# Oxidised Apolipoprotein Peptidome Characterises Metabolic Dysfunction‐Associated Steatotic Liver Disease

**DOI:** 10.1111/liv.16200

**Published:** 2025-01-17

**Authors:** Gabriele Mocciaro, Amy L. George, Michael Allison, Mattia Frontini, Isabel Huang‐Doran, Frank Reiman, Fiona Gribble, Julian L. Griffin, Antonio Vidal‐Puig, Vian Azzu, Richard Kay, Michele Vacca

**Affiliations:** ^1^ Roger Williams Institute of Liver Studies Foundation for Liver Research London UK; ^2^ Institute of Metabolic Science Metabolic Research Laboratories Addenbrooke's Hospital Cambridge UK; ^3^ Liver Unit, Cambridge NIHR Biomedical Research Centre Cambridge University Hospitals NHS Foundation Trust Cambridge UK; ^4^ Faculty of Health and Life Sciences, Clinical and Biomedical Sciences University of Exeter Medical School Exeter UK; ^5^ The Rowett Institute, Foresterhill Campus University of Aberdeen Aberdeen UK; ^6^ University of Bari "Aldo Moro" Department of Interdisciplinary Medicine, Clinica Medica "C. Frugoni" Bari Italy

**Keywords:** insulin resistance, liquid chromatography‐mass spectrometry, metabolic dysfunction‐associated steatotic liver disease, peptidomics, proteomics

## Abstract

**Background:**

Metabolic Dysfunction‐Associated Steatotic Liver Disease (MASLD) encompasses a spectrum of histological conditions ranging from simple steatosis to fibrosing steatohepatitis, and is a risk factor for cardiovascular diseases (CVD). While oxidised apolipoproteins A and B have been linked to obesity and CVD, the association between other oxidised apolipoproteins and MASLD is yet to be established. To fill this gap, we characterised the circulating serum peptidome of patients with MASLD.

**Methods:**

We studied the serum of 87 biopsy‐confirmed MASLD patients and 20 age‐ and sex‐matched control (CTRL) subjects. We first employed an untargeted LC‐MS/MS peptidomics approach (9 CTRL, 32 MASLD) to identify key hits differentially modulated, and subsequently validated the most relevant findings through targeted peptidomics in an enlarged study population (87 MASLD and 20 CTRL).

**Results:**

Untargeted serum peptidomics identified several oxidised apolipoprotein peptide fragments, including ApoE and ApoC‐III, significantly upregulated in MASLD compared to CTRL. Specifically focusing on the oxidative status of intact ApoC‐III, studied through its major glycoforms (ApoC‐III_0_, ApoC‐III_i_ and ApoC‐III_ii_), we observed a marked reduction in non‐oxidised forms of these circulating peptides alongside substantially increased levels of their oxidised proteoforms in MASLD versus controls (but not within the disease stages). Oxidised ApoE and ApoC‐III peptide fragments were also significantly correlated with obesity, insulin resistance, dyslipidaemia and transaminases, suggesting a potential link between circulating apolipoprotein oxidation and systemic/hepatic metabolic dysfunction.

**Conclusion:**

Our data reveals a previously unreported oxidised apolipoprotein profile associated with MASLD. The functional and clinical implications of these findings warrant further mechanistic investigation.

**Summary**

- Metabolic dysfunction‐associated steatotic liver disease (MASLD) is defined as an accumulation of fat in the liver in the presence of metabolic disorders (e.g. Type 2 diabetes).
- MASLD affects about one‐third of the adult population worldwide, and up to 40% of these individuals have a progressive form of the disease that can advance to severe complications (cirrhosis, cardiovascular disease and cancer).
- Non‐invasive tests to identify patients at risk for the disease and/or its complications are lacking and urgently needed.
- Here, we focused on a specific process occurring in MASLD (protein oxidation) by using advanced analytical platforms (liquid chromatography‐mass spectrometry) to examine a wide range of circulating serum proteins/peptides in MASLD patients and healthy subjects.
- We identified a previously unreported series of oxidised serum proteins (apolipoproteins), which were elevated in MASLD patients, correlated with the metabolic features of the disease, and effectively distinguished MASLD patients from controls.
- Although these findings require further validation, they could pave the way for a novel set of accurate biomarkers to screen at‐risk MASLD patients, and to identify those who may benefit from a deeper diagnostic evaluation.

## 1 Introduction

Metabolic dysfunction‐associated steatotic liver disease (MASLD), formerly known as non‐alcoholic fatty liver disease (NAFLD), has an estimated worldwide prevalence of about 30% [1]. MASLD covers a spectrum of histological features ranging from simple steatosis (MASL) to steatohepatitis (MASH), an inflammatory and fibrosing form of the disease which can lead to cirrhosis, liver failure and hepatocellular carcinoma (HCC) [2, 3]. While MASLD is considered an independent risk factor for cardiovascular diseases [4], the combination of the surrounding metabolic dysfunction [insulin resistance [5], mixed dyslipidaemia: low high‐density lipoprotein cholesterol (HDL‐C), increased very low‐density lipoprotein triglycerides (VLDL‐TG) and low‐density lipoprotein cholesterol (LDL‐C)], changes in lipoprotein lipid composition [6, 7], enhanced hepatic secretion of multiple atherogenic molecules [including pro‐inflammatory (cytokines, chemokines), vasoactive and thrombogenic factors [8]], and increased oxidative stress [9] are known drivers of the atherosclerotic process, and might contribute to the high cardiovascular risk associated with MASLD.

Various mass spectrometric techniques have been adopted to study MASLD pathophysiology and to identify candidate biomarkers of the disease, by focusing on lipidomics [10] and proteomics (global study of lipids and proteins, respectively) in liver tissue [11, 12, 13] or blood [14, 15, 16, 17, 18, 19]. However, serum mass spectrometry proteomics is analytically challenging as circulating proteins are present over a wide concentration range and are dominated by high abundance proteins such as albumin [20]. As a result, enzymatic digestion of undepleted plasma identifies only the most abundant circulatory proteins [15]. Enrichment steps, for example using immunoaffinity‐based depletion approaches, are important to identify subtle changes in less abundant proteins but can suffer from low throughput and are not practical for widespread clinical use [18]. Peptidomics (the analysis of endogenous peptides with omics approaches) can supplement conventional proteomic studies to investigate differences in low‐molecular weight moieties (that are often missed in proteomic studies) by employing in vitro digestion methods [21]. Moreover, peptidomics can help the characterisation of disease related proteo‐forms or endogenous peptide abundance providing insight into abnormal protein synthesis, processing, oxidation or degradation [22]. In this study, we employed organic solvent‐based precipitation to extract both the low molecular weight proteome and peptidome, which has previously demonstrated efficiency at removing high abundance proteins for proteomic [23, 24, 25] and peptidomic analysis [26, 27].

By performing conventional bottom‐up proteomics and a complementary top–down peptidomic analysis, we found that compared to controls, the sera of patients with MASLD are characterised by enhanced apolipoprotein oxidation, and that these oxidised peptides from apolipoproteins were intimately associated with markers of metabolic dysfunction and liver necroinflammation.

## 2 Methods

### 2.1 Ethics and Study Cohort

Patients were recruited by the NAFLD Service at Cambridge University Hospitals NHS Foundation Trust, while healthy volunteers were recruited at the NIHR Cambridge BioResource (http://www.cambridgebioresource.org.uk) and the NHS Blood and Transplant Unit, Cambridge, UK. Participant enrolment was approved by NHS Research Ethics Committees (REC 06/Q0106/70; 12/EE/0040; 17/EE/0389). Study protocols followed the principles of the Declaration of Helsinki, and all participants gave written informed consent. In healthy controls, where liver biopsy was not clinically indicated, the absence of NAFLD was predicted on the basis of the non‐invasive score proposed by Kotronen et al. [28].

The MASLD cohort, originally recruited following the NAFLD clinical and histological definition (later specified), met the criteria of MASLD [29], thus we used this definition for our cohort. The latter has been previously described with minor differences due to samples availability [30]. All the patients had a clinical diagnosis of MASLD (with alternate diagnoses and aetiologies excluded), histology scored by an expert pathologist according to the NASH Clinical Research Network scoring criteria [31, 32]. For this study, histology was grouped into MASL (fatty liver without inflammation and/or ballooning), MASH (fatty liver with inflammation and ballooning, NAFLD activity score > 4) [33] with fibrosis score 0–4. Serum was collected from healthy and MASLD participants for the assessment of standard clinical laboratory tests as previously reported [30].

Untargeted serum proteomic and peptidomic analyses were undertaken on 42 male patients (comprising 9 healthy controls and 33 patients with varying degrees of MASLD as detailed in Table S1) and henceforth referred to as “untargeted cohort.”

A targeted mass spectrometric method to measure intact apolipoproteins and their fragments (with and without oxidised methionines) with increased throughput was then performed on the initial 42 subjects sera and additional 67 subjects (comprising an additional 11 healthy controls and 56 patients with varying degrees of MASLD). The peptide fragments, and intact peptides such as ApoC‐III glycoforms, monitored in the targeted cohort were selected based on their intensity levels, presence in the majority of the samples and inclusion of oxidised methionine(s). The triple quadrupole instrument settings were optimised to obtain the best sensitivity, selectivity and separation of similar products such as ApoC‐III glycoforms (detailed settings reported in supplementary materials). A description of the combined study populations is given in Table S2, henceforth referred to as “targeted cohort.”

### 2.2 Data Processing and Statistical Analysis

Untargeted data processing was performed using Perseus software (version 1.6.12.0) [34]. The peak area intensities of each feature from the proteomics and peptidomics PEAKS analysis were imported, on the protein level or peptide level respectively, and expressed as a ratio to the internal standard (bovine insulin). Proteomic data were transformed to logarithmic scale (Base 2) and filtered for 70% data completeness in at least one experimental group. Missing values in the proteomic dataset were not imputed. Peptidomic data were transformed to logarithmic scale (Base 2) and filtered using a reduced threshold for data completeness of 30% in at least one experimental group to avoid exclusion of circulating protein degradation products that were confirmed by manual integration. Xcalibur (v4.3.73.11, Thermo Fisher Scientific) was used to query missing values within the discovery peptidomics dataset before global data was imputed in Perseus using default settings, prior to statistical analysis. Peak area ratios of fragment peptides were mapped to their protein progenitor sequence using R version 4.2.1. Missing values in the targeted dataset were imputed based on LOD/2 (limit of detection) prior to statistical analysis [35].

Clinical data are shown as mean and standard deviation unless otherwise specified. Graphs are shown as dot plots and mean values. Comparisons of clinical data between healthy and patients with MASLD were assessed using two‐way and three‐way ANOVA controlling for sex and the presence of T2DM, followed by the Tukey HSD post hoc test to estimate the statistical significance among groups, when relevant and as detailed in the legends to tables/figures. Regarding categorical variables, a chi‐square test was employed. Proteomic and peptidomic data in the untargeted cohort were analysed using two‐way ANOVA controlling for presence of T2DM, and when more than two comparisons were made this was followed by the Tukey HSD post hoc test to estimate the statistical significance among groups. For clinical values a *p* value < 0.05 was considered significant while for proteomics and peptidomics a *p* value < 0.05 (Benjamini–Hochberg false discovery rate) was considered significant. Peptidomic data in the targeted cohort were analysed using three‐way ANOVA controlling for presence of sex and T2DM as covariates, and when more than two comparisons were made this was followed by the Tukey HSD post hoc test to estimate the statistical significance among groups. A *p* value < 0.05 (Benjamini–Hochberg false discovery rate) was considered significant. A post hoc power analysis, carried out with G*power software, was performed to assess the power of this study (proteomics and peptidomics). Variables with an effect size (f) below 0.3 (proteomics) and 0.5 (peptidomics) fell below an acceptable power level of 0.7, hence exposed to Type 2 error. All the significant variables had an optimal power (> 0.8). Univariate correlations were carried out using the Pearson correlation coefficient. Prior to statistical analysis, data were logarithmically transformed (Log2). Statistical analysis and graphs were performed with R version 4.2.1 and GraphPad Prism Version 9 (GraphPad Software, San Diego, CA).

### 2.3 Other Experimental Procedures

Detailed experimental procedures (including Tables S3 and S4) are described in the supplementary files.

## 3 Results

### 3.1 Patient Characteristics

This cohort has been previously reported [30]. However, due to small differences in sample availability among our studies, we reanalysed the main clinical features, and reported them in supplementary materials. Specifically, samples from 107 participants were employed in the present study, including 87 patients with biopsy‐confirmed MASLD and varying histological severity and metabolic impairment, and 20 healthy (age and sex matched) participants. The clinical details of the untargeted cohort (*n* = 42) and the targeted cohort (*n* = 107) are shown in Tables S1 and S2. Patients with MASLD displayed higher body mass index (BMI) and insulin resistance, some features of the metabolic syndrome (lower HDL‐C and higher TG), together with increased hepatic necro‐inflammatory markers (AST/ALT). The targeted cohort included 46 patients (53%) diagnosed with T2DM (same percentage observed in the untargeted cohort) (Tables S1 and S2). With regards to pharmacological treatments, MASLD patients were treated with metformin (*n* = 9; 28% untargeted cohort and *n* = 27; 31% targeted cohort) and statins (*n* = 13; 41%, untargeted cohort and *n* = 28; 32% targeted cohort) (Tables S1 and S2).

Within the MASLD biopsy‐confirmed population, no major clinical differences were observed stratifying the patients for disease stage, except for circulating transaminases and insulin resistance (in the targeted cohort) alongside the expected significantly different hepatic histological features (Tables S5 and S6).

### 3.2 MASLD Is Associated With Marked Differences in the Serum Proteome

In the untargeted cohort (*n* = 41), the proteomic approach highlighted 28 differentially expressed proteins in MASLD compared with controls (fold change > 1.5 and false discovery rate (FDR) < 0.05), functionally linked with lipid and lipoprotein metabolism (APOA4, APOD, APOE, APOL1), antioxidant activity (ALB, APOA4, HPR, APOE), cellular oxidant detoxification (ALB, HBB, HBD, APOE, HBA1) and immune response (C3, VTN, IGKV2D‐29, IGKC, HPR, IGKV3‐11, IGHA1, IGLV3‐19, IGKV1D‐12) (Figure S1 A,B). Of interest, presence of T2DM (used as a covariate) was not significantly influencing these proteins (Table S7).

The proteomic panel largely confirmed previous findings investigating proteomics biomarkers for NAFLD [36]. To better understand the extent to which these proteins related to metabolic impairment, we correlated the significantly different proteins from Figure S1 A with metabolic clinical data (Figure S1 C). Pearson correlation analysis showed that the selected proteins strongly associated with BMI, insulin resistance (HOMA2‐IR), and transaminase levels (AST, ALT; Figure S1 C). Across the MASLD spectrum, after multiple comparison correction, we did not observe significant proteomics differences (Table S8) suggesting that disease progression has limited impact on these hits that are already modulated in the early stages of the disease. However, given the relatively small sample size, we cannot exclude that a Type 2 error affected our ability to detect disease‐stage associated differences across the MASLD spectrum in some of the proteins that have been highlighted by larger studies as stepwise regulated by disease stage [37, 38].

Taken together, the circulating proteome points to a deregulation of lipoprotein metabolism, oxidative status, and immunological activation as early events occurring in MASLD. These dysregulated processes strongly correlate with systemic metabolic impairment (e.g. insulin resistance) and markers of liver damage.

### 3.3 Untargeted Serum Peptidomics Highlights Enhanced Apolipoproteins Oxidation in MASLD

During proteomic analysis, intact circulating proteins are cleaved with trypsin, specifically at the C‐terminus of arginine and lysine residues to produce tryptic peptides of similar lengths that permit indirect measure of total protein abundance (Figure S2A). However, insight into endogenous protein processing on significant proteolytic events is lost with such an analysis, as levels of tryptic peptides produced in vitro swamp the signal for naturally occurring peptides. To better study endogenous protein modifications, we analysed the same samples using a peptidomic approach which retains the structure of circulating peptides and proteins, therefore enabling a “proteoform analysis”, with particular focus on the low molecular weight (LMW) apolipoproteins. Visualisation of the endogenous circulating peptides mapped to their protein sequence revealed extensive peptide laddering and post‐translational modifications (methionine oxidation) enriched in MASLD compared to controls (Figure S2B). The peptide component of MASLD patient's samples was notably more complex than controls, as clearly reflected in the total ion chromatograms of the two sample types (Figure S3A,B). Technical methodological details and quality controls of these analysis are reported in Supplementary Note 1.

**FIGURE 1:**
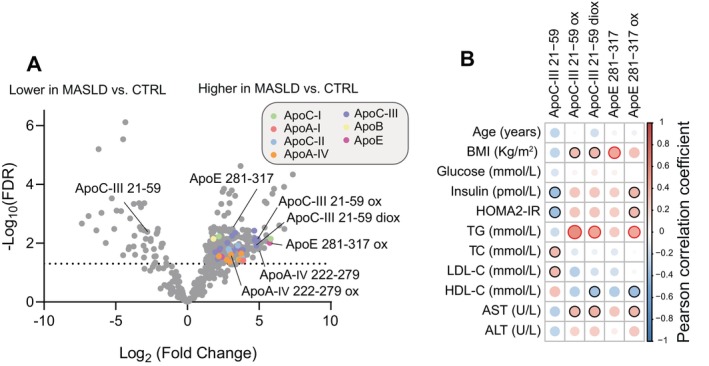
Untargeted serum peptidomics in healthy volunteers and patients with MASLD. (A) Volcano plot showing differences in serum peptides of MASLD and controls in the untargeted cohort (*n* = 42), highlighting oxidised apolipoproteins peptides. Statistical significance was assessed using two‐way ANOVA using the disease state and presence of Type 2 diabetes as covariates; a *p* value < 0.05 (Benjamini–Hochberg false discovery rate) was considered significant (B) Heatmap representing a correlation matrix among selected serum peptides and clinical data in healthy volunteers and MASLD patients: colour represents the Pearson correlation coefficient (red: positive; blue: negative), and the size of the circle represents significance (black bold borders highlight correlations with *p* < 0.05; red bold borders highlight correlations with *p* < 0.01).

The untargeted peptidomic analysis (*n* = 41) showed that 353 peptides, assigned to 40 protein groups, were differentially abundant between the MASLD and the controls (Log2 fold change > 1.5 & FDR < 0.05) (Figure 1A). Of them, 31% were peptide fragments belonging to apolipoproteins ApoA‐IV, ApoC‐III, ApoA‐I, ApoA‐II, ApoC‐I, ApoC‐II and ApoE, many of which displayed an oxidised state (Table S9). Of interest, and in line with the proteomic data, T2DM did not influence the levels of these peptides. Because of the relevance of ApoC‐III (inhibition) and ApoE (enhancement) in apolipoprotein B lipoproteins clearance/homeostasis alongside their role in mediating cardio‐metabolic risk [39], we focused on these two apolipoproteins (and their oxidation status). Specifically, we investigated the correlation of these peptides with clinical data (Figure 1B). Pearson correlation analysis showed that except for the non‐oxidised ApoC‐III peptide, the oxidised and di‐oxidised peptides alongside a peptide of ApoE and its oxidised form were positively correlated with BMI, triglycerides, AST and ALT and inversely correlated with HDL‐C (Figure 1B). Taken together, these data show that MASLD patients are characterised by a previously unreported enhanced serum apolipoprotein oxidation state and that these peptides are correlated with key clinical features of MASLD.

### 3.4 Targeted Serum Peptidomics Confirmed an Enhanced Oxidation of ApoC‐III and ApoE Peptides in MASLD

**FIGURE 2:**
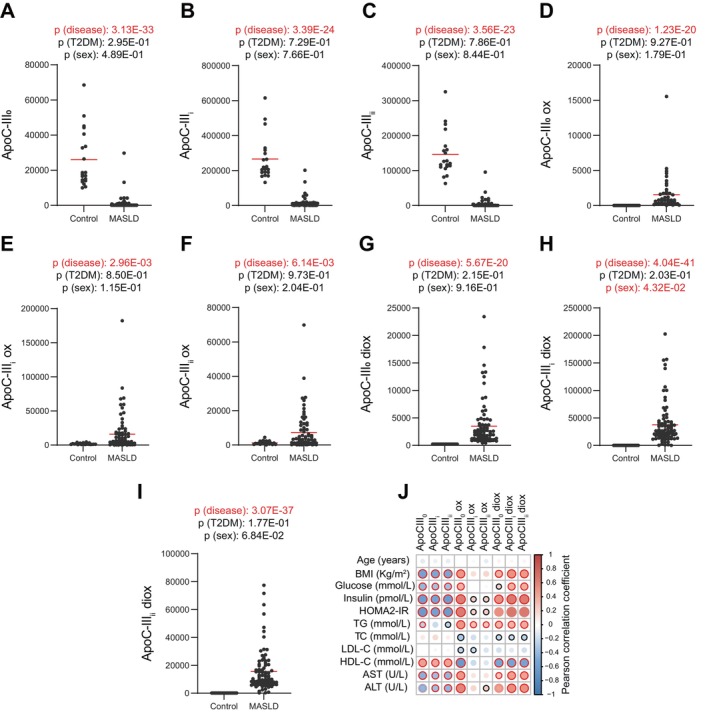
Targeted serum peptidomics in healthy volunteers and patients with MASLD. (A–C) ApoC‐III glycosylation proteoforms, and (D–I) their mono and di‐oxidised state in MASLD compared to controls in the validation cohort (*n* = 107). Statistical significance was assessed using three‐way ANOVA using the disease state, presence of Type 2 diabetes (T2DM) and sex as covariates; a *p* value < 0.05 was considered significant. (J) Heatmap representing a correlation matrix among serum glycosylated proteoforms (oxidised and non‐oxidised) and clinical data in healthy volunteers and MASLD patients: Colour represents the Pearson correlation coefficient (red: positive; blue: negative), and the size of the circle represents significance (black bold borders highlight correlations with *p* < 0.05; red bold borders highlight correlations with *p* < 0.01).

**FIGURE 3:**
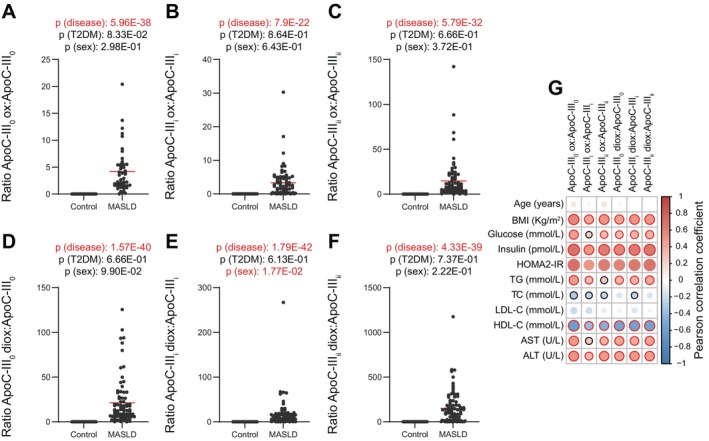
Oxidised to non‐oxidised ApoC‐III glycoforms ratios in healthy volunteers and patients with MASLD. (A–F) Ratios of oxidised to non‐oxidised ApoC‐III glycoforms in MASLD compared to controls in the validation cohort (*n* = 107). Statistical significance was assessed using three‐way ANOVA using the disease state, presence of Type 2 diabetes (T2DM) and sex as covariates; a *p* value < 0.05 was considered significant. (G) Heatmap representing a correlation matrix among serum ApoC‐III oxidised to non‐oxidised glycoforms ratios and clinical data in healthy volunteers and MASLD patients: Colour represents the Pearson correlation coefficient (red: positive; blue: negative), and the size of the circle represents significance (black bold borders highlight correlations with *p* < 0.05; red bold borders highlight correlations with *p* < 0.01).

To further investigate our initial findings, we expanded our cohort and applied a targeted peptidomics approach. Specifically, we looked at the three major ApoC‐III glycosylated proteoforms [amino acids 21–99 (numbers close to a protein/peptide indicate their primary sequence as designated in the UNIPROT database)]: namely ApoC‐III_0_ (carrying either a D‐galactose/N‐acetyl‐D‐galactosamine), ApoC‐III_i_ and ApoC‐III_ii_ (carrying one or two additional sialic acid moieties attached to the galactosamine) [40]. Moreover, we also investigated the previously identified ApoC‐III 21‐59 and ApoE 281‐317 (fragments of the whole protein generated post‐translationally), and their oxidated counterparts (Figure 2 and Figure S4). Compared to controls, patients with MASLD displayed significantly lower ApoC‐III proteoforms (ApoC‐III_0_, ApoC‐III_i_ and ApoC‐III_ii_; Figure 2A–C). On the contrary, the oxidised and di‐oxidised forms of these proteins [ApoC‐III_0_ (mono and di‐oxidised), ApoC‐III_i_ (mono and di‐oxidised), and ApoC‐III_ii_ (mono and di‐oxidised)] were significantly higher in MASLD compared to controls (Figure 2D–I) suggesting a generalised oxidation process at the level of ApoC‐III irrespective of the proteoform investigated. In agreement with the untargeted cohort, the non‐oxidised fragment ApoC‐III 21‐59 was significantly lower, while its oxidised and di‐oxidised forms were higher in MASLD compared to controls (Figure S4A–C). Moreover, aligning with the proteomics data (higher ApoE in MASLD compared to controls) the ApoE 281‐317 peptide and its oxidised form were significantly higher in MASLD vs. controls (Figure S4D,E). Pearson correlation analysis showed a remarkable negative correlation between BMI, HOMA2‐IR, triglycerides and liver enzymes with non‐oxidised ApoC‐III proteoforms while their oxidised counterparts were significantly positively correlated with these clinical paraments, suggesting a close link between the oxidative state of these proteoforms and metabolic health (Figure 2J). To study the differences in oxidised apolipoproteins proportionally to their non‐oxidised forms, we calculated the ratios of the oxidised ApoC‐III_0_, ApoC‐III_i_, ApoC‐III_ii_, ApoC‐III 21‐59 and ApoE 281‐317 to each of their non‐oxidised variants (Figure 3 and Figure S4). As expected for the ApoC‐III proteoforms and the fragment ApoC‐III 21‐59, their oxidised to non‐oxidised ratios were significantly higher in MASLD compared to controls (Figure 3A–F and Figure S4F,G), suggesting that lower levels of non‐oxidised ApoC‐IIIs could be due, at least in part, to an enhanced oxidation process (although the functional implications of these changes remain to be elucidated). Less expected was the significantly higher ratio of the ApoE 281‐317 oxidised to non‐oxidised peptide (since both oxidised and non‐oxidised forms were higher in MASLD vs. controls; Figure S4H). The latter suggests that ApoE and its peptide are not only increased in the circulation in MASLD patients but are also more oxidised. The oxisided to non‐oxidised forms were positively significantly correlated with BMI, HOMA2‐IR, AST, ALT and inversely correlated with HDL‐C (Figure 3G and Figure S4I).

As anticipated from the clinical tables, none of the oxidised peptides were substantially different when looking across MASH progression (data not shown), highlighting that these differences are an early event in the MASLD development, strongly associated with metabolic impairment, but not with disease progression.

**FIGURE 4:**
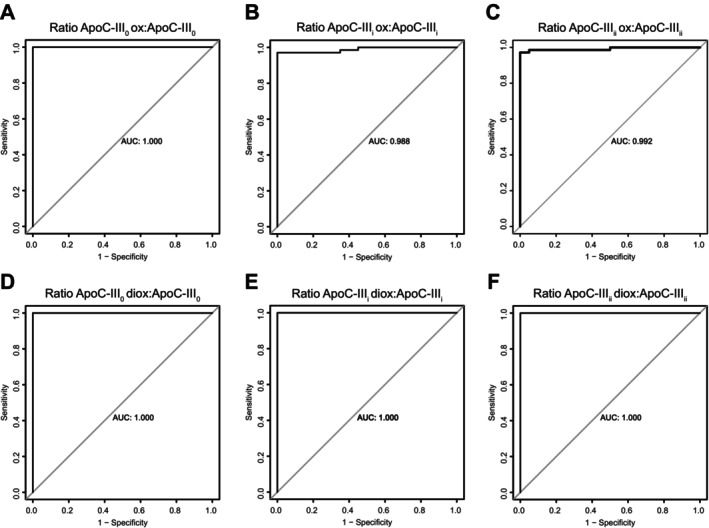
(A–F) Performance of oxidised to non‐oxidised apolipoproteins ratios as biomarkers of MASLD. Receiver‐operating characteristic (ROC) curve analysis of ApoC‐III oxidised to non‐oxidised glycoforms ratios of MASLD and controls in the targeted cohort (*n* = 107).

Last, to assess the performance of oxidised to non‐oxidised ApoC‐III proteoforms ratios as biomarkers of MASLD, we performed receiver‐operating characteristic (ROC) curve analysis (Figure 4A–F). The oxidised to non‐oxidised ApoC‐III proteoforms ratios showed excellent discriminatory capabilities between MASLD and controls. Specifically, ApoC‐III_0_, oxidised to non‐oxidised ratio displayed a ROC area under the curve (AUC) of 1.000 (95% CI = 1.000–1.000) (Figure 4A), ApoC‐III_i_ and ApoC‐III_ii_ oxidised to their non‐oxidised ratios showed a ROC AUC of 0.988 (95% CI = 0.971–1.000) and AUC: 0.992 (95% CI = 0.978–1.000), respectively (Figure 4B,C). The di‐oxidised to non‐oxidised ratios of ApoC‐III_0_, ApoC‐III_i_ and ApoC‐III_ii_ showed a ROC AUC of 1.000 (95% CI = 1.000–1.000) (Figure 4D–F). Despite the striking discriminatory capabilities of these peptids, these results require further validation before being considered candidate biomarkers for MASLD.

## 4 Discussion

MASLD is a multifactorial disease spectrum characterised by an accumulation of intrahepatic fat, due to an imbalance in hepatic lipid fluxes (enhanced influx from adipose tissue via free fatty acids, diet and hepatic *de novo* lipogenesis) coupled with reduced lipid export and catabolism [41]. Since the liver produces most of the plasmatic proteins [42], and there is a lack of non‐invasive biomarkers to stage the liver disease state, plasma proteomic approaches are currently being widely tested to better characterise this condition.

In this study, the combination of a low molecular weight proteomic approach with a peptidomic analysis allowed us to identify multiple biologically relevant proteins and novel peptides characterising MASLD. Our initial proteomics analysis largely confirmed previous findings in patients with NAFLD [15, 16, 43, 44]; however, we did not detect significant proteomic differences within the MASLD histological spectrum. This conflicts with other studies showing proteomic signatures associated with MASH progression [37, 38], probably due to differences among studies in terms of technology used to detect the proteome, sample size, and the number of proteins investigated.

To date, very few sera/plasma peptidomic studies have been performed on MASLD/NAFLD patients [36], and none of them have assessed the oxidation state across different apolipoproteins. One peptidomic study identified peptides from Complement C3 and fibrinogen in serum as potential discriminators of twins with/without NAFLD [45]. The study however only focussed on peptides up to 3.5 kDa, and therefore would have missed detection of larger peptides that we identified in our cohort. The serum/plasma peptidome, unlike their equivalent proteome, usually does not contain analytes circulating at high concentrations, mainly due to their high rates of peptide clearance (through the kidney) and degradation (by peptidases). Therefore, the fact that we detected several apolipoprotein fragments was unexpected. A possible reason for their high serum level is that these apolipoprotein‐derived peptides could still be interacting with the lipid surface of the lipoproteins, preventing them from being cleared. This draws similarities to the half‐life extending properties of lipidated peptide analogue drugs such as liraglutide and semaglutide [46, 47].

Oxidised apolipoproteins, especially ApoB and ApoA‐I, have been extensively investigated for their possible role in cardiovascular disease [48, 49], but much less is known about the oxidation status of other apolipoproteins, and their association with MASLD. Our peptidomics platform confirmed higher oxidation of ApoB (one fragment) and ApoA‐I fragments (Table S9); intriguingly, in MASLD patients' sera compared to controls, we also found substantial MASLD‐associated changes in intact ApoC‐III glycoforms and fragments of ApoC‐III and ApoE, as well as a previously unreported increase in the oxidative state of these molecules.

ApoC‐III has been extensively studied for its crucial role in TG metabolism and its causal role in CVD, with supportive evidence from genetic studies to pharmacological trials [50]. ApoC‐III can be present in both ApoB‐ and ApoA‐carrying lipoproteins, with different proportions based on the health status of a subject. Specifically, in healthy subjects, ApoC‐III is mostly found in ApoA‐carrying lipoproteins (i.e. HDLs), whereas in patients with hypertriglyceridemia, it is mostly found in ApoB‐carrying lipoproteins (i.e. LDLs or VLDLs) [39]. ApoC‐III is located on the surface of lipoproteins and modulates their levels by influencing both the conversion rate to smaller and denser fractions and their direct clearance from circulation [50]. In plasma, ApoC‐III circulates predominantly as a glycosylated form, a post‐translational modification occurring in the Golgi apparatus whereby carbohydrates are enzymatically attached to specific peptide sequences [50]. While the two most common proteoforms of ApoC‐III are sialylated apolipoproteins (containing one or two molecules of sialic acid), their role as a regulator of TG metabolism is not well understood [50]. The main ApoC‐III fragment we identified in serum (Apo‐CIII 21‐59), also contained up to two oxidised methionines, while the C‐terminally cleaved peptide of ApoE (residues 281‐317) contained a single oxidised methionine. ApoC‐III is a protein that strongly interacts with the lipid surface of lipoproteins [51], therefore it is possible that cleaved ApoC‐III peptides could still retain their lipid interaction, preventing their clearance.

Different studies have reported differentially modulated ApoC‐III proteoforms in isolated lipoproteins as result of metabolic disease (MetS and T2DM) but with contrasting results (showing higher, lower and not significantly different levels of these proteins [52, 53, 54]) probably due to different strategies for normalisation (expressed as percentage to total ApoC‐III, or normalised against the lead apolipoprotein of a specific lipoprotein fraction i.e. ApoB or ApoA‐I).

Our study showed that patients with MASLD were characterised by a remarkable increase in the oxidation of ApoC‐III proteoforms compared with controls, that this is a MASLD‐specific process, without a major influence exerted by T2DM (Figure 2), and positively correlated to metabolic features of MASLD (BMI, triglycerides HOMA2‐IR, AST and ALT), and negatively correlated with HDL‐C.

Increased protein oxidation is biologically plausible since, in the pathophysiology of MASLD, there has been reported impairment of mitochondrial β‐oxidation and oxidative phosphorylation, coupled with enhanced extra‐mitochondrial oxidative processes (peroxisomal and omega‐oxidation) which lead to the formation of reactive oxygen species (ROS) in the liver [55, 56]. ROS production is further enhanced by the hepatic endoplasmic reticulum stress response [57] and possibly by heightened *de novo* lipogenesis (a key feature of MASLD), which depletes the nicotinamide adenine dinucleotide phosphate pool and cellular antioxidant defences, thus leading to enhanced ROS production [58]. Increased hepatic ROS can cause DNA, lipid, amino acid, and peptide oxidation, possibly explaining, at least in part, our findings. It is also plausible that apolipoproteins might also be oxidated in the bloodstream, due to a reduced HDL antioxidant capacity (paraoxonase1) as observed in NAFLD/MASLD compared to controls [59]. Future mechanistic studies are required to understand which pathways lead to enhanced apolipoproteins oxidation and the biological implications of these findings with regards to the possibility that enhanced ApoC‐III oxidation could lead to functional consequences in terms of lipoprotein metabolism, and MetS/MASLD complications.

We also identified MASLD‐associated changes on the C‐terminal fragment peptide from ApoE (281‐317), which is part of its lipid binding domain [60]. ApoE plays a major role in hepatic lipoprotein uptake as it interacts with the proteins involved in the endocytic clearance of lipoproteins (LDL receptor and its family members alongside heparan sulphate proteoglycans) [61]. Higher levels of ApoE have been found in NAFLD patients [15], it was thus not surprising that also its fragments are increased in MASLD. Intriguingly, also the oxidation of ApoE fragment was increased in MASLD; this could contribute to the loss of function of this apolipoprotein and delay the hepatic uptake (catabolism) of ApoB containing lipoproteins, thus potentially further promoting the hypertriglyceridemic state often observed in MASLD patients [62]. Similar to the ApoC‐III proteoforms, the two ApoE fragments (oxidised and non‐oxidised) showed striking correlation with different metabolic and necro‐inflammatory features of MASLD thus suggesting that ApoE fragments abundance is associated to multiple aspects of the disease including metabolic features and liver damage.

Finally, the ratios between oxidised and non‐oxidised proteoforms (especially those of ApoC‐III) showed a perfect discriminatory capability between MASLD and control sera, suggesting a promising role as a non‐invasive biomarker for this condition: we think that the ApoC‐III oxidised ratios could be used to refine MASLD prediction in patients with risk factors (e.g. diabetes); the combination of these biomarkers with non‐invasive predictors of fibrosis (e.g. FIB4 or Fibroscan) will restrict the number of patients to be referred for further hepatology screening and surveillance.

Our data suggest that ApoC‐III and ApoE oxidation are early events associated with MASLD development; despite correlating with liver damage, we did not observe a worsening alongside the disease spectrum thus potentially discounting the direct contribution of apolipoproteins oxidation in the progression of the disease. Moreover, the knowledge in this field is at its infancy: mechanistic studies are needed to clarify if apolipoproteins oxidation is a consequence of an enhanced oxidative status associated to MASLD (thus making oxidised apolipoproteins merely a biomarker of the disease), or rather oxidised apolipoproteins can contribute to the pathophysiology of the disease and its complications (e.g. CVD).

There are several limitations that warrant discussion. First, the study's cross‐sectional nature provides a correlation between proteins/peptides and disease state. Thus, causation cannot be drawn. Second, the contribution of oxidised apolipoproteins and their functional implication is only speculative and requires adequate mechanistic investigation in future studies. Third, our study does not address which lipoprotein (HDL, LDL, VLDL) is mostly affected by the apolipoprotein oxidation observed, but at the same time provides a picture of the overall serum apolipoprotein oxidation status irrespective of the ApoB/ApoA content, which is typically used as a normalising denominator in lipoproteins studies. Finally, the population included in this study is relatively small thus limiting the generalizability of our findings that need to be confirmed in larger cohorts with heterogeneous population (e.g. ethnicity, genetic background, etc.).

In conclusion, by using mass‐spectrometry‐based peptidomics, we show that MASLD is characterised by a previously unreported enhanced oxidised apolipoprotein peptidome, including ApoE and ApoC‐III, which were strongly associated with metabolic impairment and necro‐inflammatory features of MASLD. The functional implication of these findings alongside their potential use as a non‐invasive biomarker requires further mechanistic investigation.

## Author Contributions

M.V., R.K. and V.A. conceived and designed the study. G.M., A.G., M.V., V.A. and R.K. wrote the manuscript. M.A., V.A., G.M., M.V. and M.F. recruited the participants. A.G., R.K., F.R. and F.G. performed the peptidomic analyses. G.M., A.G. and R.K. performed statistical analyses and prepared the figures. V.A., R.K., M.A., M.V., F.R., F.G., A.V.P. and J.L.G. acquired funding and or provided resources for the study. All the authors provided useful criticism during the study, critically reviewed the manuscript, and agreed to the published version of the manuscript.

## Conflicts of Interest

V.A. is an employee of AstraZeneca and may hold company shares/stocks. M.V. consults for and receives research funding from Boehringer Ingelheim. They declare no additional competing interests.

## Supporting information


Data S1.

## Data Availability

The data that support the findings of this study are openly available in ProteomeXchange Consortium at https://www.proteomexchange.org/, reference number PXD052061.
