# Supplementary material for: Oxidised Apolipoprotein Peptidome Characterises Metabolic Dysfunction‐Associated Steatotic Liver Disease
Source: Liver Int. 2025 Jan 17;45(2):e16200. doi: 10.1111/liv.16200 (PMC11740006; doi:10.1111/liv.16200)
Supplement: Supplementary file 1 — Data S1. [file LIV-45-0-s001.docx]

# Oxidised Apolipoprotein Peptidome Characterises Metabolic Dysfunction-Associated Steatotic Liver Disease

Gabriele Mocciaro^1^*, Amy L. George^2^*^+^, Michael Allison^3^, Mattia Frontini^4^ ,Isabel Huang-Doran^2^, Frank Reiman^2^, Fiona Gribble^2^, Julian L. Griffin^5^, Antonio Vidal-Puig^2^, Vian Azzu^2,3#§^, Richard Kay^2#^, Michele Vacca^1,6#^

***Joint first authors ^#^Joint senior and corresponding authors**

1 Roger Williams Institute of Liver Studies, Foundation for Liver Research, London SE5 9NT, UK

2 Institute of Metabolic Science Metabolic Research Laboratories, Addenbrooke’s Hospital, Hills Road, Cambridge CB2 0QQ, UK

3 Liver Unit, Cambridge NIHR Biomedical Research Centre, Cambridge University Hospitals NHS Foundation Trust, Cambridge, UK

4 Faculty of Health and Life Sciences, Clinical and Biomedical Sciences, University of Exeter Medical School, RILD Building, Barrack Road, Exeter, EX2 5DW, UK

5 The Rowett Institute, Foresterhill Campus, University of Aberdeen, Aberdeen AB25 2ZD, UK

6 Aldo Moro University of Bari, Department of Interdisciplinary Medicine, Clinica Medica "C. Frugoni", Bari 70124, Italy

^+^ Current address: Newcastle University, Faculty of Medical Sciences, Biosciences Institute, Framlington Place, Newcastle Upon Tyne, NE2 4HH, UK.

^§^ Current address: Translational Science & Experimental Medicine, Research and Early Development Cardiovascular, Renal and Metabolism (CVRM), AstraZeneca, Biomedical Campus, 1 Francis Crick Ave, Cambridge CB2 0AA, UK.

**Keywords:** Metabolic Dysfunction-Associated Steatotic Liver Disease; insulin resistance; peptidomics; proteomics; liquid chromatography-mass spectrometry.

^#^Corresponding author. Aldo Moro University of Bari, Department of Interdisciplinary Medicine, Clinica Medica “C. Frugoni”, Bari, 70124, Italy. E-mail: [michele.vacca@uniba.it](mailto:michele.vacca@uniba.it) (M. Vacca).

^#^Corresponding author. Institute of Metabolic Science Metabolic Research Laboratories, Addenbrooke’s Hospital, Hills Road, Cambridge CB2 0QQ, UK. E-mail: [rgk27@medschl.cam.ac.uk](mailto:rgk27@medschl.cam.ac.uk) (R. Kay).

^#^Corresponding author. Institute of Metabolic Science Metabolic Research Laboratories, Addenbrooke’s Hospital, Hills Road, Cambridge CB2 0QQ, UK. E-mail: [va220@medschl.cam.ac.uk](mailto:va220@medschl.cam.ac.uk) (V. Azzu).

**Supplementary materials**

Supplementary experimental procedures

Supplementary Files:

1. Supplementary Note 1

2. Supplementary Tables 1-9

3. Supplementary Figures 1-4

Supplementary references

**Supplementary experimental procedures**

## **Chemicals and reagents**

Acetonitrile (ACN) (A955-212), formic acid (FA) (A117-50), guanidine hydrochloride, LC-MS grade water (w/0112/17) and methanol (M/4056/17) were purchased from Fisher Scientific (Thermo Fisher Scientific, Hemel Hempstead, UK). Iodoacetamide (IAA) (I1149), dithiothreitol (DTT) (43819), ammonium bicarbonate (ABC) and dimethyl sulfoxide (DMSO) were obtained from Sigma-Aldrich (Sigma-Aldrich, Gillingham, UK). Acetic acid (0714-500ML) was purchased from VWR (VWR, Lutterworth, UK). Trypsin Gold (V528A) was sourced from Promega (Promega, Southampton, UK).

## **Low molecular weight proteome extraction**

Samples were thawed on ice and 25 µL was transferred to a fresh well in a 96 well plate. An equal volume of 6M guanidine hydrochloride was added and the plate mixed thoroughly before precipitation was performed with 300 µL of 75% ACN in water (v/v) with bovine insulin at 50 ng/mL. Plates were centrifuged at 2900g for 10 mins at 4˚C and the supernatants transferred to clean Eppendorf Lobind protein plates and evaporated under oxygen free nitrogen at 40 ˚C on a Biotage SPE dry 96 (Uppsala, Sweden). The residue was reconstituted into 200 µL of 0.1% FA in water (v/v) and subjected to SPE as described previously (*1*) Extracts were resuspended in 75 µL of 50 mM ABC with 10 mM DTT and incubated at 60˚C for one hour. Free cysteine residues were capped by the addition of 20 µL of 100 mM IAA in 50 mM ABC solution and left in the dark for 30 mins at room temperature. Modified porcine trypsin was added (10 µL of a 0.1 µg/µL solution) to digest samples for proteomic analysis and incubated overnight at 37˚C. Prior to sample analysis (10 µL injected), 20 µL of 1% FA was added to stop the digestion.

## **Peptidomic extraction**

To enrich for the circulating peptidome, a modified extraction method was applied, where 10 µL sample volume (or 9 µL for subsequent targeted analysis) was transferred to 96 well plates and organic solvent precipitation was performed using 60 µL of 80% ACN in water (v/v) with bovine insulin at 5 ng/mL. The plate was centrifuged, the supernatant removed and 10 µL of DMSO added prior to solvent evaporation as previously described. Peptidomic samples were reconstituted, reduced, and alkylated as described previously, however no enzymatic digestion was performed, and sample (10 µL) was analysed after addition of 20 µL 1% FA.

## **Nano LC-MS/MS analysis**

All LC-MS/MS analyses were performed on an ThermoScientific Ultimate 3000 nano LC system (San Jose, USA) coupled to a ThermoScientific Q-Exactive plus Orbitrap mass spectrometer. The loading flow rate was 30 µL per minute (2% ACN in water with 0.1% FA v/v) and held for 15 mins to load the sample onto a Thermo 0.3 x 5 mm trap column. The trap was then switched in line with the ThermoScientific Easyspray nano column 250 x 0.075 mm flowing at 300nL/min. The mobile phases were A: 0.1% FA in water (v/v) and B: 0.1% FA (v/v) in 80:20 ACN/water.

*Proteomic analysis:* Initial conditions were 2.5% B and held for 15 mins before increasing to 50% B over 90 mins. The column was washed with 90% B for 20 mins before returning to starting conditions for a further 20 mins, totalling an entire run time of 130 mins.

*Peptidomic analysis:* To obtain data on the more hydrophobic intact apolipoproteins, a modified gradient separation was performed over a 100-minute method, where %A was increased from 2.5% to 55% over 60 mins, before a 20-minute wash and return to starting conditions for a final 20 mins.

Positive nano electrospray analysis was performed using a spray voltage of 1.7 kV, the tune settings for the mass spectrometer used an S‐lens setting of 70 V to target peptides of higher *m/z* values. A full scan range of 400–1600 *m/z* was performed at a resolution of 75,000 before the top 10 ions of each spectrum were selected for MS/MS analysis. Existing ions selected for fragmentation were added to an exclusion list for 30s.

## **Validation of in-vivo and ex-vivo oxidation of ApoC-III isoforms**

To investigate whether the oxidation of ApoC-III protein isoforms could occur after serum collection (as an experimental artefact), control serum was subjected to up to three freeze thaw cycles and a 24h bench top stability test. All samples were extracted and analysed using the modified peptidomics methodology as described in the manuscript and the level of oxidation, and formation of degraded apolipoprotein peptides was assessed.

## **High flow SRM LC-MS/MS analysis**

A selected reaction monitoring (SRM) method was developed to measure a panel of intact apolipoproteins and their fragments (with and without oxidised methionines) that were significantly differentially expressed in the peptidomics analyses. Targeted analysis was performed on a Waters M-Class UPLC system using a 1.0 x 5 mm T3 HSS 1.8 µm particle column held at a temperature of 60 ˚C. The flow rate was set at 25 µL per minute with mobile phases A: 0.1% FA in water (v/v) and B: 0.1% FA in ACN (v/v) and started at 20% B. Peptides were eluted over a 6-minute gradient to 55% B, the column washed at 85% B for 1.4 mins before returning to starting conditions for a total method time of 10 mins. The SRM transitions that were monitored, and collision energy settings are included in **Supplementary Tables 3 and 4**. The source voltage was 3.0 kV, the cone voltage was 30 V, gas 600 L/h, SRM dwell times for all peptide fragments were 20ms whilst the intact apolipoproteins had a dwell time of 10ms.

## **Protein identification and quantitation**

Global LC-MS/MS data was searched against the Uniprot database (downloaded 29th November 2018) using PEAKS 8.5 (BSI, Waterloo, Canada) with a human filter applied. The proteomics search parameters included trypsin digest, a fixed carbamidomethylation on cysteine residues and variable methionine oxidation. The peptidomics search used a no-enzyme setting with carbamidomethylation on cysteine residues and variable modifications of methionine oxidation, N-terminal pyroglutamate, N-terminal acetylation and C-terminal amidation. In both cases, the *m/z* tolerances were 10ppm accuracy for precursor ions and 0.05 m/z for product ion fragments. Only proteins identifications with a 1% false discovery rate (FDR) and at least one unique peptide were included in further analyses. Manual interrogation of raw discovery data was performed using Xcalibur (v4.3.73.11, Thermo Fisher Scientific) to identify targeted transitions of biomarker candidates for SRM method development. Peptide peak areas from the SRM analysis were manually integrated using the TargetLynx program within Masslynx (v 4.2, Waters).

**Note**

**Supplementary Note 1.**

Because of the limitations of the PEAKS software (only peptides under 65 amino acid residues can be matched), intact peptides such as ApoC-III were manually analysed in the raw data. This analysis identified the presence of intact and oxidised apolipoproteins such as ApoC-I, ApoC-II, ApoC-III and ApoA-2. To ensure experimentally induced peptide oxidation was not a confounding factor, serum samples were subject to repeated freeze thaws cycles which produced a minimal 0.1% increase in oxidation of the total intact ApoC-III protein signal, a ratio significantly smaller than that observed in the MASLD samples **(Supplementary Figure 3c)**. Importantly, it should be noted that all samples were handled uniformly, and these findings emphasise that the level of oxidation induced by sample manipulation is negligible compared to the biological changes observed.

# Tables

**Supplementary Table 1. Clinical characteristics of the untargeted cohort.**

|  | **Healthy controls n = 9** | **MASLD  n = 32** | **p-value** | |
| --- | --- | --- | --- | --- |
|  |  |  | **T2DM Disease** | |
| Age (years) | 47 ± 12 | 52 ± 13 | 1.55E-01 | 6.17E-01 |
| BMI (kg/m^2^) | 25 ± 3 | 31 ± 3 | **1.56E-02** | **2.79E-04** |
| Glucose (mmol/L) | 5 ± 0.7 | 6.4 ± 1.5 | **1.66E-06** | 3.27E-01 |
| Insulin (pmol/L) | 42 ± 20 | 113 ± 59 | 4.43E-01 | **1.02E-03** |
| HOMA2-IR | 0.8 ± 0.3 | 2.2 ± 1.1 | 2.12E-01 | **9.45E-04** |
| TG (mmol/L) | 1.1 ± 0.6 | 1.6 ± 0.6 | 9.59E-01 | **3.72E-02** |
| TC (mmol/L) | 4.6 ± 0.7 | 4 ± 0.8 | 9.63E-01 | **3.40E-02** |
| LDL-C (mmol/L) | 2.8 ± 0.5 | 3.1 ± 0.8 | 7.94E-01 | 1.10E-01 |
| HDL-C (mmol/l) | 1.3 ± 0.3 | 0.9 ± 0.2 | 4.27E-01 | **2.52E-04** |
| AST (U/L) | 24 ± 7 | 48 ± 28 | 6.77E-01 | **1.16E-02** |
| ALT (U/L) | 38 ± 10 | 71 ± 35 | 4.45E-01 | **1.02E-03** |
| Steatosis | - | 1.6 ± 0.8 | - | - |
| Inflammation | - | 1.5 ± 0.7 | - | - |
| Ballooning | - | 0.9 ± 0.7 | - | - |
| NAS | - | 4 ± 1.5 | - | - |
| Fibrosis | - | 1.8 ± 1.2 | - | - |
| T2DM, n (%) | 0 | 17 (53) | - | **1.36E-02^#^** |
| Statin, n (%) | 0 | 13 (41) | - | **7.63E-03^#^** |
| Metformin, n (%) | 0 | 9 (28) | - | **1.69E-02^#^** |

*Data are mean ± SD and n (%). Statistical tests are two-way ANOVA with diabetes (T2DM) as covariate, and Pearson χ2 test #, as appropriate. A p-value <0.05 was considered significant. MASLD = Metabolic Dysfunction-Associated Steatotic Liver Disease, BMI = Body Mass Index, HOMA2-IR = Homeostasis Model Assessment 2 of Insulin Resistance, TG = Triglycerides, TC = Total Cholesterol, HDL-C = High-density lipoprotein cholesterol, LDL-C = Low-density lipoprotein cholesterol, AST = Aspartate aminotransaminase, ALT = Alanine aminotransaminase; NAS: NAFLD activity score.*

**Supplementary Table 2. Clinical characteristics of the targeted cohort.**

|  | **Healthy  controls n = 20** | **MASLD n = 87** | **p-value** | | |
| --- | --- | --- | --- | --- | --- |
|  |  |  | **T2DM Sex Disease** | | |
| Sex (M/F) | 9/11 | 54/33 | - | - | 1.62E-01^#^ |
| Age (years) | 54 ± 13 | 55 ± 11 | **2.17E-02** | **1.14E-03** | 8.96E-01 |
| BMI (kg/m^2^) | 25 ± 4 | 32 ± 5 | **1.10E-04** | 2.52E-01 | **1.50E-07** |
| Glucose (mmol/L) | 4.8 ± 0.7 | 6.7 ± 2.2 | **1.05E-11** | 4.01E-01 | 9.82E-02 |
| Insulin (pmol/L) | 38 ± 20 | 127 ± 72 | **8.60E-04** | 1.80E-01 | **5.10E-05** |
| HOMA2-IR | 0.7 ± 0.3 | 2.5 ± 1.4 | **3.86E-05** | 2.54E-01 | **4.19E-05** |
| TG (mmol/L) | 1.1 ± 0.4 | 1.8 ± 1.3 | 4.98E-01 | 2.85E-01 | **2.72E-02** |
| TC (mmol/L) | 4.8 ± 0.8 | 4.4 ± 1.2 | 1.74E-01 | 8.63E-02 | 4.65E-01 |
| LDL-C (mmol/L) | 2.7 ± 0.6 | 2.6 ± 1 | 2.03E-01 | 3.74E-01 | 6.98E-01 |
| HDL-C (mmol/l) | 1.6 ± 0.5 | 1 ± 0.3 | 6.32E-02 | **3.64E-03** | **7.52E-08** |
| AST (U/L) | 22 ± 5 | 44 ± 24 | **2.25E-02** | 2.60E-01 | **1.27E-03** |
| ALT (U/L) | 31 ± 10 | 66 ± 34 | 8.07E-01 | **4.07E-04** | **1.65E-05** |
| Steatosis | - | 1.7 ± 0.7 | - | - | - |
| Inflammation | - | 1.6 ± 0.6 | - | - | - |
| Ballooning | - | 0.9 ± 0.8 | - | - | - |
| NAS | - | 4 ± 1.4 | - | - | - |
| Fibrosis | - | 1.6 ± 1.2 | - | - | - |
| T2DM, n (%) | 0 | 46 (53) | - | - | **1.38E-05^#^** |
| Statin, n (%) | 0 | 28 (32) | - | - | **1.38E-05^#^** |
| Metformin, n (%) | 0 | 27 (31) | - | - | **2.76E-06^#^** |

*Data are mean ± SD and n (%). Statistical tests are three-way ANOVA with diabetes (T2DM) and sex as covariates, and Pearson χ2 test #, as appropriate.* *A p-value <0.05 was considered significant. MASLD = Metabolic Dysfunction-Associated Steatotic Liver Disease, M = Male, F = Female, BMI = Body Mass Index, HOMA2-IR = Homeostasis Model Assessment 2 of Insulin Resistance, TG = Triglycerides, TC = Total Cholesterol, HDL-C = High-density lipoprotein cholesterol, LDL-C = Low-density lipoprotein cholesterol, AST = Aspartate aminotransaminase, ALT = Alanine aminotransaminase; NAS: NAFLD activity score.*

**Supplementary Table 3. Precursor (Q1) and product (Q3) ion *m/z* values, collision energy (CE) and retention times (RT) of the apolipoproteins higlighted in Figure 1.**

| **Apolipoprotein peptide fragments** | | | | |
| --- | --- | --- | --- | --- |
| Peptide | Q1 (*m/z*) | Q3 (*m/z*) | CE (eV) | RT (min) |
| ApoC-III 21-59 | 838.50 | 217.08 | 30 | 4.1 |
| ApoC-III 21-59 Oxidised | 841.70 | 217.08 | 25 | 3.5 |
| ApoC-III 21-59 Dioxidised | 844.90 | 217.08 | 25 | 2.8 |
| ApoE 281-317 | 1013.75 | 765.35 | 25 | 5.9 |
| ApoE 281-317 Oxidised | 1017.75 | 765.35 | 25 | 4.8 |
| **Intact apolipoproteins** | | | | |
| Peptide | Q1 (*m/z*) | Q3 (*m/z*) | CE (eV) | RT (min) |
| ApoC-III_0_ | 1252.89 | 217.08 | 65 | 5.37 |
| ApoC-III_0_ Oxidised | 1255.20 | 217.08 | 65 | 4.99 |
| ApoC-III_0_ Dioxidised | 1257.60 | 217.08 | 65 | 4.72 |
| ApoC-III_i_ | 1346.72 | 274.20 | 30 | 5.38 |
| ApoC-III_i_ Oxidised | 1349.10 | 274.20 | 30 | 4.98 |
| ApoC-III_i_ Dioxidised | 1351.30 | 274.20 | 30 | 4.70 |
| ApoC-III_ii_ | 1388.57 | 274.20 | 30 | 5.42 |
| ApoC-III_ii_ Oxidised | 1390.58 | 274.20 | 30 | 5.03 |
| ApoC-III_ii_ Dioxidised | 1392.90 | 274.20 | 30 | 4.76 |

## **Supplementary Table 4.** **Precursor ion *m/z* values and retention times associated with the peptides selected for further validation.**

| Peptide | Peptide *m/z* values, including 13 isotopes | Charge (*z*) | RT (mins) |
| --- | --- | --- | --- |
| ApoC-III 21-59 | 1110.04, 1110.29, 1110.54, 1110.79, 1111.04 | 4 | 53.4 |
| ApoC-III 21-59 Oxidised | 841.20, 841.40, 841.60, 841.80, 842.00 | 5 | 42.0 |
| ApoC-III 21-59 Dioxidised | 844.40, 844.60, 844.80, 845.00, 845.20 | 5 | 38.4 |
| ApoE 281-317 | 1013.25, 1013.50, 1013.75, 1014.00, 1014.25 | 4 | 56.0 |
| ApoE 281-317 oxidised | 1017.25, 1017.50, 1017.75, 1018.00, 1018.25 | 4 | 51.0 |
| Bovine insulin A-chain | 856.36, 856.69, 857.02, 857.36 | 3 | 45.7 |
| ApoC-III_0_ | 1252.89, 1253.04, 1253.18, 1253.32 | 7 | 55.8 |
| ApoC-III_0_ Oxidised | 1255.03, 1255.18, 1255.32, 1255.46 | 7 | 53.4 |
| ApoC-III_0_ Dioxidised | 1257.46, 1257.61, 1257.75, 1257.89 | 7 | 51.2 |
| ApoC-III_i_ | 1346.64, 1346.78, 1346.93, 1347.07 | 7 | 56.0 |
| ApoC-III_i_ Oxidised | 1348.93, 1349.07, 1349.21, 1349.35 | 7 | 53.5 |
| ApoC-III_i_ Dioxidised | 1351.21, 1351.35, 1351.50, 1351.64 | 7 | 51.4 |
| ApoC-III_ii_ | 1388.23, 1388.37, 1388.51, 1388.65 | 7 | 56.7 |
| ApoC-III_ii_ Oxidised | 1390.51, 1390.65, 1390.79, 1390.94 | 7 | 54.1 |
| ApoC-III_ii_ Dioxidised | 1392.80, 1392.94, 1393.08, 1393.22 | 7 | 51.9 |

**Supplementary Table 5. Clinical characteristics of patients with MASL, MASH F0-2 or MASH F3-4 (untargeted cohort).**

|  | **MASL n = 11** | **MASH F0-2 n = 12** | **MASH F3-4 n = 9** | **p-value** | |
| --- | --- | --- | --- | --- | --- |
|  |  |  |  | **T2DM Disease** | |
| Age (years) | 48 ± 12 | 51 ± 14 | 60 ± 10 | 3.14E-01 | 1.61E-01 |
| BMI (kg/m^2^) | 30 ± 3 | 32 ± 4 | 30 ± 3 | 4.60E-01 | 7.62E-01 |
| Glucose (mmol/L) | 5.7 ± 0.7 | 6.8 ± 1.9 | 6.5 ± 1.6 | **2.37E-04** | 7.86E-01 |
| Insulin (pmol/L) | 101 ± 38 | 121 ± 76 | 118 ± 57 | 2.38E-01 | 3.35E-01 |
| HOMA2-IR | 1.9 ± 0.7 | 2.4 ± 1.5 | 2.2 ± 0.9 | 4.29E-01 | 3.24E-01 |
| TG (mmol/L) | 1.6 ± 0.7 | 1.7 ± 0.7 | 1.5 ± 0.5 | 2.57E-01 | 3.63E-01 |
| TC (mmol/L) | 3.7 ± 0.8 | 4.2 ± 0.7 | 4.2 ± 0.9 | 4.55E-01 | 2.51E-01 |
| LDL-C (mmol/L) | 2.1 ± 0.9 | 2.6 ± 0.7 | 2.5 ± 1 | 3.60E-01 | 2.34E-01 |
| HDL-C (mmol/l) | 0.9 ± 0.2 | 0.9 ± 0.2 | 1 ± 0.4 | 1.77E-01 | 5.42E-01 |
| AST (U/L) | 36 ± 12 | 50 ± 35 | 62 ± 27**^a^** | 1.88E-01 | **2.97E-02** |
| ALT (U/L) | 70 ± 38 | 66 ± 34 | 80 ± 36 | **3.87E-02** | 3.24E-01 |
| Steatosis | 1.4 ± 0.7 | 1.8 ± 0.8 | 1.7 ± 0.9 | 5.02E-01 | 3.24E-01 |
| Inflammation | 1 ± 0.6 | 1.8 ± 0.5**^a^** | 1.9 ± 0.6**^a^** | 7.89E-01 | **8.93E-03** |
| Ballooning | 0.1 ± 0.3 | 1.2 ± 0.4 | 1.4 ± 0.5 | 5.04E-01 | 3.99E-01 |
| NAS | 2.5 ± 0.8 | 4.7 ± 0.9**^a^** | 5 ± 1.2**^a^** | 9.40E-02 | **1.40E-06** |
| Fibrosis | 1.1 ± 0.9 | 1.3 ± 0.6 | 3.2 ± 0.4**^a,b^** | 8.40E-01 | **1.09E-05** |
| T2DM, n (%) | 3 (27) | 8 (67) | 5 (56) | - | 9.88E-02^#^ |
| Statin, n (%) | 5 (46) | 3 (25) | 5 (56) | - | 2.24E-01^#^ |
| Metformin, n (%) | 1 (9) | 4 (33) | 4 (44) | - | 5.00E-02^#^ |

*Data are mean ± SD and n (%). Statistical tests are two-way ANOVA with diabetes (T2DM) as covariate, and Pearson χ2 test #, as appropriate.* *Tukey HSD post hoc test was used to estimate the statistical significance among groups. A p-value <0.05 was considered significant. Lowercase letters indicate post hoc analysis significance: “a” means different from MASL, and “b” means different from MASH F0-F2. MASL = Metabolic Dysfunction-Associated Steatotic Liver, MASH = Metabolic Dysfunction-Associated Steatohepatitis, M = Male, F = Female, BMI = Body Mass Index, HOMA2-IR = Homeostasis Model Assessment 2 of Insulin Resistance, TG = Triglycerides, TC = Total Cholesterol, HDL-C = High-density lipoprotein cholesterol, LDL-C = Low-density lipoprotein cholesterol, AST = Aspartate aminotransaminase, ALT = Alanine aminotransaminase; NAS: NAFLD activity score.*

**Supplementary Table 6. Clinical characteristics of patients with MASL, MASH F0-2 or MASH F3-4 (targeted cohort).**

|  | **MASL n = 32** | **MASH F0-2 n = 32** | **MASH F3-4 n = 23** | **p-value** | | |
| --- | --- | --- | --- | --- | --- | --- |
|  |  |  |  | **T2DM Sex Disease** | | |
| Sex (M/F) | 23/9 | 19/13 | 12/11 | - | - | 2.93E-01^#^ |
| Age (years) | 53 ± 12 | 54 ± 11 | 60 ± 9 | **2.72E-02** | **1.61E-02** | 2.73E-01 |
| BMI (kg/m^2^) | 31 ± 4 | 32 ± 6 | 32 ± 4 | 1.05E-01 | **4.46E-03** | 3.58E-01 |
| Glucose (mmol/L) | 6.1 ± 2.3 | 6.8 ± 1.9 | 7.6 ± 2.1 | **4.77E-08** | 2.29E-01 | 5.77E-01 |
| Insulin (pmol/L) | 102 ± 46 | 146 ± 90 | 135 ± 67 | 1.86E-01 | 6.47E-01 | **3.60E-02** |
| HOMA2-IR | 1.9 ± 0.8 | 2.8 ± 1.6 | 2.7 ± 1.3 | **4.56E-02** | 7.89E-01 | **3.63E-02** |
| TG (mmol/L) | 1.6 ± 0.7 | 2.1 ± 1.7 | 1.6 ± 1.2 | 7.96E-01 | 7.96E-01 | 9.91E-02 |
| TC (mmol/L) | 4.4 ± 1.4 | 4.4 ± 0.9 | 4.3 ± 1.1 | 6.28E-01 | 2.28E-01 | 8.87E-01 |
| LDL-C (mmol/L) | 2.7 ± 1.3 | 2.6 ± 0.9 | 2.6 ± 0.8 | 4.66E-01 | 2.63E-01 | 8.11E-01 |
| HDL-C (mmol/l) | 1 ± 0.3 | 1 ± 0.2 | 1 ± 0.3 | 3.05E-01 | 4.29E-01 | 6.49E-01 |
| AST (U/L) | 35 ± 12 | 48 ± 28 | 54 ± 25**^a^** | 9.28E-01 | 8.55E-01 | **1.01E-03** |
| ALT (U/L) | 66 ± 38 | 65 ± 31 | 69 ± 32 | 1.84E-01 | **7.73E-03** | 3.09E-01 |
| Steatosis | 1.4 ± 0.7 | 2 ± 0.7**^a^** | 1.6 ± 0.7 | 5.11E-01 | 6.99E-01 | **9.71E-03** |
| Inflammation | 1.2 ± 0.6 | 1.7 ± 0.5**^a^** | 2 ± 0.6 | 1.03E-01 | 7.25E-01 | **9.89E-04** |
| Ballooning | 0.1 ± 0.3 | 1.2 ± 0.4 | 1.6 ± 0.5 | **2.11E-02** | 1.27E-01 | 2.17E-01 |
| NAS | 2.7 ± 0.9 | 4.8 ± 0.9**^a^** | 5 ± 1.1**^a^** | **7.51E-05** | 1.90E-01 | **3.82E-14** |
| Fibrosis | 0.9 ± 0.9 | 1.3 ± 0.6 | 3.2 ± 0.4**^a,b^** | **4.82E-03** | 2.38E-01 | **1.01E-14** |
| T2DM, n (%) | 9 (28) | 18 (56) | 19 (83) | - | - | **4.16E-04^#^** |
| Statin, n (%) | 9 (28) | 8 (25) | 11 (48) | - | - | 1.12E-01^#^ |
| Metformin, n (%) | 6 (19) | 10 (31) | 11 (48) |  |  | 1.21E-01^#^ |

*Data are mean ± SD and n (%). Statistical tests are three-way ANOVA with diabetes (T2DM) and sex as covariates, and Pearson χ2 test #, as appropriate. Tukey HSD post hoc test was used to estimate the statistical significance among groups.* *A p-value <0.05 was considered significant. Lowercase letters indicate post hoc analysis significance: “a” means different from MASL, and “b” means different from MASH F0-F2. MASL = Metabolic Dysfunction-Associated Steatotic Liver, MASH = Metabolic Dysfunction-Associated Steatohepatitis, M = Male, F = Female, BMI = Body Mass Index, HOMA2-IR = Homeostasis Model Assessment 2 of Insulin Resistance, TG = Triglycerides, TC = Total Cholesterol, HDL-C = High-density lipoprotein cholesterol, LDL-C = Low-density lipoprotein cholesterol, AST = Aspartate aminotransaminase, ALT = Alanine aminotransaminase; NAS: NAFLD activity score.*

**Supplementary Table 7. Differences in proteins in the untargeted cohort of healthy volunteers and patients with MASLD.**

| **Uniprot Accession** | **Gene** | **CTRL** | **MASLD** | **Log2FC** | **p (Disease)** | **p (T2DM)** | **FDR (disease)** |
| --- | --- | --- | --- | --- | --- | --- | --- |
| P01024 | C3 | 1.69E+00 ± 7.17E-01 | 5.57E+00 ± 1.29E+00 | 3.877 | 1.74E-10 | 2.68E-01 | 2.38E-08 |
| P02649 | APOE | -5.20E+00 ± 1.48E+00 | 1.97E+00 ± 3.06E+00 | 7.166 | 5.99E-08 | 4.86E-01 | 4.10E-06 |
| Q15942 | ZYX | -4.64E+00 ± 1.21E+00 | 1.13E-01 ± 2.31E+00 | 4.751 | 7.49E-07 | 2.28E-01 | 3.42E-05 |
| P01876 | IGHA1 | -4.60E-01 ± 3.68E-01 | 3.86E-01 ± 4.53E-01 | 0.846 | 1.03E-05 | 6.02E-01 | 3.52E-04 |
| O14791 | APOL1 | -6.26E+00 ± 9.48E-01 | -2.53E+00 ± 1.98E+00 | 3.727 | 1.13E-04 | 9.86E-01 | 2.78E-03 |
| P04217 | A1BG | -3.74E-01 ± 8.99E-01 | 1.05E+00 ± 8.99E-01 | 1.423 | 1.22E-04 | 1.12E-01 | 2.78E-03 |
| Q14624 | ITIH4 | 1.18E+00 ± 2.82E+00 | 3.82E+00 ± 1.27E+00 | 2.633 | 3.36E-04 | 8.84E-01 | 6.58E-03 |
| P01042 | KNG1 | -8.10E-01 ± 1.65E+00 | 1.94E+00 ± 1.83E+00 | 2.748 | 6.43E-04 | 9.13E-02 | 7.88E-03 |
| P06727 | APOA4 | 2.42E+00 ± 5.00E-01 | 4.64E+00 ± 1.70E+00 | 2.228 | 5.03E-04 | 9.43E-01 | 7.88E-03 |
| P68871 | HBB | 5.40E+00 ± 1.13E+00 | 6.47E+00 ± 6.26E-01 | 1.071 | 5.34E-04 | 1.84E-01 | 7.88E-03 |
| P01611 | IGKV1D-12 | -4.63E+00 ± 4.77E-01 | -5.58E+00 ± 7.18E-01 | -0.954 | 6.90E-04 | 1.53E-01 | 7.88E-03 |
| P01714 | IGLV3-19 | -4.63E+00 ± 4.77E-01 | -5.58E+00 ± 7.18E-01 | -0.954 | 6.90E-04 | 1.53E-01 | 7.88E-03 |
| P02671 | FGA | 6.99E+00 ± 9.30E-01 | 3.84E+00 ± 2.55E+00 | -3.146 | 1.01E-03 | 8.22E-01 | 1.06E-02 |
| Q04756 | HGFAC | -6.25E+00 ± 7.92E-01 | -3.06E+00 ± 1.72E+00 | 3.185 | 1.30E-03 | 5.84E-01 | 1.27E-02 |
| P69905 | HBA1 | 5.99E+00 ± 1.12E+00 | 6.93E+00 ± 6.13E-01 | 0.94 | 1.61E-03 | 1.35E-01 | 1.46E-02 |
| P00739 | HPR | -2.57E+00 ± 1.21E+00 | -4.21E+00 ± 1.31E+00 | -1.642 | 1.70E-03 | 2.23E-01 | 1.46E-02 |
| P01834 | IGKC | -6.76E-01 ± 2.17E-01 | -1.56E+00 ± 7.72E-01 | -0.883 | 1.94E-03 | 6.73E-01 | 1.56E-02 |
| Q08380 | LGALS3BP | -4.13E+00 ± 7.07E-01 | -1.77E+00 ± 2.03E+00 | 2.355 | 2.92E-03 | 4.50E-01 | 2.22E-02 |
| P04004 | VTN | 7.80E-01 ± 1.41E+00 | 2.13E+00 ± 1.10E+00 | 1.349 | 4.03E-03 | 3.01E-01 | 2.70E-02 |
| P02042 | HBD | -3.77E-01 ± 1.21E+00 | 7.36E-01 ± 9.17E-01 | 1.113 | 4.33E-03 | 1.60E-01 | 2.70E-02 |
| P02765 | AHSG | 4.93E+00 ± 4.49E-01 | 4.23E+00 ± 6.33E-01 | -0.698 | 4.18E-03 | 9.17E-01 | 2.70E-02 |
| A0A075B6S2 | IGKV2D-29 | -3.16E+00 ± 5.42E-01 | -4.11E+00 ± 8.80E-01 | -0.952 | 4.18E-03 | 3.91E-01 | 2.70E-02 |
| P37802 | TAGLN2 | -2.97E+00 ± 4.49E-01 | -3.86E+00 ± 8.36E-01 | -0.887 | 4.61E-03 | 6.53E-01 | 2.75E-02 |
| P00747 | PLG | -2.42E+00 ± 9.29E-01 | -1.48E+00 ± 8.13E-01 | 0.942 | 6.10E-03 | 7.84E-01 | 3.42E-02 |
| P05090 | APOD | -2.93E+00 ± 8.35E-01 | -3.83E+00 ± 8.10E-01 | -0.899 | 6.24E-03 | 4.04E-01 | 3.42E-02 |
| P04433 | IGKV3-11 | -3.41E-01 ± 4.76E-01 | -9.86E-01 ± 6.21E-01 | -0.645 | 7.18E-03 | 8.79E-01 | 3.78E-02 |
| P02768 | ALB | 4.16E+00 ± 8.22E-01 | 5.10E+00 ± 9.45E-01 | 0.942 | 8.29E-03 | 8.07E-02 | 4.21E-02 |
| P20930 | FLG | -2.37E+00 ± 7.42E-01 | -3.87E+00 ± 1.60E+00 | -1.498 | 9.99E-03 | 2.59E-01 | 4.89E-02 |
| P12259 | F5 | -2.39E+00 ± 1.43E+00 | -3.82E+00 ± 1.42E+00 | -1.431 | 1.13E-02 | 1.23E-01 | 5.36E-02 |
| P02656 | APOC3 | 5.91E+00 ± 7.45E-01 | 6.49E+00 ± 5.41E-01 | 0.582 | 1.30E-02 | 4.48E-01 | 5.76E-02 |
| A2NJV5 | IGKV2-29 | -4.80E+00 ± 1.43E+00 | -6.04E+00 ± 9.06E-01 | -1.241 | 1.28E-02 | 4.34E-01 | 5.76E-02 |
| Q05682 | CALD1 | -6.64E+00 ± 0.00E+00 | -3.95E+00 ± 1.72E+00 | 2.691 | 1.36E-02 | 5.72E-01 | 5.81E-02 |
| P01860 | IGHG3 | -6.33E+00 ± 7.09E-01 | -3.64E+00 ± 2.19E+00 | 2.684 | 1.40E-02 | 9.65E-01 | 5.81E-02 |
| P02753 | RBP4 | 3.73E+00 ± 2.89E-01 | 3.30E+00 ± 4.91E-01 | -0.437 | 1.46E-02 | 2.11E-01 | 5.88E-02 |
| A0A075B6P5 | IGKV2-28 | -1.20E-02 ± 4.94E-01 | -4.67E-01 ± 4.85E-01 | -0.455 | 1.58E-02 | 1.13E-01 | 6.18E-02 |
| P00738 | HP | 4.01E+00 ± 5.66E-01 | 1.79E+00 ± 2.68E+00 | -2.223 | 1.80E-02 | 1.95E-01 | 6.84E-02 |
| P02748 | C9 | -3.80E+00 ± 2.08E+00 | -2.55E+00 ± 1.03E+00 | 1.256 | 1.93E-02 | 2.30E-01 | 6.95E-02 |
| P01619 | IGKV3-20 | 2.69E-01 ± 4.28E-01 | -1.51E-01 ± 4.54E-01 | -0.420 | 1.91E-02 | 9.20E-01 | 6.95E-02 |
| P02452 | COL1A1 | -6.64E+00 ± 1.92E-15 | -6.03E+00 ± 6.85E-01 | 0.617 | 2.22E-02 | 7.20E-02 | 7.79E-02 |
| P01009 | SERPINA1 | 8.53E-01 ± 7.83E-01 | 2.01E+00 ± 1.40E+00 | 1.157 | 2.30E-02 | 2.70E-01 | 7.87E-02 |
| P02647 | APOA1 | 6.73E+00 ± 4.58E-01 | 7.40E+00 ± 8.13E-01 | 0.666 | 2.62E-02 | 8.14E-01 | 8.56E-02 |
| O95445 | APOM | 3.47E+00 ± 4.30E-01 | 3.08E+00 ± 4.55E-01 | -0.397 | 2.62E-02 | 6.40E-01 | 8.56E-02 |
| A0A0C4DH25 | IGKV3D-20 | -4.77E+00 ± 6.43E-01 | -3.58E+00 ± 1.52E+00 | 1.188 | 3.02E-02 | 6.80E-01 | 9.05E-02 |
| P0DOX5 |  | 7.70E-01 ± 5.33E-01 | 2.01E-01 ± 6.93E-01 | -0.569 | 2.87E-02 | 3.17E-01 | 9.05E-02 |
| P0DOX7 |  | -4.85E-01 ± 3.06E-01 | -1.12E+00 ± 8.22E-01 | -0.635 | 3.08E-02 | 4.45E-01 | 9.05E-02 |
| P01594 | IGKV1-33 | -2.05E+00 ± 4.23E-01 | -2.79E+00 ± 9.62E-01 | -0.746 | 3.16E-02 | 5.36E-01 | 9.05E-02 |
| P68363 | TUBA1B | -2.05E+00 ± 4.23E-01 | -2.79E+00 ± 9.62E-01 | -0.746 | 3.16E-02 | 5.36E-01 | 9.05E-02 |
| P01031 | C5 | -2.64E+00 ± 1.74E+00 | -4.49E+00 ± 1.75E+00 | -1.850 | 3.17E-02 | 6.98E-01 | 9.05E-02 |
| P0DOX8 |  | 1.56E+00 ± 2.58E-01 | 1.11E+00 ± 5.90E-01 | -0.448 | 3.58E-02 | 7.32E-01 | 1.00E-01 |
| P61626 | LYZ | 1.89E+00 ± 2.27E-01 | 1.42E+00 ± 6.20E-01 | -0.462 | 3.73E-02 | 6.25E-01 | 1.00E-01 |
| P00488 | F13A1 | 1.02E+00 ± 7.13E-01 | 3.48E-02 ± 1.20E+00 | -0.981 | 3.68E-02 | 4.75E-01 | 1.00E-01 |
| P02655 | APOC2 | 4.21E+00 ± 9.72E-01 | 4.89E+00 ± 8.08E-01 | 0.677 | 3.98E-02 | 2.93E-01 | 1.01E-01 |
| P01615 | IGKV2D-28 | -1.52E+00 ± 9.13E-01 | -2.67E+00 ± 1.54E+00 | -1.144 | 3.95E-02 | 1.17E-01 | 1.01E-01 |
| P0DJI9 | SAA2 | -1.52E+00 ± 9.13E-01 | -2.67E+00 ± 1.54E+00 | -1.144 | 3.95E-02 | 1.17E-01 | 1.01E-01 |
| P19652 | ORM2 | -1.38E+00 ± 7.31E-01 | -2.38E+00 ± 1.34E+00 | -1.003 | 4.18E-02 | 8.46E-01 | 1.04E-01 |
| Q9Y613 | FHOD1 | -1.06E+00 ± 3.19E+00 | -3.46E+00 ± 2.40E+00 | -2.401 | 4.31E-02 | 2.22E-01 | 1.06E-01 |
| P07357 | C8A | -5.05E+00 ± 9.54E-01 | -5.84E+00 ± 1.04E+00 | -0.790 | 5.29E-02 | 9.86E-02 | 1.27E-01 |
| P0DJI8 | SAA1 | 3.00E-01 ± 1.10E+00 | 1.25E+00 ± 1.33E+00 | 0.955 | 5.97E-02 | 9.02E-01 | 1.41E-01 |
| Q96PD5 | PGLYRP2 | 1.61E-01 ± 5.57E-01 | -4.19E-01 ± 8.51E-01 | -0.579 | 6.06E-02 | 2.15E-01 | 1.41E-01 |
| O76011 | KRT34 | -6.00E+00 ± 7.83E-01 | -4.82E+00 ± 1.23E+00 | 1.179 | 7.10E-02 | 1.32E-01 | 1.55E-01 |
| P05452 | CLEC3B | 1.27E+00 ± 3.47E-01 | 9.49E-01 ± 4.71E-01 | -0.317 | 7.17E-02 | 9.55E-01 | 1.55E-01 |
| P02533 | KRT14 | -5.26E-01 ± 3.18E-01 | -9.63E-01 ± 6.92E-01 | -0.437 | 7.13E-02 | 1.46E-01 | 1.55E-01 |
| Q16627 | CCL14 | -4.86E+00 ± 9.19E-01 | -5.51E+00 ± 8.65E-01 | -0.649 | 7.01E-02 | 7.84E-02 | 1.55E-01 |
| P02760 | AMBP | -4.60E+00 ± 1.02E+00 | -5.41E+00 ± 1.02E+00 | -0.809 | 7.26E-02 | 6.64E-01 | 1.55E-01 |
| Q9Y251 | HPSE | -5.45E+00 ± 3.38E-01 | -4.33E+00 ± 1.06E+00 | 1.116 | 7.44E-02 | 7.97E-02 | 1.57E-01 |
| P08697 | SERPINF2 | -2.87E+00 ± 7.10E-01 | -1.90E+00 ± 1.52E+00 | 0.973 | 7.82E-02 | 6.15E-02 | 1.58E-01 |
| Q92954 | PRG4 | 4.44E-01 ± 6.89E-01 | 9.01E-01 ± 6.53E-01 | 0.457 | 7.69E-02 | 5.71E-01 | 1.58E-01 |
| P02766 | TTR | 5.78E+00 ± 1.81E-01 | 6.19E+00 ± 6.77E-01 | 0.414 | 7.81E-02 | 2.39E-01 | 1.58E-01 |
| P04196 | HRG | -4.25E+00 ± 6.88E-01 | -2.92E+00 ± 2.05E+00 | 1.337 | 8.43E-02 | 7.44E-01 | 1.67E-01 |
| P01308 | INS | -5.29E+00 ± 1.26E+00 | -4.08E+00 ± 1.65E+00 | 1.210 | 8.61E-02 | 4.44E-01 | 1.68E-01 |
| P59665 | DEFA1 | -3.32E+00 ± 8.76E-01 | -4.30E+00 ± 1.55E+00 | -0.975 | 8.96E-02 | 7.91E-01 | 1.73E-01 |
| P02749 | APOH | 1.13E+00 ± 6.86E-01 | 1.59E+00 ± 7.28E-01 | 0.461 | 1.00E-01 | 5.37E-01 | 1.91E-01 |
| P04430 | IGKV1-16 | -3.30E+00 ± 1.24E+00 | -4.25E+00 ± 1.42E+00 | -0.950 | 1.05E-01 | 8.04E-01 | 1.96E-01 |
| P02751 | FN1 | -1.29E+00 ± 5.48E-01 | -1.79E+00 ± 8.45E-01 | -0.493 | 1.07E-01 | 2.90E-01 | 1.99E-01 |
| Q9Y490 | TLN1 | -2.12E+00 ± 7.40E-01 | -2.81E+00 ± 1.22E+00 | -0.689 | 1.13E-01 | 8.60E-02 | 2.07E-01 |
| P01597 | IGKV1-39 | -5.43E+00 ± 1.04E+00 | -4.83E+00 ± 9.81E-01 | 0.598 | 1.25E-01 | 3.12E-01 | 2.10E-01 |
| P0CG47 | UBB | -5.70E+00 ± 1.10E+00 | -5.20E+00 ± 5.60E-01 | 0.502 | 1.21E-01 | 4.31E-01 | 2.10E-01 |
| P0CG48 | UBC | -5.70E+00 ± 1.10E+00 | -5.20E+00 ± 5.60E-01 | 0.502 | 1.21E-01 | 4.31E-01 | 2.10E-01 |
| P16930 | FAH | -5.70E+00 ± 1.10E+00 | -5.20E+00 ± 5.60E-01 | 0.502 | 1.21E-01 | 4.31E-01 | 2.10E-01 |
| P62987 | UBA52 | -5.70E+00 ± 1.10E+00 | -5.20E+00 ± 5.60E-01 | 0.502 | 1.21E-01 | 4.31E-01 | 2.10E-01 |
| P31151 | S100A7 | -2.88E+00 ± 2.69E-01 | -2.71E+00 ± 2.85E-01 | 0.178 | 1.26E-01 | 4.35E-01 | 2.10E-01 |
| P08571 | CD14 | -5.28E+00 ± 1.38E+00 | -6.05E+00 ± 6.56E-01 | -0.769 | 1.23E-01 | 5.69E-01 | 2.10E-01 |
| P30043 | BLVRB | -4.34E+00 ± 7.24E-01 | -4.86E+00 ± 9.80E-01 | -0.521 | 1.32E-01 | 2.24E-02 | 2.18E-01 |
| P0DOX2 |  | -4.30E+00 ± 5.10E-01 | -3.91E+00 ± 7.06E-01 | 0.392 | 1.59E-01 | 5.08E-01 | 2.57E-01 |
| P02654 | APOC1 | 4.58E+00 ± 4.82E-01 | 4.30E+00 ± 5.46E-01 | -0.285 | 1.58E-01 | 1.31E-01 | 2.57E-01 |
| P01008 | SERPINC1 | -1.07E+00 ± 4.65E-01 | -1.41E+00 ± 6.68E-01 | -0.338 | 1.63E-01 | 2.86E-01 | 2.60E-01 |
| P01023 | A2M | -3.93E+00 ± 9.41E-01 | -3.22E+00 ± 1.40E+00 | 0.710 | 1.69E-01 | 8.44E-01 | 2.63E-01 |
| A0A0B4J1X5 | IGHV3-74 | -4.75E+00 ± 6.41E-01 | -5.25E+00 ± 8.58E-01 | -0.492 | 1.67E-01 | 4.28E-01 | 2.63E-01 |
| O60885 | BRD4 | -3.83E+00 ± 2.25E-01 | -4.09E+00 ± 5.26E-01 | -0.261 | 1.91E-01 | 4.30E-01 | 2.90E-01 |
| P01591 | JCHAIN | -4.28E+00 ± 6.37E-01 | -3.66E+00 ± 1.48E+00 | 0.619 | 2.34E-01 | 3.19E-01 | 3.52E-01 |
| P05019 | IGF1 | -5.67E-02 ± 4.26E-01 | -2.79E-01 ± 5.38E-01 | -0.222 | 2.66E-01 | 5.74E-01 | 3.96E-01 |
| P36980 | CFHR2 | -2.61E+00 ± 8.30E-01 | -3.06E+00 ± 1.11E+00 | -0.448 | 2.69E-01 | 2.92E-01 | 3.96E-01 |
| P01717 | IGLV3-25 | -4.74E+00 ± 1.43E+00 | -4.06E+00 ± 1.31E+00 | 0.681 | 2.83E-01 | 1.69E-01 | 4.13E-01 |
| P49747 | COMP | -3.48E+00 ± 1.88E+00 | -4.02E+00 ± 1.08E+00 | -0.540 | 3.10E-01 | 6.55E-01 | 4.47E-01 |
| P18065 | IGFBP2 | -3.43E+00 ± 3.47E-01 | -3.07E+00 ± 1.07E+00 | 0.358 | 3.14E-01 | 5.65E-02 | 4.48E-01 |
| P14625 | HSP90B1 | -5.85E+00 ± 5.85E-01 | -5.50E+00 ± 8.47E-01 | 0.346 | 3.34E-01 | 4.87E-01 | 4.67E-01 |
| P09871 | C1S | -4.25E+00 ± 1.14E+00 | -4.64E+00 ± 1.10E+00 | -0.390 | 3.34E-01 | 3.57E-02 | 4.67E-01 |
| P16035 | TIMP2 | -5.16E+00 ± 8.32E-01 | -5.51E+00 ± 9.68E-01 | -0.351 | 3.40E-01 | 5.05E-01 | 4.70E-01 |
| P02750 | LRG1 | 1.05E+00 ± 6.45E-01 | 7.96E-01 ± 7.28E-01 | -0.258 | 3.46E-01 | 4.93E-01 | 4.74E-01 |
| P02776 | PF4 | 3.25E+00 ± 3.87E-01 | 2.85E+00 ± 1.27E+00 | -0.4 | 3.65E-01 | 6.77E-01 | 4.96E-01 |
| P02775 | PPBP | 4.72E+00 ± 1.70E-01 | 4.33E+00 ± 1.29E+00 | -0.389 | 3.85E-01 | 9.31E-01 | 5.17E-01 |
| P13647 | KRT5 | -4.46E+00 ± 8.56E-01 | -4.02E+00 ± 1.52E+00 | 0.446 | 4.04E-01 | 2.03E-01 | 5.32E-01 |
| P14923 | JUP | -6.64E+00 ± 1.92E-15 | -6.64E+00 ± 9.73E-16 | 0.000 | 4.01E-01 | 1.00E+00 | 5.32E-01 |
| P05155 | SERPING1 | -5.07E+00 ± 9.28E-01 | -4.80E+00 ± 7.86E-01 | 0.267 | 4.09E-01 | 1.18E-01 | 5.34E-01 |
| O60942 | RNGTT | -2.75E+00 ± 9.97E-01 | -2.53E+00 ± 6.06E-01 | 0.226 | 4.13E-01 | 8.54E-01 | 5.34E-01 |
| A0A075B6S5 | IGKV1-27 | -1.77E+00 ± 2.27E+00 | -2.41E+00 ± 2.02E+00 | -0.642 | 4.32E-01 | 1.82E-01 | 5.53E-01 |
| P10720 | PF4V1 | -2.16E+00 ± 5.36E-01 | -2.44E+00 ± 1.08E+00 | -0.274 | 4.55E-01 | 6.46E-02 | 5.77E-01 |
| Q86YW5 | TREML1 | -1.51E+00 ± 4.19E-01 | -1.86E+00 ± 1.44E+00 | -0.355 | 4.62E-01 | 8.60E-02 | 5.80E-01 |
| P14209 | CD99 | -5.48E+00 ± 2.85E-01 | -5.69E+00 ± 7.83E-01 | -0.209 | 4.81E-01 | 7.41E-02 | 5.99E-01 |
| Q16610 | ECM1 | 1.01E-01 ± 5.31E-01 | -1.08E-01 ± 9.52E-01 | -0.209 | 5.40E-01 | 8.45E-01 | 6.66E-01 |
| P04264 | KRT1 | -2.32E+00 ± 3.65E+00 | -3.16E+00 ± 2.56E+00 | -0.838 | 5.70E-01 | 6.90E-01 | 6.97E-01 |
| P00734 | F2 | 4.71E+00 ± 2.94E-01 | 4.39E+00 ± 1.78E+00 | -0.328 | 5.90E-01 | 4.01E-01 | 7.16E-01 |
| P35527 | KRT9 | -3.86E+00 ± 2.53E+00 | -3.37E+00 ± 2.33E+00 | 0.489 | 6.73E-01 | 2.21E-01 | 7.88E-01 |
| P17936 | IGFBP3 | -3.62E+00 ± 1.39E+00 | -3.30E+00 ± 1.46E+00 | 0.320 | 6.61E-01 | 6.40E-01 | 7.88E-01 |
| P25311 | AZGP1 | 1.79E-01 ± 5.22E-01 | 9.02E-02 ± 5.58E-01 | -0.089 | 6.73E-01 | 3.98E-01 | 7.88E-01 |
| Q15323 | KRT31 | -5.45E+00 ± 1.06E+00 | -5.61E+00 ± 9.61E-01 | -0.165 | 6.69E-01 | 9.44E-01 | 7.88E-01 |
| P01861 | IGHG4 | -3.35E+00 ± 1.37E+00 | -3.58E+00 ± 1.38E+00 | -0.223 | 6.78E-01 | 8.91E-01 | 7.88E-01 |
| P13645 | KRT10 | -4.15E+00 ± 3.70E+00 | -3.71E+00 ± 2.51E+00 | 0.441 | 7.08E-01 | 1.02E-01 | 8.15E-01 |
| P02790 | HPX | -3.95E+00 ± 1.55E+00 | -4.15E+00 ± 1.44E+00 | -0.201 | 7.16E-01 | 2.07E-01 | 8.18E-01 |
| P00746 | CFD | 2.60E-01 ± 5.22E-01 | 3.18E-01 ± 5.32E-01 | 0.059 | 7.65E-01 | 8.26E-02 | 8.66E-01 |
| P01780 | IGHV3-7 | -5.01E+00 ± 6.24E-01 | -4.90E+00 ± 9.67E-01 | 0.107 | 7.73E-01 | 2.33E-01 | 8.68E-01 |
| P01019 | AGT | -1.84E+00 ± 8.89E-01 | -1.69E+00 ± 1.65E+00 | 0.158 | 7.87E-01 | 7.73E-01 | 8.70E-01 |
| P20929 | NEB | -6.07E+00 ± 5.35E-01 | -6.14E+00 ± 5.56E-01 | -0.067 | 7.86E-01 | 9.75E-01 | 8.70E-01 |
| P61769 | B2M | 8.03E-01 ± 5.48E-01 | 7.18E-01 ± 1.02E+00 | -0.085 | 8.05E-01 | 4.48E-02 | 8.83E-01 |
| P02787 | TF | 2.87E+00 ± 4.48E-01 | 2.91E+00 ± 6.48E-01 | 0.043 | 8.50E-01 | 9.35E-02 | 9.12E-01 |
| P01601 | IGKV1D-16 | -3.10E+00 ± 7.08E-01 | -3.15E+00 ± 7.64E-01 | -0.054 | 8.52E-01 | 5.35E-01 | 9.12E-01 |
| P59666 | DEFA3 | -3.10E+00 ± 7.08E-01 | -3.15E+00 ± 7.64E-01 | -0.054 | 8.52E-01 | 5.35E-01 | 9.12E-01 |
| Q13790 | APOF | -1.92E+00 ± 8.38E-01 | -1.83E+00 ± 1.95E+00 | 0.093 | 8.91E-01 | 3.53E-01 | 9.42E-01 |
| P02763 | ORM1 | 3.24E-01 ± 9.34E-01 | 3.77E-01 ± 1.37E+00 | 0.054 | 9.12E-01 | 1.59E-01 | 9.42E-01 |
| P02743 | APCS | -5.43E+00 ± 7.20E-01 | -5.40E+00 ± 5.91E-01 | 0.028 | 9.15E-01 | 6.81E-01 | 9.42E-01 |
| A0A0B4J1U3 | IGLV1-36 | -6.32E+00 ± 6.18E-01 | -6.35E+00 ± 5.76E-01 | -0.031 | 8.97E-01 | 8.92E-01 | 9.42E-01 |
| Q03591 | CFHR1 | -4.10E+00 ± 9.69E-01 | -4.16E+00 ± 1.42E+00 | -0.060 | 9.08E-01 | 5.01E-01 | 9.42E-01 |
| P02652 | APOA2 | 7.21E+00 ± 4.97E-01 | 7.20E+00 ± 4.07E-01 | -0.007 | 9.63E-01 | 3.55E-01 | 9.82E-01 |
| O95810 | CAVIN2 | -3.20E+00 ± 1.41E+00 | -3.21E+00 ± 8.54E-01 | -0.016 | 9.68E-01 | 3.51E-01 | 9.82E-01 |
| P02774 | GC | 2.60E-02 ± 6.81E-01 | 1.64E-02 ± 9.15E-01 | -0.01 | 9.76E-01 | 8.89E-02 | 9.83E-01 |
| P07988 | SFTPB | -4.60E+00 ± 1.11E+00 | -4.60E+00 ± 1.29E+00 | -0.007 | 9.88E-01 | 7.46E-01 | 9.88E-01 |

*Data are represented (log 2 transformed) as mean ± standard deviation. Statistical significance was calculated using a two-way ANOVA controlling for presence of type 2 diabetes mellitus (T2DM)* *as covariate. A p-value <0.05 was considered significant. The adjusted p-value was calculated using the false discovery rate (FDR) as described in the methods.*

**Supplementary Table 8. Differences in proteins in the untargeted cohort of MASLD patients clustered against MASL, MASH F0-2, MASH F3-4.**

| **Uniprot Accession** | **Gene** | **MASL** | **MASH F0-2** | **MASH F3-4** | **p (Disease)** | **p (T2DM)** | **FDR (Disease)** |
| --- | --- | --- | --- | --- | --- | --- | --- |
| A0A075B6P5 | IGKV2-28 | -0.38 ± 0.55 | -0.62 ± 0.33 | -0.38 ± 0.58 | 3.59E-01 | 4.03E-02 | 8.88E-01 |
| A0A075B6S2 | IGKV2D-29 | -4.12 ± 1.15 | -3.99 ± 0.69 | -4.29 ± 0.78 | 7.60E-01 | 3.88E-01 | 9.99E-01 |
| A0A075B6S5 | IGKV1-27 | -3.16 ± 2.53 | -1.79 ± 1.81 | -2.32 ± 1.4 | 2.77E-01 | 3.87E-01 | 8.88E-01 |
| A0A087WSY4 | IGHV4-30-2 | -6.24 ± 0.55 | -6.2 ± 0.73 | -6.24 ± 0.55 | 9.89E-01 | 3.12E-01 | 9.99E-01 |
| A0A0B4J1U3 | IGLV1-36 | -6.32 ± 0.62 | -6.34 ± 0.48 | -6.39 ± 0.71 | 9.71E-01 | 8.38E-01 | 9.99E-01 |
| A0A0B4J1X5 | IGHV3-74 | -5.67 ± 0.95 | -4.93 ± 0.77 | -5.2 ± 0.73 | 1.12E-01 | 1.01E-01 | 8.75E-01 |
| A0A0C4DH25 | IGKV3D-20 | -4.05 ± 1.49 | -3.03 ± 1.72 | -3.73 ± 1.14 | 2.77E-01 | 9.19E-01 | 8.88E-01 |
| A0A0C4DH41 | IGHV4-61 | -6.24 ± 0.55 | -6.2 ± 0.73 | -6.24 ± 0.55 | 9.89E-01 | 3.12E-01 | 9.99E-01 |
| A2NJV5 | IGKV2-29 | -5.78 ± 1.19 | -6.48 ± 0.41 | -5.83 ± 0.99 | 3.79E-01 | 4.89E-01 | 8.93E-01 |
| O00151 | PDLIM1 | -3.95 ± 0.83 | -4.77 ± 1.26 | -4.8 ± 1.95 | 4.94E-01 | 6.89E-01 | 8.98E-01 |
| O14791 | APOL1 | -2.93 ± 1.81 | -1.72 ± 1.68 | -3.11 ± 2.33 | 2.44E-01 | 7.72E-01 | 8.88E-01 |
| O60885 | BRD4 | -4.16 ± 0.72 | -3.9 ± 0.23 | -4.21 ± 0.52 | 5.05E-01 | 6.11E-01 | 8.98E-01 |
| O60942 | RNGTT | -2.48 ± 0.53 | -2.44 ± 0.8 | -2.72 ± 0.32 | 5.89E-01 | 8.94E-01 | 9.67E-01 |
| O76011 | KRT34 | -5.23 ± 0.82 | -4.16 ± 1.36 | -5.62 ± 0.58**^b^** | **3.26E-02** | 2.62E-01 | 6.75E-01 |
| O95445 | APOM | 3.2 ± 0.58 | 3.08 ± 0.4 | 2.93 ± 0.34 | 4.33E-01 | 3.51E-01 | 8.98E-01 |
| O95810 | CAVIN2 | -3.36 ± 0.94 | -3.11 ± 0.92 | -3.17 ± 0.71 | 7.78E-01 | 3.98E-01 | 9.99E-01 |
| P00488 | F13A1 | 0.55 ± 0.82 | -0.7 ± 1.43 | 0.34 ± 0.91 | 5.06E-02 | 9.54E-01 | 6.75E-01 |
| P00734 | F2 | 4.63 ± 0.4 | 4.19 ± 2.84 | 4.38 ± 0.72 | 8.62E-01 | 5.57E-01 | 9.99E-01 |
| P00738 | HP | 1.96 ± 2.69 | 1.71 ± 2.71 | 1.68 ± 2.95 | 9.68E-01 | 1.90E-01 | 9.99E-01 |
| P00739 | HPR | -4.29 ± 1.56 | -3.92 ± 1.23 | -4.51 ± 1.17 | 5.94E-01 | 2.42E-01 | 9.67E-01 |
| P00746 | CFD | 0.18 ± 0.42 | 0.45 ± 0.62 | 0.32 ± 0.56 | 4.90E-01 | 1.59E-01 | 8.98E-01 |
| P00747 | PLG | -1.58 ± 0.61 | -1.28 ± 0.86 | -1.61 ± 0.98 | 6.09E-01 | 6.61E-01 | 9.71E-01 |
| P01008 | SERPINC1 | -1.26 ± 0.54 | -1.4 ± 0.89 | -1.61 ± 0.45 | 5.17E-01 | 4.84E-01 | 8.98E-01 |
| P01009 | SERPINA1 | 2 ± 1.45 | 2.01 ± 1.03 | 2.03 ± 1.87 | 9.99E-01 | 2.84E-01 | 9.99E-01 |
| P01019 | AGT | -1.79 ± 1.44 | -1.16 ± 1.32 | -2.26 ± 2.18 | 3.27E-01 | 8.55E-01 | 8.88E-01 |
| P01023 | A2M | -3.25 ± 1.14 | -3.62 ± 1.53 | -2.7 ± 1.48 | 3.71E-01 | 8.78E-01 | 8.88E-01 |
| P01024 | C3 | 5.14 ± 1.44 | 5.93 ± 1.28 | 5.59 ± 1.07 | 3.28E-01 | 9.60E-02 | 8.88E-01 |
| P01031 | C5 | -4.19 ± 1.88 | -4.97 ± 1.56 | -4.42 ± 1.93 | 6.95E-01 | 5.66E-01 | 9.95E-01 |
| P01042 | KNG1 | 2.04 ± 1.76 | 1.93 ± 2.3 | 1.83 ± 1.34 | 9.72E-01 | 1.02E-01 | 9.99E-01 |
| P01308 | INS | -4.3 ± 1.85 | -4 ± 1.69 | -4.04 ± 1.67 | 9.39E-01 | 3.98E-01 | 9.99E-01 |
| P01591 | JCHAIN | -3.76 ± 0.94 | -3.54 ± 1.99 | -3.7 ± 1.48 | 9.44E-01 | 3.98E-01 | 9.99E-01 |
| P01594 | IGKV1-33 | -2.94 ± 0.97 | -2.85 ± 1.08 | -2.53 ± 0.84 | 6.41E-01 | 7.30E-01 | 9.71E-01 |
| P01597 | IGKV1-39 | -4.94 ± 0.92 | -5.03 ± 0.85 | -4.37 ± 1.23 | 3.64E-01 | 4.96E-01 | 8.88E-01 |
| P01601 | IGKV1D-16 | -3.27 ± 0.82 | -2.92 ± 0.5 | -3.31 ± 0.98 | 4.48E-01 | 6.89E-01 | 8.98E-01 |
| P01611 | IGKV1D-12 | -5.77 ± 0.66 | -5.26 ± 0.48 | -5.67 ± 0.94 | 3.08E-01 | 2.92E-01 | 8.88E-01 |
| P01614 | IGKV2D-40 | -2.15 ± 1.34 | -2.1 ± 1.82 | -1.61 ± 1.22 | 7.21E-01 | 6.58E-01 | 9.99E-01 |
| P01615 | IGKV2D-28 | -1.9 ± 0.46 | -3.16 ± 1.86 | -2.91 ± 1.72 | 1.84E-01 | 3.95E-01 | 8.88E-01 |
| P01619 | IGKV3-20 | -0.18 ± 0.65 | -0.14 ± 0.35 | -0.12 ± 0.3 | 9.52E-01 | 8.26E-01 | 9.99E-01 |
| P01699 | IGLV1-44 | -4.49 ± 3.41 | -5.02 ± 0.86 | -5.62 ± 1.42 | 6.96E-01 | 9.94E-01 | 9.95E-01 |
| P01714 | IGLV3-19 | -5.77 ± 0.66 | -5.26 ± 0.48 | -5.67 ± 0.94 | 3.08E-01 | 2.92E-01 | 8.88E-01 |
| P01780 | IGHV3-7 | -5.26 ± 0.92 | -4.8 ± 0.94 | -4.79 ± 1.11 | 6.64E-01 | 3.97E-01 | 9.95E-01 |
| P01782 | IGHV3-9 | -4.72 ± 0.94 | -4.88 ± 1.95 | -5.08 ± 1.46 | 8.47E-01 | 2.65E-02 | 9.99E-01 |
| P01824 | IGHV4-39 | -6.24 ± 0.55 | -6.2 ± 0.73 | -6.24 ± 0.55 | 9.89E-01 | 3.12E-01 | 9.99E-01 |
| P01825 | IGHV4-59 | -6.24 ± 0.55 | -6.2 ± 0.73 | -6.24 ± 0.55 | 9.89E-01 | 3.12E-01 | 9.99E-01 |
| P01834 | IGKC | -1.34 ± 0.64 | -1.74 ± 0.98 | -1.59 ± 0.61 | 5.00E-01 | 9.65E-01 | 8.98E-01 |
| P01860 | IGHG3 | -4.3 ± 1.62 | -2.7 ± 2.58 | -4.24 ± 1.92 | 2.27E-01 | 5.36E-01 | 8.88E-01 |
| P01861 | IGHG4 | -3.71 ± 1.7 | -3.46 ± 1.52 | -3.57 ± 0.77 | 9.23E-01 | 9.86E-01 | 9.99E-01 |
| P01876 | IGHA1 | 0.3 ± 0.49 | 0.61 ± 0.37 | 0.18 ± 0.41 | 7.49E-02 | 8.02E-01 | 8.75E-01 |
| P02042 | HBD | 1.19 ± 0.66 | 0.25 ± 1.02**^a^** | 0.83 ± 0.8 | **4.01E-02** | 3.88E-01 | 6.75E-01 |
| P02533 | KRT14 | -0.65 ± 0.54 | -0.87 ± 0.65 | -1.46 ± 0.7**^a^** | **2.38E-02** | 4.53E-01 | 6.75E-01 |
| P02647 | APOA1 | 7.17 ± 0.52 | 7.85 ± 0.89 | 7.09 ± 0.81 | 5.28E-02 | 4.53E-01 | 6.75E-01 |
| P02649 | APOE | 0.85 ± 3.69 | 2.88 ± 2.39 | 2.11 ± 2.91 | 2.78E-01 | 1.92E-01 | 8.88E-01 |
| P02652 | APOA2 | 7.03 ± 0.46 | 7.42 ± 0.21 | 7.12 ± 0.44 | 5.04E-02 | 7.06E-01 | 6.75E-01 |
| P02654 | APOC1 | 4.22 ± 0.3 | 4.5 ± 0.58 | 4.11 ± 0.69 | 2.30E-01 | 1.65E-01 | 8.88E-01 |
| P02655 | APOC2 | 4.72 ± 0.83 | 5.22 ± 0.64 | 4.65 ± 0.9 | 1.92E-01 | 3.93E-01 | 8.88E-01 |
| P02656 | APOC3 | 6.43 ± 0.61 | 6.64 ± 0.44 | 6.38 ± 0.59 | 5.22E-01 | 4.90E-01 | 8.98E-01 |
| P02671 | FGA | 4.32 ± 2.53 | 3.18 ± 3.13 | 4.15 ± 1.64 | 5.37E-01 | 6.07E-01 | 9.13E-01 |
| P02743 | APCS | -5.57 ± 0.66 | -5.24 ± 0.68 | -5.4 ± 0.38 | 4.84E-01 | 9.82E-01 | 8.98E-01 |
| P02748 | C9 | -2.32 ± 0.64 | -2.35 ± 0.85 | -3.07 ± 1.47 | 2.09E-01 | 5.57E-02 | 8.88E-01 |
| P02749 | APOH | 1.53 ± 0.74 | 1.64 ± 0.87 | 1.6 ± 0.58 | 9.45E-01 | 4.46E-01 | 9.99E-01 |
| P02750 | LRG1 | 0.9 ± 0.79 | 0.77 ± 0.66 | 0.69 ± 0.8 | 8.23E-01 | 3.51E-01 | 9.99E-01 |
| P02751 | FN1 | -1.72 ± 0.77 | -1.63 ± 0.82 | -2.09 ± 0.97 | 4.41E-01 | 2.30E-01 | 8.98E-01 |
| P02753 | RBP4 | 3.41 ± 0.61 | 3.29 ± 0.27 | 3.16 ± 0.56 | 5.16E-01 | 1.01E-01 | 8.98E-01 |
| P02760 | AMBP | -5.58 ± 1.04 | -5.3 ± 0.97 | -5.41 ± 1.17 | 8.45E-01 | 7.75E-01 | 9.99E-01 |
| P02763 | ORM1 | 0.62 ± 1.75 | 0.53 ± 1.23 | -0.12 ± 0.97 | 4.38E-01 | 2.58E-01 | 8.98E-01 |
| P02765 | AHSG | 4.54 ± 0.69 | 4.05 ± 0.59 | 4.11 ± 0.54 | 1.47E-01 | 3.61E-01 | 8.88E-01 |
| P02766 | TTR | 6.21 ± 0.55 | 6.14 ± 0.49 | 6.24 ± 1.03 | 9.35E-01 | 2.87E-01 | 9.99E-01 |
| P02768 | ALB | 4.98 ± 1.01 | 5.48 ± 1.07 | 4.75 ± 0.49 | 1.60E-01 | 3.35E-02 | 8.88E-01 |
| P02774 | GC | -0.07 ± 1.06 | 0.28 ± 0.95 | -0.23 ± 0.64 | 3.85E-01 | 5.49E-02 | 8.94E-01 |
| P02775 | PPBP | 4.31 ± 1.4 | 4.28 ± 1.65 | 4.43 ± 0.56 | 9.69E-01 | 9.61E-01 | 9.99E-01 |
| P02776 | PF4 | 2.82 ± 1.32 | 2.71 ± 1.63 | 3.05 ± 0.59 | 8.43E-01 | 7.19E-01 | 9.99E-01 |
| P02787 | TF | 2.96 ± 0.76 | 3.03 ± 0.68 | 2.7 ± 0.43 | 5.03E-01 | 1.19E-01 | 8.98E-01 |
| P02790 | HPX | -4.67 ± 1.09 | -3.49 ± 0.9 | -4.4 ± 2.09 | 1.24E-01 | 4.15E-01 | 8.88E-01 |
| P04004 | VTN | 2.17 ± 0.51 | 2.18 ± 1.42 | 2.01 ± 1.25 | 9.35E-01 | 2.75E-01 | 9.99E-01 |
| P04114 | APOB | -2.53 ± 2.13 | 0.8 ± 3.05 | -1.53 ± 3.49 | 8.97E-02 | 3.03E-01 | 8.75E-01 |
| P04196 | HRG | -3.69 ± 0.97 | -1.77 ± 1.35 | -3.19 ± 2.99 | 9.78E-02 | 1.51E-01 | 8.75E-01 |
| P04217 | A1BG | 0.83 ± 1.07 | 1.07 ± 0.88 | 1.28 ± 0.71 | 5.15E-01 | 3.46E-02 | 8.98E-01 |
| P04264 | KRT1 | -3.15 ± 2.3 | -3.11 ± 3.08 | -3.23 ± 2.5 | 9.96E-01 | 6.67E-01 | 9.99E-01 |
| P04430 | IGKV1-16 | -3.93 ± 1.5 | -4.54 ± 1.51 | -4.39 ± 1.36 | 6.72E-01 | 6.03E-01 | 9.95E-01 |
| P04433 | IGKV3-11 | -0.94 ± 0.71 | -0.92 ± 0.63 | -1.14 ± 0.54 | 7.16E-01 | 9.75E-01 | 9.99E-01 |
| P05019 | IGF1 | -0.3 ± 0.43 | -0.11 ± 0.55 | -0.48 ± 0.63 | 3.23E-01 | 5.97E-01 | 8.88E-01 |
| P05090 | APOD | -3.77 ± 0.82 | -3.87 ± 0.82 | -3.86 ± 0.87 | 9.53E-01 | 4.55E-01 | 9.99E-01 |
| P05155 | SERPING1 | -4.92 ± 0.76 | -4.95 ± 0.83 | -4.44 ± 0.77 | 4.19E-01 | 1.88E-01 | 8.98E-01 |
| P05452 | CLEC3B | 1.12 ± 0.64 | 1.04 ± 0.24 | 0.62 ± 0.3**^a^** | **3.72E-02** | 4.82E-01 | 6.75E-01 |
| P06331 | IGHV4-34 | -6.24 ± 0.55 | -6.2 ± 0.73 | -6.24 ± 0.55 | 9.89E-01 | 3.12E-01 | 9.99E-01 |
| P06681 | C2 | -4.46 ± 2.04 | -2.83 ± 2.26 | -3.4 ± 1.83 | 2.51E-01 | 3.29E-01 | 8.88E-01 |
| P06727 | APOA4 | 4.26 ± 1.22 | 5.14 ± 2.07 | 4.45 ± 1.69 | 4.52E-01 | 6.66E-01 | 8.98E-01 |
| P07357 | C8A | -5.9 ± 1.39 | -5.8 ± 0.94 | -5.83 ± 0.8 | 9.74E-01 | 6.25E-02 | 9.99E-01 |
| P07988 | SFTPB | -4.54 ± 1.28 | -4.16 ± 1.23 | -5.25 ± 1.23 | 1.64E-01 | 6.93E-01 | 8.88E-01 |
| P08697 | SERPINF2 | -1.52 ± 1.48 | -1.63 ± 1.78 | -2.71 ± 0.92 | 1.57E-01 | 1.38E-01 | 8.88E-01 |
| P09871 | C1S | -4.53 ± 1.16 | -4.83 ± 1.02 | -4.51 ± 1.21 | 7.16E-01 | 1.53E-02 | 9.99E-01 |
| P0CG47 | UBB | -5.12 ± 0.45 | -5.03 ± 0.47 | -5.49 ± 0.77 | 3.44E-01 | 6.86E-01 | 8.88E-01 |
| P0CG48 | UBC | -5.12 ± 0.45 | -5.03 ± 0.47 | -5.49 ± 0.77 | 3.44E-01 | 6.86E-01 | 8.88E-01 |
| P0DJI8 | SAA1 | 0.87 ± 1.56 | 1.67 ± 0.94 | 1.17 ± 1.47 | 3.71E-01 | 5.53E-01 | 8.88E-01 |
| P0DJI9 | SAA2 | -1.9 ± 0.46 | -3.16 ± 1.86 | -2.91 ± 1.72 | 1.84E-01 | 3.95E-01 | 8.88E-01 |
| P0DOX2 |  | -4.08 ± 0.69 | -3.91 ± 0.61 | -3.75 ± 0.84 | 6.40E-01 | 7.77E-01 | 9.71E-01 |
| P0DOX5 |  | 0.12 ± 0.63 | 0.35 ± 0.65 | 0.1 ± 0.85 | 6.35E-01 | 2.28E-01 | 9.71E-01 |
| P0DOX7 |  | -0.91 ± 0.9 | -1.17 ± 0.79 | -1.31 ± 0.8 | 5.62E-01 | 7.45E-01 | 9.45E-01 |
| P0DOX8 |  | 1.09 ± 0.74 | 1.17 ± 0.58 | 1.05 ± 0.45 | 9.06E-01 | 7.79E-01 | 9.99E-01 |
| P0DP04 | IGHV3-43D | -6.24 ± 0.55 | -6.2 ± 0.73 | -6.24 ± 0.55 | 9.89E-01 | 3.12E-01 | 9.99E-01 |
| P0DP06 | IGHV4-30-4 | -6.24 ± 0.55 | -6.2 ± 0.73 | -6.24 ± 0.55 | 9.89E-01 | 3.12E-01 | 9.99E-01 |
| P0DP07 | IGHV4-31 | -6.24 ± 0.55 | -6.2 ± 0.73 | -6.24 ± 0.55 | 9.89E-01 | 3.12E-01 | 9.99E-01 |
| P0DP08 | IGHV4-38-2 | -6.24 ± 0.55 | -6.2 ± 0.73 | -6.24 ± 0.55 | 9.89E-01 | 3.12E-01 | 9.99E-01 |
| P10720 | PF4V1 | -2.54 ± 1.03 | -2.48 ± 1.13 | -2.28 ± 1.18 | 8.63E-01 | 1.10E-01 | 9.99E-01 |
| P10909 | CLU | -6.27 ± 0.65 | -6.2 ± 0.84 | -6.38 ± 0.65 | 8.97E-01 | 3.20E-01 | 9.99E-01 |
| P12259 | F5 | -3.75 ± 1.62 | -3.83 ± 1.04 | -3.89 ± 1.73 | 9.79E-01 | 1.15E-01 | 9.99E-01 |
| P13645 | KRT10 | -4.01 ± 2.03 | -3.22 ± 3.28 | -4.17 ± 1.63 | 6.87E-01 | 3.54E-02 | 9.95E-01 |
| P13647 | KRT5 | -4.03 ± 1.79 | -4.2 ± 1.58 | -3.75 ± 1.2 | 8.06E-01 | 2.11E-01 | 9.99E-01 |
| P14625 | HSP90B1 | -5.02 ± 1.01 | -5.57 ± 0.78 | -5.88 ± 0.63 | 2.34E-01 | 9.69E-01 | 8.88E-01 |
| P16035 | TIMP2 | -5.48 ± 0.81 | -5.26 ± 1.15 | -5.87 ± 0.87 | 4.16E-01 | 3.95E-01 | 8.98E-01 |
| P16930 | FAH | -5.12 ± 0.45 | -5.03 ± 0.47 | -5.49 ± 0.77 | 3.44E-01 | 6.86E-01 | 8.88E-01 |
| P17936 | IGFBP3 | -4.09 ± 1.59 | -3.28 ± 0.89 | -2.63 ± 1.73 | 1.66E-01 | 9.59E-01 | 8.88E-01 |
| P18065 | IGFBP2 | -3.38 ± 1.13 | -2.68 ± 0.71 | -3.21 ± 1.32 | 2.57E-01 | 1.70E-01 | 8.88E-01 |
| P19652 | ORM2 | -2.03 ± 1.17 | -2.53 ± 1.39 | -2.65 ± 1.53 | 5.70E-01 | 5.24E-01 | 9.48E-01 |
| P20929 | NEB | -6.14 ± 0.53 | -6.32 ± 0.62 | -5.84 ± 0.45 | 3.58E-01 | 7.43E-01 | 8.88E-01 |
| P20930 | FLG | -3.33 ± 1.52 | -3.99 ± 1.67 | -4.36 ± 1.58 | 3.98E-01 | 5.18E-01 | 8.98E-01 |
| P25311 | AZGP1 | 0.25 ± 0.71 | 0.05 ± 0.53 | -0.05 ± 0.37 | 5.08E-01 | 6.62E-01 | 8.98E-01 |
| P30043 | BLVRB | -5.11 ± 1.04 | -4.47 ± 0.76 | -4.87 ± 1.09 | 4.35E-01 | 5.96E-02 | 8.98E-01 |
| P31151 | S100A7 | -2.62 ± 0.29 | -2.71 ± 0.27 | -2.82 ± 0.28 | 2.82E-01 | 1.45E-01 | 8.88E-01 |
| P35527 | KRT9 | -3.27 ± 2.32 | -3.87 ± 1.96 | -2.94 ± 2.94 | 7.41E-01 | 1.93E-01 | 9.99E-01 |
| P36980 | CFHR2 | -3 ± 1.13 | -3.19 ± 0.98 | -2.95 ± 1.35 | 8.68E-01 | 2.58E-01 | 9.99E-01 |
| P37802 | TAGLN2 | -3.54 ± 0.64 | -4.16 ± 0.73 | -3.86 ± 1.1 | 2.24E-01 | 8.56E-01 | 8.88E-01 |
| P49747 | COMP | -4.13 ± 1.49 | -4.02 ± 0.96 | -3.9 ± 0.69 | 9.27E-01 | 6.82E-01 | 9.99E-01 |
| P59665 | DEFA1 | -3.64 ± 1.23 | -4.53 ± 1.51 | -4.86 ± 1.79 | 2.29E-01 | 6.14E-01 | 8.88E-01 |
| P59666 | DEFA3 | -3.27 ± 0.82 | -2.92 ± 0.5 | -3.31 ± 0.98 | 4.48E-01 | 6.89E-01 | 8.98E-01 |
| P61626 | LYZ | 1.46 ± 0.71 | 1.62 ± 0.55 | 1.12 ± 0.52 | 1.96E-01 | 6.89E-01 | 8.88E-01 |
| P61769 | B2M | 0.97 ± 0.34 | 0.9 ± 0.8 | 0.16 ± 1.6 | 1.06E-01 | 1.08E-02 | 8.75E-01 |
| P62987 | UBA52 | -5.12 ± 0.45 | -5.03 ± 0.47 | -5.49 ± 0.77 | 3.44E-01 | 6.86E-01 | 8.88E-01 |
| P68363 | TUBA1B | -2.94 ± 0.97 | -2.85 ± 1.08 | -2.53 ± 0.84 | 6.41E-01 | 7.30E-01 | 9.71E-01 |
| P68871 | HBB | 6.64 ± 0.58 | 6.29 ± 0.63 | 6.5 ± 0.68 | 4.11E-01 | 1.98E-01 | 8.98E-01 |
| P69905 | HBA1 | 7.23 ± 0.45 | 6.62 ± 0.65 | 6.97 ± 0.6 | 5.19E-02 | 2.21E-01 | 6.75E-01 |
| P78386 | KRT85 | -5.81 ± 0.75 | -6.64 ± 0.71 | -5.52 ± 0.62 | 3.33E-01 | 2.71E-01 | 8.88E-01 |
| Q03591 | CFHR1 | -4.39 ± 1.39 | -3.67 ± 1.59 | -4.55 ± 1.17 | 3.33E-01 | 6.52E-01 | 8.88E-01 |
| Q04756 | HGFAC | -3.17 ± 2.42 | -3.6 ± 1.25 | -2.36 ± 1.22 | 3.04E-01 | 5.51E-01 | 8.88E-01 |
| Q05682 | CALD1 | -4.33 ± 2.01 | -3.33 ± 1.71 | -4.3 ± 1.41 | 3.67E-01 | 2.67E-01 | 8.88E-01 |
| Q08380 | LGALS3BP | -2.58 ± 1.8 | -1.3 ± 2.22 | -1.4 ± 1.93 | 2.55E-01 | 1.54E-01 | 8.88E-01 |
| Q13790 | APOF | -2.28 ± 1.49 | -0.9 ± 1.98 | -2.51 ± 2.11 | 1.14E-01 | 5.81E-01 | 8.75E-01 |
| Q14624 | ITIH4 | 3.92 ± 1.47 | 3.39 ± 1.23 | 4.22 ± 1.02 | 3.55E-01 | 8.87E-01 | 8.88E-01 |
| Q14766 | LTBP1 | -3.66 ± 0.75 | -4.13 ± 1.1 | -4.05 ± 0.77 | 6.26E-01 | 5.51E-01 | 9.71E-01 |
| Q15323 | KRT31 | -5.5 ± 0.9 | -5.64 ± 1.09 | -5.69 ± 0.96 | 9.23E-01 | 8.62E-01 | 9.99E-01 |
| Q15942 | ZYX | -0.26 ± 2.52 | 0.31 ± 2.23 | 0.27 ± 2.4 | 8.26E-01 | 1.71E-01 | 9.99E-01 |
| Q16610 | ECM1 | 0.04 ± 1.01 | -0.19 ± 0.97 | -0.18 ± 0.95 | 8.23E-01 | 6.60E-01 | 9.99E-01 |
| Q16627 | CCL14 | -5.95 ± 0.61 | -5.14 ± 1.01 | -5.48 ± 0.78 | 1.51E-01 | 2.22E-01 | 8.88E-01 |
| Q5TAX3 | TUT4 | -4.3 ± 1.4 | -3.79 ± 0.94 | -4.22 ± 0.35 | 6.29E-01 | 3.17E-01 | 9.71E-01 |
| Q5TZA2 | CROCC | -2.74 ± 0.87 | -2.61 ± 0.4 | -2.3 ± 0.48 | 4.64E-01 | 5.17E-01 | 8.98E-01 |
| Q86YW5 | TREML1 | -2.31 ± 1 | -1.1 ± 1.65 | -2.34 ± 1.29 | 3.93E-02 | 1.74E-02 | 6.75E-01 |
| Q92954 | PRG4 | 1.13 ± 0.63 | 0.98 ± 0.63 | 0.52 ± 0.61 | 1.12E-01 | 9.51E-01 | 8.75E-01 |
| Q96PD5 | PGLYRP2 | -0.29 ± 1.04 | -0.39 ± 0.79 | -0.62 ± 0.71 | 6.87E-01 | 1.41E-01 | 9.95E-01 |
| Q99969 | RARRES2 | -6.32 ± 0.62 | -5.12 ± 1.11**^a^** | -5.7 ± 0.87 | **4.48E-02** | 7.99E-01 | 6.75E-01 |
| Q9H4B7 | TUBB1 | -4.48 ± 0.67 | -4.9 ± 1.1 | -5.88 ± 1.02**^a^** | **5.29E-02** | 5.87E-01 | 6.75E-01 |
| Q9Y251 | HPSE | -4.92 ± 0.78 | -3.84 ± 1.17 | -4.3 ± 0.99 | 8.61E-02 | 3.53E-01 | 8.75E-01 |
| Q9Y490 | TLN1 | -2.95 ± 1.48 | -2.89 ± 1.35 | -2.54 ± 0.76 | 7.90E-01 | 1.84E-01 | 9.99E-01 |
| Q9Y613 | FHOD1 | -4.66 ± 0.26 | -2.1 ± 2.96**^a^** | -4.06 ± 2.05 | **4.09E-02** | 6.11E-01 | 6.75E-01 |

*Data are represented (log 2 transformed) as mean ± standard deviation. Statistical significance was calculated using a two-way ANOVA controlling for presence of type 2 diabetes mellitus (T2DM)* *as covariate. Tukey HSD post hoc test was used to estimate the statistical significance among groups. A p-value <0.05 was considered significant. Lowercase letters indicate post hoc analysis significance: “a” means different from controls “MASL”, and “b” means different from MASH F0-F2. MASL = Metabolic Dysfunction-Associated Steatotic Liver, MASH = Metabolic Dysfunction-Associated Steatohepatitis. The adjusted p-value was calculated using the false discovery rate (FDR) as described in the methods.*

**Supplementary Table 9. Differences in endogenous peptides in the untargeted cohort of healthy volunteers and patients with MASLD.**

| **Gene** | **Peptide** | **Control** | **MASLD** | **Log2FC** | **p (Disease)** | **p (T2DM)** | **FDR(Disease)** |
| --- | --- | --- | --- | --- | --- | --- | --- |
| A1AT | A.EDPQGDAAQKTDTSHHDQDHPTFNKITPNLAEFAFSLY.R | -8.43E+00 ± 1.69E+00 | -5.30E+00 ± 4.17E+00 | 3.129 | 3.60E-02 | 4.29E-01 | 5.32E-02 |
| A1AT | A.EDPQGDAAQKTDTSHHDQDHPTFNKITPNLAEFAFSLYR.Q | -8.49E+00 ± 1.24E+00 | -5.18E+00 ± 3.55E+00 | 3.31 | 9.54E-03 | 2.95E-01 | 2.03E-02 |
| A1AT | A.EDPQGDAAQKTDTSHHDQDHPTFNKITPNLAEFAFSLYRQLAH.Q | -8.12E+00 ± 1.76E+00 | -7.94E+00 ± 1.06E+00 | 0.172 | 7.17E-01 | 6.86E-01 | 7.57E-01 |
| A1AT | A.EDPQGDAAQKTDTSHHDQDHPTFNKITPNLAEFAFSLYRQLAHQ.S | -3.41E+00 ± 9.99E-01 | -6.72E+00 ± 2.31E+00 | -3.313 | 1.92E-04 | 5.07E-01 | 2.83E-03 |
| A1AT | G.DAAQKTDTSHHDQDHPTFNKITPNLAEFAFSLY.R | -8.52E+00 ± 1.02E+00 | -6.42E+00 ± 3.02E+00 | 2.102 | 4.63E-02 | 1.78E-01 | 6.66E-02 |
| A1AT | G.DAAQKTDTSHHDQDHPTFNKITPNLAEFAFSLYRQLAHQ.S | -5.31E+00 ± 1.12E+00 | -7.03E+00 ± 1.46E+00 | -1.719 | 2.67E-03 | 7.98E-01 | 1.04E-02 |
| A1AT | G.TEAAGAMFLEAIPMSIPPEVKFNKPFVFLMIE QNTKSPLFMGKVVNPTQK | -6.21E+00 ± 2.08E+00 | -7.27E+00 ± 2.77E+00 | -1.063 | 2.92E-01 | 2.66E-01 | 3.53E-01 |
| A1AT | K.SPLFM(+15.99)GKVVNPTQ.K | -8.93E+00 ± 7.85E-01 | -6.58E+00 ± 3.00E+00 | 2.349 | 2.83E-02 | 8.73E-01 | 4.36E-02 |
| A1AT | K.SPLFMGKVVNPTQK | -8.92E+00 ± 7.91E-01 | -6.48E+00 ± 2.35E+00 | 2.436 | 4.41E-03 | 3.81E-01 | 1.34E-02 |
| A1AT | R.SASLHLPKLSITGTYDL.K | -9.31E+00 ± 9.30E-01 | -6.58E+00 ± 2.81E+00 | 2.734 | 7.00E-03 | 3.56E-01 | 1.67E-02 |
| A1AT | R.SASLHLPKLSITGTYDLK.S | -9.27E+00 ± 6.78E-01 | -6.52E+00 ± 2.85E+00 | 2.752 | 5.65E-03 | 7.06E-02 | 1.52E-02 |
| A2MG | K.AIGYLNTGYQR.Q | -8.17E+00 ± 1.36E+00 | -6.87E+00 ± 1.85E+00 | 1.293 | 6.17E-02 | 8.37E-01 | 8.62E-02 |
| A2MG | L.VHVEEPHTETV.R | -8.84E+00 ± 5.76E-01 | -7.02E+00 ± 1.71E+00 | 1.823 | 3.74E-03 | 7.50E-01 | 1.22E-02 |
| A2MG | R.NQGNTWLTAFVLKTFAQAR.A | -8.61E+00 ± 7.17E-01 | -6.78E+00 ± 1.66E+00 | 1.825 | 2.53E-03 | 1.58E-01 | 1.01E-02 |
| ACTB | R.VAPEEHPVLLTEAPLNPK.A | -8.21E+00 ± 1.36E+00 | -6.97E+00 ± 1.65E+00 | 1.248 | 4.71E-02 | 7.41E-01 | 6.76E-02 |
| ACTG | R.VAPEEHPVLLTEAPLNPK.A | -8.30E+00 ± 1.38E+00 | -6.89E+00 ± 1.48E+00 | 1.414 | 1.56E-02 | 7.38E-01 | 2.87E-02 |
| AEGP | L.DGPDQQGAGV.D | -7.40E+00 ± 2.28E+00 | -7.88E+00 ± 9.91E-01 | -0.485 | 3.54E-01 | 5.73E-01 | 4.16E-01 |
| ALBU | M.DDFAAFVEKC(+57.02)C(+57.02)KA DDKETC(+57.02)FAEEGKKLV.A | -6.91E+00 ± 1.45E+00 | -6.93E+00 ± 1.59E+00 | -0.02 | 9.74E-01 | 2.47E-01 | 9.77E-01 |
| ALBU | R.DAHKSEVAHRFKDLGEENF.K | -8.53E+00 ± 1.01E+00 | -6.14E+00 ± 2.74E+00 | 2.394 | 1.31E-02 | 1.13E-01 | 2.52E-02 |
| ALBU | R.DAHKSEVAHRFKDLGEENFKALVLIAFAQYLQQ.C | -7.97E+00 ± 5.78E-01 | -6.24E+00 ± 3.08E+00 | 1.733 | 1.06E-01 | 4.56E-01 | 1.41E-01 |
| ANGT | N.KPEVLEVTLNRPFLFAVYDQSATALHFLGRVANPLSTA | -3.30E+00 ± 4.76E-01 | -6.67E+00 ± 1.98E+00 | -3.372 | 1.36E-05 | 4.74E-01 | 4.34E-04 |
| ANGT | R.VGEVLNSIFFELEADEREPTESTQQLNKPE VLEVTLNRPFLFAVYDQSATALHFLG.R | -8.78E+00 ± 8.97E-01 | -4.89E+00 ± 3.88E+00 | 3.894 | 5.46E-03 | 4.24E-01 | 1.48E-02 |
| APOA1 | A.ELQEGARQKLHELQEKLSPLGEEM.R | -8.78E+00 ± 4.25E-01 | -6.11E+00 ± 3.36E+00 | 2.673 | 2.52E-02 | 9.29E-01 | 4.00E-02 |
| APOA1 | A.EYHAKATEHLSTLSEKAKPALEDLRQGLLPVLESFKV.S | -9.22E+00 ± 9.10E-01 | -7.03E+00 ± 1.82E+00 | 2.197 | 1.43E-03 | 7.13E-01 | 7.14E-03 |
| APOA1 | A.EYHAKATEHLSTLSEKAKPALEDLRQGLLPVL ESFKVSFLSALEEYTKKLNTQ | -8.80E+00 ± 8.79E-01 | -3.12E+00 ± 5.03E+00 | 5.679 | 1.87E-03 | 3.19E-01 | 8.33E-03 |
| APOA1 | F.LSALEEYTKKLNTQ | -8.74E+00 ± 9.97E-01 | -7.26E+00 ± 1.89E+00 | 1.479 | 3.26E-02 | 6.84E-01 | 4.91E-02 |
| APOA1 | G.LLPVLESFKVSFLSALEEYTKKLNTQ | -8.79E+00 ± 4.85E-01 | -5.42E+00 ± 4.54E+00 | 3.373 | 3.58E-02 | 9.46E-01 | 5.31E-02 |
| APOA1 | K.ATEHLSTLSEKAKPALEDLRQGLLPVLESFKVSFLSALEEYTKKLNTQ | -8.51E+00 ± 7.96E-01 | -4.71E+00 ± 4.84E+00 | 3.805 | 2.67E-02 | 6.10E-01 | 4.19E-02 |
| APOA1 | K.LHELQEKLSPLGEEM.R | -9.27E+00 ± 7.70E-01 | -5.07E+00 ± 3.50E+00 | 4.195 | 9.14E-04 | 1.43E-01 | 5.91E-03 |
| APOA1 | K.LHELQEKLSPLGEEMRDRA.R | -8.85E+00 ± 6.94E-01 | -6.15E+00 ± 3.36E+00 | 2.698 | 2.41E-02 | 7.23E-01 | 3.86E-02 |
| APOA1 | K.VSFLSALEEYTKKLN.T | -8.90E+00 ± 6.93E-01 | -7.04E+00 ± 2.07E+00 | 1.854 | 1.32E-02 | 7.14E-01 | 2.53E-02 |
| APOA1 | K.VSFLSALEEYTKKLNT.Q | -8.86E+00 ± 6.48E-01 | -6.67E+00 ± 2.03E+00 | 2.191 | 2.65E-03 | 1.41E-01 | 1.03E-02 |
| APOA1 | K.VSFLSALEEYTKKLNTQ | -8.49E+00 ± 1.61E+00 | -3.99E+00 ± 3.97E+00 | 4.501 | 1.97E-03 | 2.13E-01 | 8.58E-03 |
| APOA1 | L.STLSEKAKPALEDL.R | -8.69E+00 ± 7.90E-01 | -6.24E+00 ± 2.35E+00 | 2.453 | 4.13E-03 | 3.51E-01 | 1.29E-02 |
| APOA1 | Q.DEPPQSPWDRVKDLATVYVDVLKDSGRDYVSQFEGSALG.K | -8.78E+00 ± 1.03E+00 | -6.61E+00 ± 2.74E+00 | 2.161 | 2.73E-02 | 3.51E-01 | 4.25E-02 |
| APOA1 | R.AELQEGARQKLHELQEKLSPLGEEM(+15.99)RD.R | -8.96E+00 ± 1.03E+00 | -5.07E+00 ± 4.84E+00 | 3.891 | 2.43E-02 | 8.82E-01 | 3.87E-02 |
| APOA1 | R.AELQEGARQKLHELQEKLSPLGEEM(+15.99)RDRA.R | -8.82E+00 ± 6.52E-01 | -6.18E+00 ± 4.24E+00 | 2.642 | 7.59E-02 | 8.19E-01 | 1.04E-01 |
| APOA1 | R.AELQEGARQKLHELQEKLSPLGEEM.R | -8.06E+00 ± 6.72E-01 | -5.41E+00 ± 4.69E+00 | 2.652 | 1.06E-01 | 8.58E-01 | 1.41E-01 |
| APOA1 | R.AELQEGARQKLHELQEKLSPLGEEMRD.R | -8.26E+00 ± 7.60E-01 | -5.31E+00 ± 3.63E+00 | 2.95 | 2.28E-02 | 9.80E-01 | 3.72E-02 |
| APOA1 | R.LAEYHAKATEHLSTLSEKAKPALEDLRQGLLP VLESFKVSFLSALEEYTKKLNTQ | -8.46E+00 ± 7.28E-01 | -4.89E+00 ± 4.35E+00 | 3.573 | 2.14E-02 | 9.55E-01 | 3.56E-02 |
| APOA1 | R.QGLLPVLESFKVSFLSALEEYTKKLNT.Q | -9.02E+00 ± 1.24E+00 | -5.50E+00 ± 3.38E+00 | 3.516 | 3.88E-03 | 1.77E-01 | 1.24E-02 |
| APOA1 | R.QGLLPVLESFKVSFLSALEEYTKKLNTQ | -7.89E+00 ± 2.74E+00 | -3.26E+00 ± 4.77E+00 | 4.63 | 7.77E-03 | 1.49E-01 | 1.78E-02 |
| APOA1 | V.LESFKVSFLSALEEYTKKLNTQ | -8.56E+00 ± 8.06E-01 | -5.75E+00 ± 4.26E+00 | 2.811 | 6.13E-02 | 9.78E-01 | 8.59E-02 |
| APOA1 | V.SFLSALEEYTKKLNTQ | -9.14E+00 ± 4.63E-01 | -6.60E+00 ± 2.55E+00 | 2.541 | 5.76E-03 | 5.48E-01 | 1.52E-02 |
| APOA2 | K.AGTELVNFLSYFVELGTQPAT.Q | -8.88E+00 ± 6.24E-01 | -7.25E+00 ± 1.67E+00 | 1.63 | 7.35E-03 | 5.53E-01 | 1.72E-02 |
| APOA2 | K.AGTELVNFLSYFVELGTQPATQ | -8.33E+00 ± 8.91E-01 | -6.84E+00 ± 2.02E+00 | 1.492 | 3.91E-02 | 3.80E-01 | 5.72E-02 |
| APOA2 | K.KAGTELVNFLSYFVELGTQPATQ | -8.96E+00 ± 9.64E-01 | -6.87E+00 ± 1.87E+00 | 2.084 | 3.04E-03 | 8.52E-01 | 1.11E-02 |
| APOA2 | K.SKEQLTPLIKKAGTELVNFLSYFVELGTQPATQ | -8.19E+00 ± 6.98E-01 | -7.03E+00 ± 1.85E+00 | 1.154 | 6.98E-02 | 9.63E-02 | 9.61E-02 |
| APOA2 | K.SPELQAEAKSYFEKSKEQLTPLIKKAGTELVNFLSYFVELGTQPATQ | -9.08E+00 ± 9.26E-01 | -6.51E+00 ± 3.23E+00 | 2.568 | 2.58E-02 | 5.66E-01 | 4.09E-02 |
| APOA2 | K.SYFEKSKEQLTPLIKKAGTELVNFLSYFVELGTQPAT.Q | -8.32E+00 ± 1.23E+00 | -6.79E+00 ± 2.75E+00 | 1.532 | 1.18E-01 | 5.50E-01 | 1.56E-01 |
| APOA2 | N.FLSYFVELGTQPATQ | -6.83E+00 ± 5.53E-01 | -7.02E+00 ± 1.55E+00 | -0.191 | 7.23E-01 | 7.02E-01 | 7.62E-01 |
| APOA4 | A.EVSADQVATVM(+15.99)WDYFSQLSNNAKEAVEHLQ.K | -9.10E+00 ± 9.46E-01 | -5.35E+00 ± 4.08E+00 | 3.751 | 1.08E-02 | 8.33E-01 | 2.24E-02 |
| APOA4 | A.EVSADQVATVMWDYFSQLSNNAKEAVEHLQ.K | -8.82E+00 ± 4.89E-01 | -4.87E+00 ± 4.27E+00 | 3.958 | 9.80E-03 | 8.43E-01 | 2.04E-02 |
| APOA4 | K.AKIDQNVEELKGRLTPYADEFKV.K | -8.61E+00 ± 8.87E-01 | -5.79E+00 ± 3.13E+00 | 2.82 | 1.23E-02 | 6.20E-01 | 2.45E-02 |
| APOA4 | K.SELTQQLNALFQDKLGEVNTYAGDLQ.K | -9.00E+00 ± 3.90E-01 | -5.42E+00 ± 4.04E+00 | 3.581 | 1.24E-02 | 3.79E-01 | 2.46E-02 |
| APOA4 | K.SLAELGGHLDQQVEEF.R | -8.33E+00 ± 1.63E+00 | -4.11E+00 ± 4.13E+00 | 4.225 | 5.19E-03 | 4.26E-01 | 1.47E-02 |
| APOA4 | K.SLAELGGHLDQQVEEFR.R | -8.81E+00 ± 9.45E-01 | -4.73E+00 ± 3.37E+00 | 4.088 | 1.10E-03 | 6.84E-01 | 6.45E-03 |
| APOA4 | K.SLAELGGHLDQQVEEFRR.R | -9.23E+00 ± 7.90E-01 | -6.75E+00 ± 2.19E+00 | 2.481 | 2.13E-03 | 4.50E-01 | 8.98E-03 |
| APOA4 | K.TLSLPELEQQQEQQQEQQQEQVQMLAPLES | -8.34E+00 ± 8.99E-01 | -5.81E+00 ± 3.14E+00 | 2.536 | 2.34E-02 | 4.47E-01 | 3.80E-02 |
| APOA4 | L.APLAEDVRGNL.R | -9.12E+00 ± 3.74E-01 | -6.79E+00 ± 1.87E+00 | 2.329 | 7.95E-04 | 7.26E-01 | 5.55E-03 |
| APOA4 | P.LAEDVRGNL.R | -8.40E+00 ± 7.98E-01 | -7.03E+00 ± 1.39E+00 | 1.367 | 7.90E-03 | 3.55E-01 | 1.78E-02 |
| APOA4 | Q.DTQEKLNHQLEGLTFQM(+15.99).K | -8.82E+00 ± 7.00E-01 | -5.17E+00 ± 4.47E+00 | 3.642 | 2.21E-02 | 7.50E-01 | 3.63E-02 |
| APOA4 | Q.EQQQEQQQEQVQMLAPLES | -8.69E+00 ± 1.01E+00 | -6.04E+00 ± 3.20E+00 | 2.657 | 1.92E-02 | 2.43E-01 | 3.27E-02 |
| APOA4 | R.ENADSLQASLRPH.A | -9.11E+00 ± 4.91E-01 | -6.99E+00 ± 2.61E+00 | 2.12 | 2.29E-02 | 8.56E-01 | 3.73E-02 |
| APOA4 | R.ENADSLQASLRPHADEL.K | -9.33E+00 ± 6.16E-01 | -6.25E+00 ± 3.75E+00 | 3.074 | 2.09E-02 | 4.99E-01 | 3.49E-02 |
| APOA4 | R.GNTEGLQKSLAELGGHLDQQVEEF.R | -6.96E+00 ± 1.73E+00 | -3.05E+00 ± 4.20E+00 | 3.911 | 7.34E-03 | 3.87E-02 | 1.72E-02 |
| APOA4 | R.GNTEGLQKSLAELGGHLDQQVEEFR.R | -8.90E+00 ± 9.88E-01 | -3.76E+00 ± 4.73E+00 | 5.141 | 2.35E-03 | 1.50E-01 | 9.76E-03 |
| APOA4 | R.GNTEGLQKSLAELGGHLDQQVEEFRR.R | -8.61E+00 ± 5.51E-01 | -5.38E+00 ± 3.21E+00 | 3.23 | 4.93E-03 | 3.14E-01 | 1.43E-02 |
| APOA4 | R.LAPLAEDVRGNLR.G | -9.35E+00 ± 8.21E-01 | -6.64E+00 ± 2.50E+00 | 2.709 | 2.96E-03 | 3.01E-01 | 1.08E-02 |
| APOA4 | R.LTPYADEFKVKIDQTVEEL.R | -9.28E+00 ± 7.87E-01 | -6.69E+00 ± 2.48E+00 | 2.593 | 4.22E-03 | 5.45E-01 | 1.30E-02 |
| APOA4 | R.LTPYADEFKVKIDQTVEELR.R | -8.98E+00 ± 7.55E-01 | -6.26E+00 ± 3.00E+00 | 2.72 | 1.10E-02 | 3.39E-01 | 2.25E-02 |
| APOA4 | R.RVEPYGENFNKALVQQMEQLR.Q | -9.17E+00 ± 9.84E-01 | -5.31E+00 ± 4.48E+00 | 3.862 | 1.61E-02 | 6.59E-01 | 2.92E-02 |
| APOA4 | R.SLAPYAQDTQEKLN.H | -9.09E+00 ± 9.19E-01 | -7.14E+00 ± 1.78E+00 | 1.945 | 3.56E-03 | 9.68E-01 | 1.21E-02 |
| APOA4 | R.SLAPYAQDTQEKLNHQLEGLTF.Q | -9.13E+00 ± 8.90E-01 | -6.19E+00 ± 3.22E+00 | 2.934 | 1.12E-02 | 4.85E-01 | 2.27E-02 |
| APOA4 | R.SLAPYAQDTQEKLNHQLEGLTFQM(+15.99).K | -8.57E+00 ± 9.38E-01 | -5.63E+00 ± 3.76E+00 | 2.949 | 2.81E-02 | 8.80E-01 | 4.35E-02 |
| APOA4 | R.SLAPYAQDTQEKLNHQLEGLTFQM(+15.99) KKNAEELKARISASAEELRQ.R | -9.05E+00 ± 7.12E-01 | -6.28E+00 ± 3.39E+00 | 2.766 | 2.24E-02 | 8.75E-01 | 3.67E-02 |
| APOA4 | R.SLAPYAQDTQEKLNHQLEGLTFQM(+15.99) KKNAEELKARISASAEELRQR.L | -9.08E+00 ± 9.03E-01 | -6.88E+00 ± 2.49E+00 | 2.197 | 1.51E-02 | 7.28E-01 | 2.80E-02 |
| APOA4 | R.SLAPYAQDTQEKLNHQLEGLTFQM(+15.99) KKNAEELKARISASAEELRQRLAPLAEDVRGNL.R | -8.42E+00 ± 1.07E+00 | -6.12E+00 ± 3.82E+00 | 2.297 | 8.58E-02 | 3.72E-01 | 1.16E-01 |
| APOA4 | R.SLAPYAQDTQEKLNHQLEGLTFQM(+15.99) KKNAEELKARISASAEELRQRLAPLAEDVRGNLR.G | -9.13E+00 ± 8.03E-01 | -6.09E+00 ± 3.51E+00 | 3.044 | 1.22E-02 | 7.84E-02 | 2.44E-02 |
| APOA4 | R.SLAPYAQDTQEKLNHQLEGLTFQM.K | -9.18E+00 ± 6.05E-01 | -5.70E+00 ± 4.12E+00 | 3.483 | 1.79E-02 | 9.80E-01 | 3.10E-02 |
| APOA4 | R.SLAPYAQDTQEKLNHQLEGLTFQMKKNAEELKA.R | -8.99E+00 ± 1.12E+00 | -4.85E+00 ± 4.33E+00 | 4.133 | 8.36E-03 | 7.48E-01 | 1.86E-02 |
| APOA4 | R.SLAPYAQDTQEKLNHQLEGLTFQMKKNAEELKARISASAEELR.Q | -8.62E+00 ± 9.52E-01 | -4.48E+00 ± 4.29E+00 | 4.137 | 7.79E-03 | 8.55E-01 | 1.78E-02 |
| APOA4 | R.SLAPYAQDTQEKLNHQLEGLTFQMKKNAEELKARISASAEELRQ.R | -8.77E+00 ± 1.33E+00 | -3.75E+00 ± 4.93E+00 | 5.014 | 4.98E-03 | 4.32E-01 | 1.44E-02 |
| APOA4 | R.SLAPYAQDTQEKLNHQLEGLTFQMKKNAEELKARISASAEELRQR.L | -8.80E+00 ± 9.35E-01 | -4.32E+00 ± 4.56E+00 | 4.476 | 5.30E-03 | 1.19E-01 | 1.47E-02 |
| APOA4 | R.SLAPYAQDTQEKLNHQLEGLTFQMKKNAEELKARISASAEE LRQRLAPLAEDVRGNL.R | -8.77E+00 ± 5.07E-01 | -3.28E+00 ± 5.02E+00 | 5.486 | 2.01E-03 | 1.06E-01 | 8.61E-03 |
| APOA4 | R.SLAPYAQDTQEKLNHQLEGLTFQMKKNAEELKA RISASAEELRQRLAPLAEDVRGNLR.G | -8.99E+00 ± 7.66E-01 | -4.02E+00 ± 5.28E+00 | 4.97 | 6.54E-03 | 7.04E-02 | 1.61E-02 |
| APOA4 | R.VLRENADSLQASLRPHADELKAKIDQNVEELKG.R | -8.73E+00 ± 7.73E-01 | -4.57E+00 ± 4.66E+00 | 4.161 | 1.27E-02 | 7.29E-01 | 2.48E-02 |
| APOA4 | R.VLRENADSLQASLRPHADELKAKIDQNVEELKGRL TPYADEFKVKIDQTVEELR.R | -9.00E+00 ± 9.07E-01 | -5.98E+00 ± 2.84E+00 | 3.019 | 3.65E-03 | 4.74E-01 | 1.22E-02 |
| APOA4 | Y.AQDTQEKLNHQLEGLTF.Q | -8.99E+00 ± 1.17E+00 | -7.39E+00 ± 2.11E+00 | 1.597 | 3.90E-02 | 9.15E-01 | 5.72E-02 |
| APOA4 | Y.AQDTQEKLNHQLEGLTFQM.K | -8.96E+00 ± 1.00E+00 | -6.25E+00 ± 2.94E+00 | 2.712 | 1.09E-02 | 6.61E-01 | 2.25E-02 |
| APOB | R.TLADLTLLDSPIKVPLLLSEPINIIDALEM(+15.99).R | -8.74E+00 ± 9.18E-01 | -6.92E+00 ± 1.52E+00 | 1.817 | 1.48E-03 | 2.23E-01 | 7.14E-03 |
| APOC1 | G.TPDVSSALDKLKEFGNTLEDKARELISRIKQSELSAKM (+15.99)REWFSETFQKVKEKLKIDS | -9.02E+00 ± 9.40E-01 | -3.22E+00 ± 5.02E+00 | 5.807 | 1.32E-03 | 1.66E-01 | 7.14E-03 |
| APOC1 | G.TPDVSSALDKLKEFGNTLEDKARELISRIKQSELSAK MREWFSETFQKVKEKLKIDS | 4.51E+00 ± 5.89E-01 | -1.18E+00 ± 5.24E+00 | -5.692 | 2.43E-03 | 2.12E-01 | 9.87E-03 |
| APOC1 | M.REWFSETFQKVKEKL.K | -9.14E+00 ± 6.17E-01 | -5.45E+00 ± 4.23E+00 | 3.691 | 1.31E-02 | 2.22E-01 | 2.52E-02 |
| APOC1 | P.DVSSALDKLKEFGNTLEDK.A | -8.60E+00 ± 8.45E-01 | -3.25E+00 ± 5.45E+00 | 5.351 | 5.35E-03 | 1.45E-01 | 1.47E-02 |
| APOC1 | P.DVSSALDKLKEFGNTLEDKA.R | -9.45E+00 ± 6.28E-01 | -6.32E+00 ± 3.18E+00 | 3.132 | 6.07E-03 | 3.67E-01 | 1.56E-02 |
| APOC1 | P.DVSSALDKLKEFGNTLEDKAREL.I | -9.17E+00 ± 9.59E-01 | -7.11E+00 ± 1.81E+00 | 2.063 | 2.47E-03 | 6.47E-01 | 9.92E-03 |
| APOC1 | P.DVSSALDKLKEFGNTLEDKARELIS.R | -9.23E+00 ± 1.02E+00 | -5.79E+00 ± 3.90E+00 | 3.437 | 1.37E-02 | 4.34E-01 | 2.59E-02 |
| APOC1 | P.DVSSALDKLKEFGNTLEDKARELISRIK.Q | -9.57E+00 ± 9.80E-01 | -4.57E+00 ± 4.75E+00 | 5 | 3.53E-03 | 3.29E-01 | 1.21E-02 |
| APOC1 | P.DVSSALDKLKEFGNTLEDKARELISRIKQSELSAKM (+15.99)REWFSETFQKVKEKLKIDS | -8.90E+00 ± 4.52E-01 | -5.31E+00 ± 4.40E+00 | 3.589 | 2.17E-02 | 8.57E-01 | 3.59E-02 |
| APOC1 | P.DVSSALDKLKEFGNTLEDKARELISRIKQSELS AKMREWFSETFQKVKEKLKIDS | 2.78E+00 ± 4.51E-01 | -2.48E-01 ± 3.57E+00 | -3.028 | 1.75E-02 | 8.53E-01 | 3.07E-02 |
| APOC1 | S.ELSAKM(+15.99)REWF.S | -9.00E+00 ± 8.23E-01 | -6.81E+00 ± 1.75E+00 | 2.196 | 9.57E-04 | 7.03E-01 | 5.91E-03 |
| APOC2 | G.TQQPQQDEM(+15.99)PSPTFLTQVKESLSSYWESA.K | -8.57E+00 ± 9.32E-01 | -6.98E+00 ± 2.66E+00 | 1.591 | 8.84E-02 | 3.63E-01 | 1.19E-01 |
| APOC2 | K.STAAM(+15.99)STYTGIFTDQVLSVLKGEE | -9.23E+00 ± 7.57E-01 | -6.29E+00 ± 3.12E+00 | 2.938 | 8.89E-03 | 5.55E-01 | 1.93E-02 |
| APOC2 | K.TAAQNLYEKTYLPAVDEKLRDLYSKSTAAM(+15.99) STYTGIFTDQVLSVLKGEE | -8.99E+00 ± 7.53E-01 | -6.13E+00 ± 2.94E+00 | 2.868 | 6.41E-03 | 2.78E-01 | 1.61E-02 |
| APOC2 | R.DLYSKSTAAM(+15.99)STYTG.I | -8.75E+00 ± 8.93E-01 | -4.95E+00 ± 4.58E+00 | 3.8 | 1.73E-02 | 1.46E-01 | 3.05E-02 |
| APOC2 | R.DLYSKSTAAM(+15.99)STYTGIFTDQVLSVLKGEE | -8.79E+00 ± 9.17E-01 | -5.27E+00 ± 3.32E+00 | 3.518 | 3.76E-03 | 7.06E-01 | 1.22E-02 |
| APOC2 | T.GIFTDQVLSVLKGEE | -8.13E+00 ± 1.28E+00 | -7.42E+00 ± 1.70E+00 | 0.705 | 2.62E-01 | 7.75E-01 | 3.21E-01 |
| APOC3 | A.SEAEDASLLSFM(+15.99)QGYM(+15.99).K | -8.68E+00 ± 1.20E+00 | -5.86E+00 ± 3.45E+00 | 2.816 | 2.36E-02 | 7.73E-01 | 3.81E-02 |
| APOC3 | A.SEAEDASLLSFM(+15.99)QGYM(+15.99)KHAT.K | -9.28E+00 ± 9.36E-01 | -4.38E+00 ± 4.22E+00 | 4.901 | 1.53E-03 | 4.29E-01 | 7.27E-03 |
| APOC3 | A.SEAEDASLLSFM(+15.99)QGYM(+15.99)KHATKT.A | -9.36E+00 ± 6.68E-01 | -6.04E+00 ± 2.59E+00 | 3.325 | 5.85E-04 | 5.75E-01 | 4.37E-03 |
| APOC3 | A.SEAEDASLLSFM(+15.99)QGYM(+15.99)KHATKTA.K | -9.02E+00 ± 5.38E-01 | -5.90E+00 ± 2.51E+00 | 3.119 | 8.52E-04 | 8.16E-01 | 5.65E-03 |
| APOC3 | A.SEAEDASLLSFM(+15.99)QGYM(+15.99)KHATKTAKDALSS.V | -8.85E+00 ± 4.78E-01 | -4.98E+00 ± 4.07E+00 | 3.874 | 7.13E-03 | 2.25E-01 | 1.69E-02 |
| APOC3 | A.SEAEDASLLSFM(+15.99)QGYM(+15.99)KHATKTAKDALSSVQES.Q | -9.01E+00 ± 9.66E-01 | -5.85E+00 ± 3.35E+00 | 3.163 | 8.63E-03 | 3.87E-01 | 1.89E-02 |
| APOC3 | A.SEAEDASLLSFM(+15.99)QGYM(+15.99)KHATKT AKDALSSVQESQVAQQA.R | -8.87E+00 ± 1.25E+00 | -4.06E+00 ± 4.54E+00 | 4.814 | 3.51E-03 | 3.96E-01 | 1.21E-02 |
| APOC3 | A.SEAEDASLLSFM(+15.99)QGYM(+15.99)KHAT KTAKDALSSVQESQVAQQAR.G | -8.62E+00 ± 4.56E-01 | -6.68E+00 ± 2.10E+00 | 1.941 | 9.58E-03 | 3.58E-01 | 2.03E-02 |
| APOC3 | A.SEAEDASLLSFM(+15.99)QGYM(+15.99)KHAT KTAKDALSSVQESQVAQQARGWVTDGFSSL.K | -9.27E+00 ± 1.03E+00 | -4.56E+00 ± 4.07E+00 | 4.71 | 1.70E-03 | 5.74E-01 | 7.90E-03 |
| APOC3 | A.SEAEDASLLSFM(+15.99)QGYMKHAT.K | -8.62E+00 ± 7.99E-01 | -6.44E+00 ± 3.27E+00 | 2.181 | 4.07E-02 | 1.02E-02 | 5.92E-02 |
| APOC3 | A.SEAEDASLLSFM(+15.99)QGYMKHATKTAKDALSSVQES.Q | -9.22E+00 ± 8.83E-01 | -6.44E+00 ± 2.56E+00 | 2.784 | 2.39E-03 | 9.90E-02 | 9.76E-03 |
| APOC3 | A.SEAEDASLLSFMQGYM(+15.99)KHAT.K | -9.23E+00 ± 9.70E-01 | -4.57E+00 ± 3.55E+00 | 4.657 | 4.47E-04 | 4.21E-01 | 3.77E-03 |
| APOC3 | A.SEAEDASLLSFMQGYM(+15.99)KHATKTA.K | -8.72E+00 ± 9.87E-01 | -6.62E+00 ± 2.52E+00 | 2.105 | 2.03E-02 | 4.91E-01 | 3.42E-02 |
| APOC3 | A.SEAEDASLLSFMQGYM(+15.99)KHATKTAKDALSSVQES.Q | -8.51E+00 ± 5.83E-01 | -6.21E+00 ± 2.54E+00 | 2.295 | 5.68E-03 | 4.56E-03 | 1.52E-02 |
| APOC3 | A.SEAEDASLLSFMQGYM(+15.99)KHATKTAKDALSSVQESQVAQQA.R | -9.04E+00 ± 8.32E-01 | -4.31E+00 ± 4.09E+00 | 4.73 | 1.42E-03 | 2.17E-01 | 7.14E-03 |
| APOC3 | A.SEAEDASLLSFMQGYMKHAT.K | -7.59E+00 ± 1.86E+00 | -7.69E+00 ± 9.70E-01 | -0.102 | 8.26E-01 | 7.04E-01 | 8.56E-01 |
| APOC3 | A.SEAEDASLLSFMQGYMKHATKTAKDALSSVQESQVAQQA.R | -3.86E+00 ± 1.00E+00 | -6.66E+00 ± 2.12E+00 | -2.798 | 5.36E-04 | 6.13E-01 | 4.12E-03 |
| APOC3 | A.SLLSFMQGYM(+15.99)KHATKTAKDALSSVQESQVAQQA.R | -9.18E+00 ± 1.08E+00 | -5.75E+00 ± 3.36E+00 | 3.429 | 5.25E-03 | 6.15E-01 | 1.47E-02 |
| APOC3 | A.TKTAKDALSSVQESQV.A | -8.43E+00 ± 5.81E-01 | -6.63E+00 ± 1.92E+00 | 1.806 | 9.59E-03 | 9.83E-01 | 2.03E-02 |
| APOC3 | K.HATKTAKDALSSVQESQVAQQA.R | -8.96E+00 ± 7.44E-01 | -5.13E+00 ± 3.71E+00 | 3.825 | 4.64E-03 | 8.47E-01 | 1.36E-02 |
| APOC3 | K.TAKDALSSVQESQVAQQA.R | -8.91E+00 ± 6.52E-01 | -4.65E+00 ± 3.48E+00 | 4.269 | 9.47E-04 | 9.69E-01 | 5.91E-03 |
| APOC3 | R.GWVTDGFSSLKDYWSTVKDKFSEF.W | -9.07E+00 ± 7.68E-01 | -4.88E+00 ± 3.89E+00 | 4.195 | 2.83E-03 | 3.04E-01 | 1.06E-02 |
| APOC4 | H.SLC(+57.02)PRLVC(+57.02)GDKDQG | -9.24E+00 ± 5.91E-01 | -6.74E+00 ± 2.34E+00 | 2.502 | 3.23E-03 | 3.81E-01 | 1.15E-02 |
| APOE | A.KVEQAVETEPEPEL.R | -8.19E+00 ± 1.00E+00 | -6.90E+00 ± 2.17E+00 | 1.293 | 9.60E-02 | 7.78E-01 | 1.29E-01 |
| APOE | A.KVEQAVETEPEPELR.Q | -9.22E+00 ± 8.10E-01 | -6.79E+00 ± 2.41E+00 | 2.426 | 5.90E-03 | 9.60E-01 | 1.54E-02 |
| APOE | A.TVGSLAGQPLQERAQAWGERL.R | -8.95E+00 ± 1.34E+00 | -4.80E+00 ± 3.54E+00 | 4.153 | 1.45E-03 | 2.65E-01 | 7.14E-03 |
| APOE | A.TVGSLAGQPLQERAQAWGERLR.A | -8.80E+00 ± 8.71E-01 | -6.07E+00 ± 2.55E+00 | 2.725 | 2.78E-03 | 9.75E-02 | 1.06E-02 |
| APOE | K.SWFEPLVEDM(+15.99)QRQWAGLVEKVQAAVGTSAAPVPSDNH | -7.29E+00 ± 2.30E+00 | -1.55E+00 ± 5.14E+00 | 5.736 | 2.37E-03 | 2.30E-01 | 9.76E-03 |
| APOE | K.SWFEPLVEDMQRQWAGLVEKVQAAVGTSAAPVPSDNH | -9.48E+00 ± 6.52E-01 | -6.33E+00 ± 2.33E+00 | 3.146 | 2.73E-04 | 1.80E-01 | 3.30E-03 |
| APOE | K.VQAAVGTSAAPVPSDNH | -8.98E+00 ± 4.42E-01 | -5.52E+00 ± 3.66E+00 | 3.458 | 8.09E-03 | 3.86E-01 | 1.81E-02 |
| APOE | R.AATVGSLAGQPLQERAQAWGERL.R | -8.89E+00 ± 9.95E-01 | -4.81E+00 ± 3.69E+00 | 4.081 | 1.63E-03 | 4.69E-02 | 7.64E-03 |
| APOE | R.AATVGSLAGQPLQERAQAWGERLR.A | -8.71E+00 ± 7.91E-01 | -4.93E+00 ± 3.29E+00 | 3.781 | 1.09E-03 | 4.19E-02 | 6.45E-03 |
| APOE | R.ARM(+15.99)EEM(+15.99)GSRTRDRLDEVKEQV AEVRAKLEEQAQQIRLQAEAFQARL.K | -9.07E+00 ± 9.05E-01 | -5.34E+00 ± 3.87E+00 | 3.726 | 7.87E-03 | 9.46E-01 | 1.78E-02 |
| APOE | R.ARMEEM(+15.99)GSRTRDRLDEVKEQVAEVRA KLEEQAQQIRLQAEAFQARL.K | -8.66E+00 ± 9.68E-01 | -6.26E+00 ± 2.75E+00 | 2.394 | 1.60E-02 | 5.95E-01 | 2.90E-02 |
| APOE | R.DRLDEVKEQVAEV.R | -9.07E+00 ± 6.17E-01 | -5.69E+00 ± 3.29E+00 | 3.382 | 4.66E-03 | 8.69E-01 | 1.36E-02 |
| APOE | R.ERLGPLVEQG.R | -8.97E+00 ± 8.51E-01 | -6.31E+00 ± 3.01E+00 | 2.655 | 1.45E-02 | 9.58E-01 | 2.73E-02 |
| APOE | R.QWAGLVEKVQAAVGTS.A | -8.74E+00 ± 9.58E-01 | -5.76E+00 ± 2.99E+00 | 2.974 | 6.38E-03 | 6.67E-01 | 1.61E-02 |
| APOE | R.QWAGLVEKVQAAVGTSAAPVPSDNH | -9.14E+00 ± 6.83E-01 | -5.12E+00 ± 3.27E+00 | 4.013 | 9.44E-04 | 7.23E-01 | 5.91E-03 |
| APOE | S.WFEPLVEDM(+15.99)Q.R | -8.92E+00 ± 7.50E-01 | -6.68E+00 ± 2.27E+00 | 2.244 | 6.85E-03 | 8.17E-01 | 1.64E-02 |
| APOF | K.SYDLDPGAGSLEI | -8.71E+00 ± 6.18E-01 | -6.76E+00 ± 2.10E+00 | 1.957 | 1.02E-02 | 7.84E-01 | 2.11E-02 |
| APOL1 | A.NLQSVPHASASRPR.V | -9.12E+00 ± 8.64E-01 | -6.33E+00 ± 2.47E+00 | 2.795 | 2.21E-03 | 7.04E-01 | 9.25E-03 |
| APOL1 | R.VTEPISAESGEQVER.V | -9.04E+00 ± 8.72E-01 | -7.16E+00 ± 1.70E+00 | 1.883 | 3.11E-03 | 7.23E-01 | 1.12E-02 |
| CAVN2 | A.SALVEGEIAEEAAEKAT.S | -7.52E+00 ± 1.53E+00 | -7.91E+00 ± 9.10E-01 | -0.391 | 3.45E-01 | 9.94E-01 | 4.07E-01 |
| CAVN2 | R.YEGSYALTSEEAERSDGDPVQPAVLQVHQTS | -5.35E+00 ± 1.49E+00 | -6.45E+00 ± 1.58E+00 | -1.1 | 7.35E-02 | 9.08E-01 | 1.01E-01 |
| CAVN2 | Y.ALTSEEAERSDGDPVQPAVLQVHQTS | -6.71E+00 ± 1.92E+00 | -7.18E+00 ± 1.21E+00 | -0.463 | 3.82E-01 | 3.75E-01 | 4.43E-01 |
| CLUS | F.FFPKSRIV.R | -8.98E+00 ± 8.29E-01 | -6.07E+00 ± 2.13E+00 | 2.907 | 3.10E-04 | 3.94E-01 | 3.43E-03 |
| CLUS | P.HFFFPK.S | -5.58E+00 ± 1.93E+00 | -4.66E+00 ± 3.92E+00 | 0.912 | 4.83E-01 | 2.50E-02 | 5.38E-01 |
| CLUS | P.HFFFPKSRIV.R | -6.98E+00 ± 2.24E+00 | -2.85E+00 ± 2.96E+00 | 4.131 | 3.51E-04 | 1.60E-01 | 3.66E-03 |
| CLUS | R.ASSIIDELFQDRFFTREPQDT.Y | -9.05E+00 ± 8.10E-01 | -6.77E+00 ± 1.73E+00 | 2.282 | 4.62E-04 | 2.65E-01 | 3.78E-03 |
| CLUS | R.ASSIIDELFQDRFFTREPQDTYHYLPFSLPH.R | -7.99E+00 ± 1.60E+00 | -4.52E+00 ± 3.52E+00 | 3.464 | 7.60E-03 | 6.69E-01 | 1.76E-02 |
| CLUS | R.ASSIIDELFQDRFFTREPQDTYHYLPFSLPHR.R | -9.32E+00 ± 6.18E-01 | -3.80E+00 ± 3.24E+00 | 5.52 | 1.41E-05 | 9.96E-01 | 4.34E-04 |
| CLUS | R.RPHFFFPKSRIV.R | -9.31E+00 ± 8.83E-01 | -6.15E+00 ± 2.76E+00 | 3.16 | 1.48E-03 | 1.22E-01 | 7.14E-03 |
| CO3 | E.TKENEGFTVTAEG.K | -8.92E+00 ± 1.07E+00 | -6.73E+00 ± 2.62E+00 | 2.187 | 2.02E-02 | 4.15E-01 | 3.42E-02 |
| CO3 | H.RIHWESASLL.R | -7.92E+00 ± 1.79E+00 | -3.81E+00 ± 3.01E+00 | 4.108 | 3.96E-04 | 3.23E-01 | 3.71E-03 |
| CO3 | I.HWESASLL.R | -1.55E+00 ± 1.19E+00 | -2.73E+00 ± 4.70E+00 | -1.183 | 4.48E-01 | 6.92E-02 | 5.08E-01 |
| CO3 | I.HWESASLLR.S | -8.75E+00 ± 7.74E-01 | -5.98E+00 ± 2.64E+00 | 2.765 | 4.03E-03 | 4.55E-01 | 1.27E-02 |
| CO3 | I.THRIHWESAS.L | -9.01E+00 ± 6.11E-01 | -4.02E+00 ± 4.83E+00 | 4.986 | 3.72E-03 | 1.90E-01 | 1.22E-02 |
| CO3 | I.THRIHWESASLL.R | -5.04E+00 ± 1.86E+00 | -3.85E+00 ± 4.07E+00 | 1.188 | 3.94E-01 | 1.21E-01 | 4.53E-01 |
| CO3 | K.EDIPPADLSDQVPDTESET.R | -8.77E+00 ± 6.42E-01 | -5.23E+00 ± 3.69E+00 | 3.538 | 6.65E-03 | 1.69E-01 | 1.61E-02 |
| CO3 | K.ENEGFTVTAEG.K | -9.18E+00 ± 1.25E+00 | -6.96E+00 ± 2.18E+00 | 2.22 | 5.99E-03 | 2.73E-01 | 1.56E-02 |
| CO3 | K.ITHRIHWESASLL.R | -8.72E+00 ± 1.34E+00 | -6.30E+00 ± 2.68E+00 | 2.419 | 1.11E-02 | 8.21E-02 | 2.27E-02 |
| CO3 | R.EGVQKEDIPPADLS.D | -9.07E+00 ± 7.78E-01 | -4.21E+00 ± 4.68E+00 | 4.861 | 3.48E-03 | 1.72E-01 | 1.21E-02 |
| CO3 | R.EGVQKEDIPPADLSDQVPDTESETR.I | -8.57E+00 ± 7.96E-01 | -5.18E+00 ± 3.91E+00 | 3.396 | 1.30E-02 | 1.47E-01 | 2.52E-02 |
| CO3 | R.FISLGEAC(+57.02)KKVFLDC(+57.02)C(+57.02)NYITEL.R | -8.85E+00 ± 8.87E-01 | -4.34E+00 ± 4.04E+00 | 4.512 | 2.09E-03 | 2.96E-01 | 8.86E-03 |
| CO3 | R.IHWESASLL.R | -8.30E+00 ± 1.69E+00 | -4.30E+00 ± 3.50E+00 | 4.004 | 1.30E-03 | 3.10E-02 | 7.14E-03 |
| CO3 | R.SEETKENEGFTVTAEG.K | -8.54E+00 ± 1.27E+00 | -2.13E+00 ± 4.63E+00 | 6.408 | 1.55E-04 | 7.25E-02 | 2.50E-03 |
| CO3 | R.SEETKENEGFTVTAEGK.G | -7.00E+00 ± 1.68E+00 | -6.38E+00 ± 1.95E+00 | 0.617 | 3.93E-01 | 2.68E-01 | 4.53E-01 |
| CO3 | R.SEETKENEGFTVTAEGKGQGTLSVV.T | -7.71E+00 ± 1.28E+00 | -7.33E+00 ± 2.90E+00 | 0.381 | 7.08E-01 | 7.89E-01 | 7.49E-01 |
| CO3 | R.SEETKENEGFTVTAEGKGQGTLSVVTM(+15.99)YHA.K | -8.44E+00 ± 1.01E+00 | -5.50E+00 ± 3.32E+00 | 2.947 | 1.26E-02 | 2.32E-01 | 2.48E-02 |
| CO3 | R.SNLDEDIIAEENIVS.R | -8.81E+00 ± 9.18E-01 | -4.69E+00 ± 4.01E+00 | 4.112 | 4.41E-03 | 3.20E-01 | 1.34E-02 |
| CO3 | R.SSKITHRIHWESAS.L | -8.78E+00 ± 1.06E+00 | -7.15E+00 ± 1.47E+00 | 1.628 | 3.48E-03 | 1.92E-01 | 1.21E-02 |
| CO3 | R.SSKITHRIHWESASL.L | -9.15E+00 ± 1.09E+00 | -4.88E+00 ± 3.47E+00 | 4.272 | 8.51E-04 | 2.93E-01 | 5.65E-03 |
| CO3 | R.SSKITHRIHWESASLL.R | -3.22E+00 ± 2.68E+00 | -2.20E+00 ± 4.99E+00 | 1.023 | 5.42E-01 | 3.82E-02 | 5.89E-01 |
| CO3 | R.SSKITHRIHWESASLLR.S | -5.49E+00 ± 1.31E+00 | -3.91E+00 ± 3.77E+00 | 1.577 | 2.20E-01 | 1.19E-01 | 2.76E-01 |
| CO3 | S.KITHRIHWESASLL.R | -6.86E+00 ± 1.31E+00 | -3.22E+00 ± 4.24E+00 | 3.635 | 8.56E-03 | 4.12E-03 | 1.88E-02 |
| CO3 | S.SKITHRIHWESASLL.R | -9.00E+00 ± 1.08E+00 | -3.50E+00 ± 4.72E+00 | 5.498 | 7.54E-04 | 1.63E-02 | 5.34E-03 |
| CO3 | T.HRIHWESASLL.R | -7.65E+00 ± 1.93E+00 | -4.26E+00 ± 3.35E+00 | 3.389 | 3.92E-03 | 1.68E-02 | 1.24E-02 |
| CO4A | K.DDPDAPLQPVTPLQLFEG.R | -8.52E+00 ± 7.03E-01 | -6.28E+00 ± 2.50E+00 | 2.242 | 1.27E-02 | 5.08E-01 | 2.48E-02 |
| CO4A | K.DDPDAPLQPVTPLQLFEGR.R | -6.78E+00 ± 1.41E+00 | -5.83E+00 ± 2.66E+00 | 0.954 | 3.02E-01 | 1.41E-01 | 3.63E-01 |
| CO4A | K.DDPDAPLQPVTPLQLFEGRRN.R | -3.99E+00 ± 3.66E+00 | -4.99E+00 ± 2.79E+00 | -1.002 | 3.61E-01 | 4.55E-02 | 4.23E-01 |
| CO4A | K.SHALQLNNRQIR.G | -9.05E+00 ± 7.77E-01 | -6.50E+00 ± 1.28E+00 | 2.548 | 2.14E-06 | 6.40E-01 | 1.42E-04 |
| CO4A | K.VLQIEKEGAIHREELVYELNPLDHR.G | -9.34E+00 ± 1.11E+00 | -6.05E+00 ± 2.84E+00 | 3.295 | 1.15E-03 | 4.59E-02 | 6.57E-03 |
| CO4A | L.EEELQFSLGSKI.N | -9.49E+00 ± 4.59E-01 | -6.92E+00 ± 1.67E+00 | 2.572 | 6.15E-05 | 4.86E-01 | 1.17E-03 |
| CO4A | L.EIPGNSDPNMIPDGDFNS.Y | -8.76E+00 ± 1.14E+00 | -6.80E+00 ± 2.38E+00 | 1.966 | 2.38E-02 | 8.09E-01 | 3.83E-02 |
| CO4A | R.GLEEELQFSLGS.K | -9.03E+00 ± 6.81E-01 | -6.37E+00 ± 3.12E+00 | 2.659 | 1.73E-02 | 8.18E-01 | 3.05E-02 |
| CO4A | R.GLEEELQFSLGSKI.N | -9.22E+00 ± 1.02E+00 | -6.57E+00 ± 2.53E+00 | 2.646 | 4.62E-03 | 8.29E-01 | 1.36E-02 |
| CO4A | R.GLEEELQFSLGSKINV.K | -7.72E+00 ± 1.39E+00 | -3.67E+00 ± 3.17E+00 | 4.047 | 7.53E-04 | 7.42E-01 | 5.34E-03 |
| CO4A | R.GLEEELQFSLGSKINVKVGGNS.K | -4.40E+00 ± 1.53E+00 | -3.29E+00 ± 2.12E+00 | 1.107 | 1.55E-01 | 4.97E-01 | 2.01E-01 |
| CO4A | R.GLEEELQFSLGSKINVKVGGNSKGTL.K | -9.22E+00 ± 9.13E-01 | -6.29E+00 ± 2.34E+00 | 2.926 | 5.03E-04 | 3.90E-02 | 4.04E-03 |
| CO4A | R.GLEEELQFSLGSKINVKVGGNSKGTLKVL.R | -8.73E+00 ± 1.42E+00 | -5.83E+00 ± 2.60E+00 | 2.896 | 2.60E-03 | 1.84E-01 | 1.03E-02 |
| CO4A | R.GSFEFPVGDAVSKVLQIEKEGAIH.R | -8.84E+00 ± 8.81E-01 | -6.39E+00 ± 2.82E+00 | 2.441 | 1.65E-02 | 8.44E-01 | 2.96E-02 |
| CO4A | R.NGFKSHALQLN.N | -8.66E+00 ± 1.15E+00 | -6.10E+00 ± 2.82E+00 | 2.56 | 9.32E-03 | 5.24E-02 | 2.00E-02 |
| CO4A | R.NGFKSHALQLNNR.Q | -8.69E+00 ± 4.30E-01 | -5.95E+00 ± 2.55E+00 | 2.744 | 2.92E-03 | 3.24E-01 | 1.08E-02 |
| CO4A | R.NGFKSHALQLNNRQ.I | -6.19E+00 ± 1.60E+00 | -6.56E+00 ± 2.18E+00 | -0.374 | 6.28E-01 | 1.00E-01 | 6.72E-01 |
| CO4A | R.NGFKSHALQLNNRQI.R | -3.53E+00 ± 1.37E+00 | -3.65E+00 ± 2.73E+00 | -0.125 | 8.96E-01 | 4.65E-01 | 9.17E-01 |
| CO4A | R.NGFKSHALQLNNRQIR.G | -4.11E+00 ± 2.42E+00 | -4.46E+00 ± 3.59E+00 | -0.354 | 7.74E-01 | 4.51E-02 | 8.07E-01 |
| CO4A | R.TLEIPGNSDPNM(+15.99)IPDGDFNS.Y | -9.02E+00 ± 7.99E-01 | -6.45E+00 ± 2.75E+00 | 2.566 | 7.71E-03 | 8.59E-02 | 1.78E-02 |
| CO4A | R.TLEIPGNSDPNM(+15.99)IPDGDFNSYV.R | -9.02E+00 ± 8.15E-01 | -6.44E+00 ± 3.04E+00 | 2.582 | 1.82E-02 | 8.69E-01 | 3.13E-02 |
| CO4A | R.TLEIPGNSDPNM(+15.99)IPDGDFNSYVR.V | -8.30E+00 ± 5.90E-01 | -5.51E+00 ± 2.85E+00 | 2.792 | 6.72E-03 | 7.10E-01 | 1.62E-02 |
| CO4A | R.TLEIPGNSDPNMIPDGDFN.S | -9.01E+00 ± 1.11E+00 | -6.42E+00 ± 2.88E+00 | 2.589 | 1.36E-02 | 9.44E-01 | 2.58E-02 |
| CO4A | R.TLEIPGNSDPNMIPDGDFNSYV.R | -8.79E+00 ± 9.58E-01 | -5.19E+00 ± 3.65E+00 | 3.606 | 6.49E-03 | 6.35E-01 | 1.61E-02 |
| CO4A | R.TLEIPGNSDPNMIPDGDFNSYVR.V | -8.67E+00 ± 8.72E-01 | -2.73E+00 ± 3.34E+00 | 5.931 | 4.50E-06 | 1.12E-01 | 2.09E-04 |
| CO4A3 | Q.GVPGAPGPPGEAGPRGELS.V | -8.61E+00 ± 1.61E+00 | -7.33E+00 ± 1.43E+00 | 1.287 | 2.60E-02 | 3.67E-01 | 4.09E-02 |
| CO4B | K.DDPDAPLQPVTPLQLFEG.R | -8.99E+00 ± 1.00E+00 | -6.38E+00 ± 2.54E+00 | 2.606 | 5.23E-03 | 6.52E-01 | 1.47E-02 |
| CO4B | K.DDPDAPLQPVTPLQLFEGR.R | -6.88E+00 ± 1.58E+00 | -5.78E+00 ± 2.53E+00 | 1.098 | 2.26E-01 | 3.28E-01 | 2.82E-01 |
| CO4B | K.DDPDAPLQPVTPLQLFEGRRN.R | -3.94E+00 ± 3.66E+00 | -5.13E+00 ± 2.94E+00 | -1.184 | 3.02E-01 | 6.22E-02 | 3.63E-01 |
| CO4B | K.SHALQLNNRQIR.G | -8.94E+00 ± 8.43E-01 | -6.94E+00 ± 1.62E+00 | 2.005 | 1.13E-03 | 5.82E-01 | 6.54E-03 |
| CO4B | K.VLQIEKEGAIHREELVYELNPLDHR.G | -9.13E+00 ± 8.19E-01 | -6.19E+00 ± 3.03E+00 | 2.937 | 5.46E-03 | 6.75E-02 | 1.48E-02 |
| CO4B | L.EEELQFSLGSKI.N | -9.24E+00 ± 1.01E+00 | -7.01E+00 ± 1.62E+00 | 2.234 | 4.41E-04 | 9.69E-01 | 3.77E-03 |
| CO4B | L.EIPGNSDPNMIPDGDFNS.Y | -8.86E+00 ± 8.68E-01 | -6.76E+00 ± 2.10E+00 | 2.103 | 6.65E-03 | 8.97E-01 | 1.61E-02 |
| CO4B | R.GLEEELQFSLGS.K | -8.76E+00 ± 7.51E-01 | -6.41E+00 ± 3.08E+00 | 2.351 | 3.17E-02 | 7.01E-01 | 4.81E-02 |
| CO4B | R.GLEEELQFSLGSKI.N | -8.73E+00 ± 7.66E-01 | -6.58E+00 ± 2.52E+00 | 2.152 | 1.74E-02 | 6.26E-01 | 3.06E-02 |
| CO4B | R.GLEEELQFSLGSKINV.K | -7.88E+00 ± 1.55E+00 | -3.65E+00 ± 3.10E+00 | 4.225 | 4.12E-04 | 9.44E-01 | 3.71E-03 |
| CO4B | R.GLEEELQFSLGSKINVKVGGNS.K | -4.40E+00 ± 1.53E+00 | -3.27E+00 ± 2.05E+00 | 1.13 | 1.37E-01 | 5.14E-01 | 1.78E-01 |
| CO4B | R.GLEEELQFSLGSKINVKVGGNSKGTL.K | -8.78E+00 ± 8.43E-01 | -6.26E+00 ± 2.26E+00 | 2.521 | 1.49E-03 | 3.29E-02 | 7.14E-03 |
| CO4B | R.GLEEELQFSLGSKINVKVGGNSKGTLKVL.R | -8.56E+00 ± 1.30E+00 | -5.98E+00 ± 2.72E+00 | 2.579 | 8.95E-03 | 2.26E-01 | 1.94E-02 |
| CO4B | R.GSFEFPVGDAVSKVLQIEKEGAIH.R | -9.17E+00 ± 1.37E+00 | -6.40E+00 ± 2.84E+00 | 2.763 | 8.41E-03 | 7.12E-01 | 1.86E-02 |
| CO4B | R.NGFKSHALQLN.N | -8.55E+00 ± 9.75E-01 | -5.97E+00 ± 2.61E+00 | 2.582 | 6.12E-03 | 2.26E-01 | 1.57E-02 |
| CO4B | R.NGFKSHALQLNNR.Q | -8.84E+00 ± 6.22E-01 | -5.90E+00 ± 2.54E+00 | 2.939 | 1.40E-03 | 1.85E-01 | 7.14E-03 |
| CO4B | R.NGFKSHALQLNNRQ.I | -6.27E+00 ± 1.80E+00 | -6.58E+00 ± 2.09E+00 | -0.311 | 6.86E-01 | 2.39E-01 | 7.29E-01 |
| CO4B | R.NGFKSHALQLNNRQI.R | -3.53E+00 ± 1.37E+00 | -3.65E+00 ± 2.72E+00 | -0.125 | 8.95E-01 | 3.63E-01 | 9.17E-01 |
| CO4B | R.NGFKSHALQLNNRQIR.G | -4.38E+00 ± 2.96E+00 | -4.49E+00 ± 3.53E+00 | -0.104 | 9.33E-01 | 2.58E-02 | 9.47E-01 |
| CO4B | R.TLEIPGNSDPNM(+15.99)IPDGDFNS.Y | -1.01E+01 ± 9.28E-01 | -6.57E+00 ± 2.86E+00 | 3.503 | 9.34E-04 | 3.26E-01 | 5.91E-03 |
| CO4B | R.TLEIPGNSDPNM(+15.99)IPDGDFNSYV.R | -9.05E+00 ± 6.87E-01 | -6.40E+00 ± 3.06E+00 | 2.65 | 1.57E-02 | 7.91E-01 | 2.87E-02 |
| CO4B | R.TLEIPGNSDPNM(+15.99)IPDGDFNSYVR.V | -8.90E+00 ± 5.13E-01 | -5.43E+00 ± 2.75E+00 | 3.469 | 6.93E-04 | 6.95E-01 | 5.04E-03 |
| CO4B | R.TLEIPGNSDPNMIPDGDFN.S | -9.14E+00 ± 5.85E-01 | -6.32E+00 ± 2.87E+00 | 2.816 | 6.81E-03 | 8.59E-01 | 1.64E-02 |
| CO4B | R.TLEIPGNSDPNMIPDGDFNSYV.R | -8.59E+00 ± 9.84E-01 | -5.15E+00 ± 3.65E+00 | 3.437 | 8.64E-03 | 3.44E-01 | 1.89E-02 |
| CO4B | R.TLEIPGNSDPNMIPDGDFNSYVR.V | -8.71E+00 ± 9.85E-01 | -2.66E+00 ± 3.24E+00 | 6.041 | 1.86E-06 | 8.97E-02 | 1.41E-04 |
| CP110 | G.QNAPVHR.L | -8.24E+00 ± 1.32E+00 | -7.74E+00 ± 8.79E-01 | 0.503 | 1.72E-01 | 7.37E-02 | 2.19E-01 |
| ECM1 | A.ASEGGFTATGQRQLRPEHFQEVGYAAPPSPPLS.R | -8.25E+00 ± 1.33E+00 | -6.68E+00 ± 1.71E+00 | 1.57 | 1.67E-02 | 8.27E-01 | 2.98E-02 |
| ECM1 | A.SEGGFTATGQRQLRPEHFQEVGYAAPPSPPLS.R | -7.82E+00 ± 1.23E+00 | -7.14E+00 ± 1.63E+00 | 0.678 | 2.62E-01 | 8.68E-01 | 3.21E-01 |
| ECM1 | R.QLRPEHFQEVGYAAPPSPPLS.R | -8.61E+00 ± 9.01E-01 | -7.02E+00 ± 1.32E+00 | 1.584 | 2.00E-03 | 9.73E-01 | 8.61E-03 |
| F13A | A.FGGRRAVPPNNSNAAEDDLPTVELQGVVPR.G | -8.86E+00 ± 7.88E-01 | -7.12E+00 ± 1.52E+00 | 1.746 | 9.15E-04 | 7.13E-03 | 5.91E-03 |
| F13A | A.VPPNNSNAAEDDLPTVEL.Q | -9.43E+00 ± 5.74E-01 | -6.09E+00 ± 1.84E+00 | 3.332 | 6.00E-06 | 7.91E-01 | 2.45E-04 |
| F13A | A.VPPNNSNAAEDDLPTVELQGVVPR.G | -8.21E+00 ± 2.08E+00 | -6.10E+00 ± 2.66E+00 | 2.111 | 3.22E-02 | 1.65E-01 | 4.87E-02 |
| F13A | R.AVPPNNSNAAEDDLPTVEL.Q | -9.08E+00 ± 1.34E+00 | -6.61E+00 ± 2.14E+00 | 2.47 | 1.75E-03 | 7.27E-02 | 8.06E-03 |
| F13A | R.AVPPNNSNAAEDDLPTVELQGV.V | -8.68E+00 ± 1.07E+00 | -6.58E+00 ± 1.94E+00 | 2.093 | 2.82E-03 | 5.59E-02 | 1.06E-02 |
| F13A | R.AVPPNNSNAAEDDLPTVELQGVVPR.G | -3.63E+00 ± 3.40E+00 | -3.23E+00 ± 3.09E+00 | 0.393 | 7.46E-01 | 9.42E-01 | 7.85E-01 |
| F13A | R.RAVPPNNSNAAEDDLPTVELQGVVPR.G | -3.62E+00 ± 2.16E+00 | -5.93E+00 ± 2.54E+00 | -2.308 | 1.53E-02 | 9.97E-02 | 2.83E-02 |
| F13A | R.TAFGGRRAVPPNNSNAAEDDLPTVELQGVVPR.G | -3.30E+00 ± 2.61E+00 | -4.91E+00 ± 3.57E+00 | -1.604 | 2.11E-01 | 1.40E-01 | 2.66E-01 |
| FA5 | R.HLSQDTGSPSGMRPWEDLPSQDTGSPS.R | -8.64E+00 ± 1.33E+00 | -6.83E+00 ± 2.43E+00 | 1.814 | 4.14E-02 | 7.42E-01 | 6.01E-02 |
| FETUA | R.AHYDLRHTFMGVVSLGSPSGEVSHPRKT.R | -6.90E+00 ± 1.47E+00 | -5.47E+00 ± 2.48E+00 | 1.434 | 9.48E-02 | 4.56E-02 | 1.27E-01 |
| FETUA | R.HTFM(+15.99)GVVSLGSPSGEVSHPR.K | -8.93E+00 ± 9.23E-01 | -6.53E+00 ± 2.31E+00 | 2.395 | 4.64E-03 | 3.60E-01 | 1.36E-02 |
| FETUA | R.HTFMGVVSLGSPSGEVSHPR.K | -9.30E+00 ± 1.01E+00 | -6.58E+00 ± 2.27E+00 | 2.713 | 1.49E-03 | 7.06E-01 | 7.14E-03 |
| FETUA | R.HTFMGVVSLGSPSGEVSHPRKT.R | -8.40E+00 ± 1.68E+00 | -6.13E+00 ± 2.30E+00 | 2.267 | 5.77E-03 | 1.65E-02 | 1.52E-02 |
| FETUA | R.TVVQPSVGAAAGPVVPPC(+57.02)PGRIRHFKV | -5.50E+00 ± 2.36E+00 | -6.27E+00 ± 2.29E+00 | -0.764 | 3.90E-01 | 7.92E-01 | 4.51E-01 |
| FHR1 | R.TTC(+57.02)WDGKLEYPTC(+57.02)A.K | -8.12E+00 ± 1.10E+00 | -7.17E+00 ± 1.73E+00 | 0.952 | 1.19E-01 | 8.87E-02 | 1.57E-01 |
| FIBA | A.DEAGSEADHEGTHSTKRGHAKSRPV.R | -3.05E+00 ± 5.68E-01 | -7.38E+00 ± 1.61E+00 | -4.324 | 1.46E-09 | 2.28E-01 | 7.74E-07 |
| FIBA | A.DSGEGDFLAEGGG.V | -6.18E+00 ± 1.55E+00 | -6.20E+00 ± 1.78E+00 | -0.013 | 9.85E-01 | 2.04E-01 | 9.86E-01 |
| FIBA | A.DSGEGDFLAEGGGV.R | 2.67E+00 ± 7.71E-01 | -1.81E+00 ± 5.11E+00 | -4.481 | 1.35E-02 | 3.91E-01 | 2.57E-02 |
| FIBA | A.DSGEGDFLAEGGGVR.G | 4.76E+00 ± 1.23E+00 | -2.60E+00 ± 5.04E+00 | -7.362 | 1.30E-04 | 7.66E-01 | 2.15E-03 |
| FIBA | A.DSGEGDFLAEGGGVRG.P | -2.63E+00 ± 1.58E+00 | -6.38E+00 ± 2.11E+00 | -3.742 | 1.83E-05 | 5.36E-01 | 4.64E-04 |
| FIBA | D.FLAEGGGVR.G | -7.26E+00 ± 1.46E+00 | -7.23E+00 ± 1.52E+00 | 0.032 | 9.56E-01 | 7.72E-01 | 9.63E-01 |
| FIBA | D.SGEGDFLAEGGG.V | -6.36E+00 ± 5.88E-01 | -6.66E+00 ± 1.49E+00 | -0.306 | 5.58E-01 | 6.96E-01 | 6.04E-01 |
| FIBA | D.SGEGDFLAEGGGV.R | 4.83E-01 ± 9.53E-01 | -2.52E+00 ± 4.37E+00 | -3 | 5.26E-02 | 9.44E-01 | 7.46E-02 |
| FIBA | D.SGEGDFLAEGGGVR.G | 2.57E+00 ± 1.09E+00 | -1.49E+00 ± 3.93E+00 | -4.064 | 4.65E-03 | 7.27E-01 | 1.36E-02 |
| FIBA | E.FVSETESRGSESGIFTNTKESSSHHPGIAEFPSRG.K | -8.85E+00 ± 8.95E-01 | -6.82E+00 ± 1.87E+00 | 2.029 | 3.64E-03 | 8.98E-01 | 1.22E-02 |
| FIBA | E.GDFLAEGGGV.R | -4.67E+00 ± 2.80E+00 | -4.54E+00 ± 2.75E+00 | 0.132 | 9.01E-01 | 7.39E-01 | 9.20E-01 |
| FIBA | E.GDFLAEGGGVR.G | 1.79E-01 ± 4.73E-01 | -4.77E+00 ± 3.08E+00 | -4.944 | 3.41E-05 | 9.25E-01 | 7.86E-04 |
| FIBA | F.FDTASTGKTFPG.F | -8.71E+00 ± 5.15E-01 | -4.82E+00 ± 4.68E+00 | 3.891 | 1.69E-02 | 1.68E-01 | 3.00E-02 |
| FIBA | F.TSSTSYNRGDSTFESKSY.K | -8.71E+00 ± 7.74E-01 | -7.19E+00 ± 1.38E+00 | 1.528 | 3.14E-03 | 4.75E-01 | 1.13E-02 |
| FIBA | F.TSSTSYNRGDSTFESKSYKM.A | -7.49E+00 ± 1.01E+00 | -7.84E+00 ± 1.05E+00 | -0.355 | 3.78E-01 | 9.91E-01 | 4.39E-01 |
| FIBA | F.TSSTSYNRGDSTFESKSYKMA.D | -6.78E+00 ± 1.73E+00 | -7.70E+00 ± 1.20E+00 | -0.92 | 7.64E-02 | 6.41E-01 | 1.04E-01 |
| FIBA | G.DFLAEGGGV.R | -3.58E+00 ± 2.05E+00 | -5.58E+00 ± 2.77E+00 | -2.004 | 5.14E-02 | 3.78E-01 | 7.31E-02 |
| FIBA | G.DFLAEGGGVR.G | 2.42E+00 ± 3.58E-01 | -4.48E+00 ± 4.61E+00 | -6.899 | 6.53E-05 | 2.00E-01 | 1.20E-03 |
| FIBA | G.EGDFLAEGGGV.R | 1.82E-01 ± 1.47E+00 | -3.92E+00 ± 4.21E+00 | -4.107 | 6.61E-03 | 2.19E-01 | 1.61E-02 |
| FIBA | G.EGDFLAEGGGVR.G | 3.33E+00 ± 2.25E+00 | -3.03E+00 ± 4.66E+00 | -6.351 | 3.71E-04 | 5.23E-01 | 3.67E-03 |
| FIBA | K.DSHSLTTNIMEILRGDFSSANN.R | -5.60E+00 ± 1.23E+00 | -6.08E+00 ± 2.48E+00 | -0.474 | 5.66E-01 | 3.24E-02 | 6.13E-01 |
| FIBA | K.DSHSLTTNIMEILRGDFSSANNR.D | -7.05E+00 ± 1.47E+00 | -6.55E+00 ± 2.79E+00 | 0.504 | 6.04E-01 | 1.80E-01 | 6.50E-01 |
| FIBA | K.ESSSHHPGIAEFPS.R | -9.26E+00 ± 3.70E-01 | -6.89E+00 ± 1.42E+00 | 2.366 | 1.81E-05 | 3.91E-01 | 4.64E-04 |
| FIBA | K.ESSSHHPGIAEFPSRG.K | -9.17E+00 ± 9.31E-01 | -6.92E+00 ± 1.59E+00 | 2.25 | 2.86E-04 | 6.76E-01 | 3.30E-03 |
| FIBA | K.MADEAGSEADHEGTHSTKRGHAKSRPV.R | -5.20E+00 ± 2.14E+00 | -6.43E+00 ± 2.54E+00 | -1.229 | 1.96E-01 | 4.71E-01 | 2.48E-01 |
| FIBA | K.QFTSSTSYNRGDSTFES.K | -8.85E+00 ± 5.32E-01 | -6.40E+00 ± 2.43E+00 | 2.454 | 5.18E-03 | 4.58E-01 | 1.47E-02 |
| FIBA | K.QFTSSTSYNRGDSTFESKS.Y | -8.14E+00 ± 1.26E+00 | -6.55E+00 ± 1.99E+00 | 1.591 | 3.10E-02 | 6.73E-01 | 4.74E-02 |
| FIBA | K.QFTSSTSYNRGDSTFESKSY.K | -8.44E+00 ± 1.18E+00 | -6.51E+00 ± 2.23E+00 | 1.927 | 1.62E-02 | 1.59E-01 | 2.92E-02 |
| FIBA | K.QFTSSTSYNRGDSTFESKSYKMA.D | -6.67E+00 ± 2.09E+00 | -7.50E+00 ± 1.33E+00 | -0.823 | 1.61E-01 | 4.17E-01 | 2.06E-01 |
| FIBA | K.QFTSSTSYNRGDSTFESKSYKMADEAGSEADHEGTHST.K | -8.55E+00 ± 1.08E+00 | -6.50E+00 ± 2.77E+00 | 2.056 | 3.49E-02 | 1.97E-01 | 5.22E-02 |
| FIBA | K.SSSYSKQFTSSTSY.N | -6.40E+00 ± 2.00E+00 | -5.90E+00 ± 2.98E+00 | 0.498 | 6.39E-01 | 2.10E-01 | 6.83E-01 |
| FIBA | K.SSSYSKQFTSSTSYN.R | -8.81E+00 ± 4.00E-01 | -2.04E+00 ± 4.97E+00 | 6.763 | 2.56E-04 | 3.81E-01 | 3.30E-03 |
| FIBA | K.SSSYSKQFTSSTSYNRGDST.F | -6.63E+00 ± 1.19E+00 | -6.67E+00 ± 1.93E+00 | -0.04 | 9.54E-01 | 4.96E-01 | 9.63E-01 |
| FIBA | K.SSSYSKQFTSSTSYNRGDSTF.E | -7.07E+00 ± 1.08E+00 | -4.47E+00 ± 2.54E+00 | 2.597 | 5.27E-03 | 3.68E-01 | 1.47E-02 |
| FIBA | K.SSSYSKQFTSSTSYNRGDSTFES.K | -1.38E+00 ± 2.60E-01 | -2.79E+00 ± 3.37E+00 | -1.409 | 2.24E-01 | 4.48E-01 | 2.80E-01 |
| FIBA | K.SSSYSKQFTSSTSYNRGDSTFESK.S | -6.25E+00 ± 4.86E-01 | -5.75E+00 ± 2.24E+00 | 0.501 | 4.97E-01 | 5.65E-02 | 5.48E-01 |
| FIBA | K.SSSYSKQFTSSTSYNRGDSTFESKS.Y | -2.18E+00 ± 1.62E+00 | -4.17E+00 ± 2.77E+00 | -1.983 | 4.76E-02 | 2.78E-01 | 6.81E-02 |
| FIBA | K.SSSYSKQFTSSTSYNRGDSTFESKSY.K | -3.29E-01 ± 5.27E-01 | -2.75E+00 ± 3.44E+00 | -2.422 | 4.42E-02 | 3.67E-01 | 6.38E-02 |
| FIBA | K.SSSYSKQFTSSTSYNRGDSTFESKSYKM(+15.99).A | -5.00E+00 ± 8.22E-01 | -7.10E+00 ± 1.57E+00 | -2.096 | 5.18E-04 | 6.01E-01 | 4.10E-03 |
| FIBA | K.SSSYSKQFTSSTSYNRGDSTFESKSYKM(+15.99)A.D | -3.81E+00 ± 7.02E-01 | -7.04E+00 ± 1.96E+00 | -3.234 | 2.47E-05 | 4.11E-01 | 5.96E-04 |
| FIBA | K.SSSYSKQFTSSTSYNRGDSTFESKSYKM.A | -3.64E-01 ± 4.91E-01 | -5.63E+00 ± 2.98E+00 | -5.271 | 7.89E-06 | 8.76E-01 | 2.99E-04 |
| FIBA | K.SSSYSKQFTSSTSYNRGDSTFESKSYKMA.D | -2.16E-01 ± 2.10E+00 | -4.85E+00 ± 2.73E+00 | -4.631 | 3.66E-05 | 4.76E-01 | 8.10E-04 |
| FIBA | K.SSSYSKQFTSSTSYNRGDSTFESKSYKMADEAGSEADHEGTHST.K | -8.25E+00 ± 1.54E+00 | -4.07E+00 ± 3.05E+00 | 4.185 | 3.80E-04 | 8.82E-01 | 3.67E-03 |
| FIBA | K.SSSYSKQFTSSTSYNRGDSTFESKSYKMADEAGSEA DHEGTHSTKRGHA.K | -3.06E+00 ± 7.61E-01 | -6.12E+00 ± 2.95E+00 | -3.065 | 3.76E-03 | 2.26E-01 | 1.22E-02 |
| FIBA | K.SSSYSKQFTSSTSYNRGDSTFESKSYKMADEAGSEA DHEGTHSTKRGHAKSRPV.R | -5.59E+00 ± 1.91E+00 | -7.66E+00 ± 1.14E+00 | -2.063 | 2.46E-04 | 7.63E-01 | 3.26E-03 |
| FIBA | K.SYKM(+15.99)ADEAGSEADHEGTHST.K | -9.44E+00 ± 8.28E-01 | -6.59E+00 ± 2.27E+00 | 2.849 | 4.30E-04 | 2.94E-02 | 3.77E-03 |
| FIBA | K.SYKMADEAGSEADHEGTHST.K | -8.54E+00 ± 1.08E+00 | -1.80E+00 ± 3.43E+00 | 6.743 | 1.38E-06 | 6.12E-01 | 1.22E-04 |
| FIBA | K.SYKMADEAGSEADHEGTHSTK.R | -9.66E+00 ± 5.87E-01 | -6.72E+00 ± 1.74E+00 | 2.937 | 1.55E-05 | 3.13E-01 | 4.34E-04 |
| FIBA | K.SYKMADEAGSEADHEGTHSTKR.G | -9.00E+00 ± 6.81E-01 | -7.35E+00 ± 1.14E+00 | 1.655 | 2.29E-04 | 6.68E-01 | 3.19E-03 |
| FIBA | K.SYKMADEAGSEADHEGTHSTKRGHA.K | -4.79E+00 ± 1.93E+00 | -5.83E+00 ± 2.57E+00 | -1.041 | 2.73E-01 | 9.35E-01 | 3.33E-01 |
| FIBA | K.SYKMADEAGSEADHEGTHSTKRGHAKSRPV.R | -3.83E+00 ± 8.43E-01 | -5.23E+00 ± 3.44E+00 | -1.399 | 2.42E-01 | 5.37E-01 | 2.99E-01 |
| FIBA | K.TFPGFFSPM(+15.99)LGEFVSETES.R | -8.67E+00 ± 1.01E+00 | -6.75E+00 ± 2.18E+00 | 1.926 | 1.32E-02 | 1.39E-01 | 2.53E-02 |
| FIBA | K.TFPGFFSPM(+15.99)LGEFVSETESRGSESG.I | -9.11E+00 ± 7.64E-01 | -6.58E+00 ± 2.15E+00 | 2.526 | 1.34E-03 | 2.01E-01 | 7.14E-03 |
| FIBA | K.TFPGFFSPM(+15.99)LGEFVSETESRGSESGIFTNTKESSSHHPGIAEFPSRG.K | -1.80E+00 ± 9.78E-01 | -8.79E-01 ± 3.64E+00 | 0.921 | 4.55E-01 | 1.67E-01 | 5.14E-01 |
| FIBA | K.TFPGFFSPMLGEFVSETESRGSESGIFTNTKESSSHHPGIAEFPS.R | -6.70E+00 ± 2.94E+00 | -5.61E+00 ± 3.15E+00 | 1.093 | 3.57E-01 | 3.40E-01 | 4.19E-01 |
| FIBA | K.TFPGFFSPMLGEFVSETESRGSESGIFTNTKESSSHHPGIAEFPSRG.K | 5.42E-01 ± 4.92E-01 | -1.79E+00 ± 3.12E+00 | -2.33 | 3.50E-02 | 8.53E-01 | 5.22E-02 |
| FIBA | K.TFPGFFSPMLGEFVSETESRGSESGIFTNTKESSSHHPGIAEFPSRGK.S | -2.90E+00 ± 1.54E+00 | -5.13E+00 ± 2.86E+00 | -2.238 | 2.96E-02 | 2.01E-01 | 4.54E-02 |
| FIBA | M.KPVPDLVPGNF.K | -8.41E+00 ± 1.71E+00 | -6.19E+00 ± 2.78E+00 | 2.219 | 2.14E-02 | 2.13E-02 | 3.56E-02 |
| FIBA | M.LGEFVSETESRGSESGIFTNTKESSSHHPGIAEFPSRG.K | -8.21E+00 ± 1.40E+00 | -6.78E+00 ± 1.84E+00 | 1.43 | 3.93E-02 | 5.86E-01 | 5.73E-02 |
| FIBA | N.PDWGTFEEVSGNVSPGTR.R | -9.18E+00 ± 8.75E-01 | -5.72E+00 ± 3.53E+00 | 3.461 | 6.30E-03 | 3.00E-01 | 1.60E-02 |
| FIBA | P.MLGEFVSETESRGSESGIFTNTKESSSHHPGIAEFPSRG.K | -6.91E+00 ± 2.18E+00 | -5.19E+00 ± 2.33E+00 | 1.721 | 5.67E-02 | 6.51E-01 | 8.01E-02 |
| FIBA | P.VPDLVPGNF.K | -8.17E+00 ± 1.24E+00 | -7.70E+00 ± 8.33E-01 | 0.472 | 1.91E-01 | 5.91E-01 | 2.42E-01 |
| FIBA | Q.FTSSTSYNRGDSTFES.K | -7.67E+00 ± 1.30E+00 | -6.86E+00 ± 2.23E+00 | 0.816 | 3.05E-01 | 4.21E-01 | 3.66E-01 |
| FIBA | Q.FTSSTSYNRGDSTFESKS.Y | -8.04E+00 ± 1.65E+00 | -7.22E+00 ± 1.57E+00 | 0.817 | 1.82E-01 | 3.56E-01 | 2.31E-01 |
| FIBA | Q.FTSSTSYNRGDSTFESKSY.K | -6.37E+00 ± 7.97E-01 | -6.79E+00 ± 1.80E+00 | -0.424 | 4.84E-01 | 6.00E-02 | 5.38E-01 |
| FIBA | Q.FTSSTSYNRGDSTFESKSYKM.A | -5.33E+00 ± 1.27E+00 | -7.15E+00 ± 1.69E+00 | -1.825 | 4.54E-03 | 2.13E-01 | 1.36E-02 |
| FIBA | Q.FTSSTSYNRGDSTFESKSYKMA.D | -4.27E+00 ± 1.73E+00 | -7.28E+00 ± 1.54E+00 | -3.005 | 1.48E-05 | 8.46E-01 | 4.34E-04 |
| FIBA | R.GDSTFESKSYKMADEAGSEADHEGTHST.K | -8.89E+00 ± 6.81E-01 | -3.50E+00 ± 3.13E+00 | 5.39 | 1.09E-05 | 4.46E-01 | 3.85E-04 |
| FIBA | R.GKSSSYSKQFTSSTSYNRGDSTFES.K | -9.05E+00 ± 9.18E-01 | -6.60E+00 ± 1.29E+00 | 2.453 | 3.36E-06 | 1.03E-01 | 1.79E-04 |
| FIBA | R.GSAGHWTSESSVSGSTGQWHSESGSFRPDSPGSGNA.R | -8.43E+00 ± 9.91E-01 | -5.72E+00 ± 3.01E+00 | 2.712 | 9.74E-03 | 7.57E-02 | 2.04E-02 |
| FIBA | R.GSESGIFTNTKESSSHHPGIAEFPS.R | -8.88E+00 ± 1.15E+00 | -6.53E+00 ± 2.84E+00 | 2.356 | 2.20E-02 | 6.67E-01 | 3.63E-02 |
| FIBA | R.GSESGIFTNTKESSSHHPGIAEFPSRG.K | -7.44E+00 ± 2.08E+00 | -5.11E+00 ± 3.48E+00 | 2.338 | 6.54E-02 | 5.50E-01 | 9.09E-02 |
| FIBA | R.HRHPDEAAFFDTASTGKTFPGFFSPMLGEFVSETESR GSESGIFTNTKESSSHHPGIAEFPSRG.K | -2.57E+00 ± 1.09E+00 | -3.80E+00 ± 3.09E+00 | -1.23 | 2.57E-01 | 7.59E-01 | 3.17E-01 |
| FIBA | R.REYHTEKLVTSKGDKEL.R | -8.10E+00 ± 2.19E+00 | -7.94E+00 ± 1.07E+00 | 0.162 | 7.58E-01 | 6.40E-01 | 7.94E-01 |
| FIBA | S.GEGDFLAEGGGV.R | -1.48E+00 ± 1.80E+00 | -4.08E+00 ± 3.85E+00 | -2.593 | 6.12E-02 | 6.64E-01 | 8.59E-02 |
| FIBA | S.GEGDFLAEGGGVR.G | 1.27E+00 ± 1.21E+00 | -4.72E+00 ± 3.82E+00 | -5.994 | 4.62E-05 | 3.76E-01 | 9.28E-04 |
| FIBA | S.SSYSKQFTSSTSY.N | -6.75E+00 ± 1.55E+00 | -5.73E+00 ± 1.90E+00 | 1.02 | 1.47E-01 | 2.59E-01 | 1.90E-01 |
| FIBA | S.SSYSKQFTSSTSYN.R | -8.94E+00 ± 1.16E+00 | -5.17E+00 ± 2.49E+00 | 3.773 | 8.80E-05 | 2.79E-01 | 1.51E-03 |
| FIBA | S.SSYSKQFTSSTSYNRGDST.F | -7.05E+00 ± 6.14E-01 | -7.68E+00 ± 1.05E+00 | -0.631 | 1.00E-01 | 9.62E-01 | 1.34E-01 |
| FIBA | S.SSYSKQFTSSTSYNRGDSTF.E | -9.09E+00 ± 1.01E+00 | -4.17E+00 ± 4.37E+00 | 4.915 | 1.76E-03 | 1.71E-01 | 8.06E-03 |
| FIBA | S.SSYSKQFTSSTSYNRGDSTFE.S | -8.59E+00 ± 6.87E-01 | -4.03E+00 ± 5.41E+00 | 4.56 | 1.77E-02 | 5.75E-01 | 3.08E-02 |
| FIBA | S.SSYSKQFTSSTSYNRGDSTFES.K | -5.32E+00 ± 9.42E-01 | -5.73E+00 ± 1.42E+00 | -0.407 | 4.29E-01 | 9.36E-01 | 4.89E-01 |
| FIBA | S.SSYSKQFTSSTSYNRGDSTFESKSY.K | -6.95E+00 ± 1.77E+00 | -7.29E+00 ± 1.28E+00 | -0.338 | 5.25E-01 | 3.78E-01 | 5.74E-01 |
| FIBA | S.STSYNRGDSTFESKSY.K | -8.04E+00 ± 1.13E+00 | -6.77E+00 ± 1.05E+00 | 1.265 | 3.51E-03 | 7.73E-01 | 1.21E-02 |
| FIBA | S.STSYNRGDSTFESKSYKM.A | -7.93E+00 ± 1.59E+00 | -7.80E+00 ± 1.28E+00 | 0.129 | 8.03E-01 | 9.94E-01 | 8.35E-01 |
| FIBA | S.SYSKQFTSSTSYN.R | -8.47E+00 ± 1.11E+00 | -6.56E+00 ± 1.85E+00 | 1.914 | 5.43E-03 | 2.99E-01 | 1.48E-02 |
| FIBA | S.SYSKQFTSSTSYNRGDSTFES.K | -7.65E+00 ± 8.51E-01 | -4.59E+00 ± 3.70E+00 | 3.062 | 1.58E-02 | 5.86E-02 | 2.88E-02 |
| FIBA | S.SYSKQFTSSTSYNRGDSTFESKS.Y | -8.64E+00 ± 8.64E-01 | -7.30E+00 ± 1.25E+00 | 1.335 | 5.21E-03 | 9.37E-01 | 1.47E-02 |
| FIBA | S.SYSKQFTSSTSYNRGDSTFESKSY.K | -8.22E+00 ± 1.28E+00 | -7.29E+00 ± 1.31E+00 | 0.928 | 6.44E-02 | 1.85E-01 | 8.97E-02 |
| FIBA | S.YKMADEAGSEADHEGTHST.K | -9.29E+00 ± 8.28E-01 | -6.45E+00 ± 1.84E+00 | 2.835 | 4.72E-05 | 7.34E-02 | 9.28E-04 |
| FIBA | T.ADSGEGDFLAEGGGV.R | -2.57E+00 ± 2.32E+00 | -2.58E+00 ± 2.63E+00 | -0.006 | 9.95E-01 | 7.35E-01 | 9.95E-01 |
| FIBA | T.ADSGEGDFLAEGGGVR.G | 5.86E-01 ± 9.55E-01 | -3.41E+00 ± 2.62E+00 | -3.993 | 8.21E-05 | 6.06E-01 | 1.45E-03 |
| FIBA | T.ADSGEGDFLAEGGGVRGPR.V | -8.07E+00 ± 1.14E+00 | -7.36E+00 ± 1.47E+00 | 0.709 | 1.91E-01 | 4.28E-01 | 2.42E-01 |
| FIBA | T.NTKESSSHHPGIAEFPSRG.K | -8.65E+00 ± 7.59E-01 | -5.56E+00 ± 2.61E+00 | 3.089 | 1.43E-03 | 6.97E-01 | 7.14E-03 |
| FIBA | T.SYNRGDSTFESKSYKMADEAGSEADHEGTHST.K | -8.75E+00 ± 7.90E-01 | -4.47E+00 ± 4.47E+00 | 4.285 | 6.56E-03 | 1.68E-01 | 1.61E-02 |
| FIBA | V.SETESRGSESGIFTNTKESSSHHPGIAEFPSRG.K | -4.79E+00 ± 2.44E+00 | -3.76E+00 ± 2.72E+00 | 1.036 | 3.14E-01 | 6.00E-01 | 3.75E-01 |
| FIBA | V.SGSTGQWHSESGSFRPDSPGSGNA.R | -8.70E+00 ± 1.02E+00 | -6.84E+00 ± 1.95E+00 | 1.865 | 8.97E-03 | 2.51E-01 | 1.94E-02 |
| FIBA | V.SGSTGQWHSESGSFRPDSPGSGNARPNNPDWGTF.E | -8.51E+00 ± 8.59E-01 | -6.25E+00 ± 2.60E+00 | 2.259 | 1.23E-02 | 7.34E-02 | 2.45E-02 |
| FIBA | V.SGSTGQWHSESGSFRPDSPGSGNARPNNPDWGTFEEV.S | -7.34E+00 ± 1.03E+00 | -7.75E+00 ± 1.05E+00 | -0.411 | 3.07E-01 | 5.71E-01 | 3.67E-01 |
| FIBA | W.TADSGEGDFLAEGGGVR.G | -8.77E+00 ± 1.63E+00 | -5.03E+00 ± 4.27E+00 | 3.736 | 1.46E-02 | 2.85E-01 | 2.73E-02 |
| FIBA | Y.NRGDSTFESKSYKM.A | -8.50E+00 ± 2.07E+00 | -5.39E+00 ± 3.71E+00 | 3.109 | 2.07E-02 | 2.31E-01 | 3.47E-02 |
| FIBA | Y.NRGDSTFESKSYKMA.D | -4.91E+00 ± 1.29E+00 | -6.57E+00 ± 2.46E+00 | -1.667 | 5.03E-02 | 4.98E-02 | 7.17E-02 |
| FIBB | D.NEEGFFSA.R | -8.49E+00 ± 1.53E+00 | -6.22E+00 ± 2.64E+00 | 2.268 | 1.80E-02 | 1.78E-01 | 3.10E-02 |
| FIBB | G.VNDNEEGFF.S | -9.13E+00 ± 5.72E-01 | -7.15E+00 ± 1.75E+00 | 1.983 | 1.30E-03 | 3.56E-02 | 7.14E-03 |
| FIBB | G.VNDNEEGFFS.A | -7.13E+00 ± 9.59E-01 | -7.19E+00 ± 1.20E+00 | -0.066 | 8.81E-01 | 7.56E-01 | 9.07E-01 |
| FIBB | G.VNDNEEGFFSA.R | -5.18E+00 ± 1.67E+00 | -7.33E+00 ± 1.49E+00 | -2.153 | 6.47E-04 | 4.37E-01 | 4.77E-03 |
| FIBB | K.REEAPSLRPAPPPISGGGY.R | -8.26E+00 ± 1.61E+00 | -7.27E+00 ± 1.34E+00 | 0.994 | 6.74E-02 | 3.30E-01 | 9.34E-02 |
| FIBB | N.DNEEGFF.S | -2.48E+00 ± 5.92E-01 | -5.71E+00 ± 2.39E+00 | -3.232 | 3.02E-04 | 3.89E-01 | 3.41E-03 |
| FIBB | N.DNEEGFFS.A | -5.90E+00 ± 2.12E+00 | -7.35E+00 ± 1.74E+00 | -1.457 | 3.85E-02 | 1.67E-01 | 5.66E-02 |
| FIBB | N.DNEEGFFSA.R | -4.32E+00 ± 1.63E+00 | -6.52E+00 ± 2.41E+00 | -2.205 | 1.48E-02 | 4.81E-01 | 2.76E-02 |
| FIBB | Q.GVNDNEEGFFSA.R | -7.75E+00 ± 1.79E+00 | -6.39E+00 ± 1.92E+00 | 1.358 | 5.76E-02 | 7.19E-02 | 8.11E-02 |
| FIBB | R.EEAPSLRPAPPPISGGGY.R | -8.38E+00 ± 1.17E+00 | -5.88E+00 ± 3.38E+00 | 2.501 | 3.17E-02 | 7.49E-02 | 4.81E-02 |
| FIBB | R.GHRPLDKKREEAPSLRPAPPPISGGGY.R | -3.89E+00 ± 9.05E-01 | -6.45E+00 ± 2.23E+00 | -2.559 | 1.91E-03 | 3.53E-01 | 8.45E-03 |
| FIBB | S.QGVNDNEEGFF.S | -7.19E+00 ± 2.79E+00 | -6.28E+00 ± 2.89E+00 | 0.903 | 4.05E-01 | 1.89E-01 | 4.65E-01 |
| FIBB | S.QGVNDNEEGFFSAR.G | -8.27E+00 ± 1.20E+00 | -5.73E+00 ± 3.31E+00 | 2.535 | 2.78E-02 | 1.06E-01 | 4.31E-02 |
| FIBB | V.NDNEEGFF.S | -7.88E-01 ± 4.90E-01 | -4.48E+00 ± 2.97E+00 | -3.69 | 8.27E-04 | 8.75E-01 | 5.63E-03 |
| FIBB | V.NDNEEGFFS.A | -4.20E+00 ± 1.01E+00 | -6.54E+00 ± 2.32E+00 | -2.335 | 6.17E-03 | 4.93E-01 | 1.58E-02 |
| FIBB | V.NDNEEGFFSA.R | -1.84E+00 ± 3.61E+00 | -5.49E+00 ± 3.67E+00 | -3.654 | 1.26E-02 | 7.89E-01 | 2.48E-02 |
| FIBG | K.AIQLTYNPDESSKPNMIDAATLK.S | -7.46E+00 ± 1.09E+00 | -8.02E+00 ± 1.01E+00 | -0.565 | 1.57E-01 | 6.55E-01 | 2.03E-01 |
| FINC | R.TNTNVNC(+57.02)PIEC(+57.02)FMPLDVQADREDSRE | -9.13E+00 ± 5.91E-01 | -7.15E+00 ± 1.96E+00 | 1.975 | 5.83E-03 | 7.14E-01 | 1.53E-02 |
| INS | A.FVNQHLC(+57.02)GSHLVEALYLVC(+57.02)GERGFFYTPK.T | -6.88E+00 ± 8.89E-01 | -7.67E+00 ± 1.18E+00 | -0.795 | 6.77E-02 | 2.38E-01 | 9.36E-02 |
| ITIH4 | A.AGSRMNFRPGVLSSRQLGLPGPPDVPDHAAYHPF.R | -8.00E+00 ± 1.60E+00 | -6.76E+00 ± 2.13E+00 | 1.241 | 1.18E-01 | 6.49E-01 | 1.56E-01 |
| ITIH4 | A.GAAGSRMNFRPGVLSSRQLGLPGPPDVPDHAAYHPF.R | -4.45E+00 ± 2.46E+00 | -4.35E+00 ± 3.22E+00 | 0.099 | 9.32E-01 | 1.80E-01 | 9.47E-01 |
| ITIH4 | F.RPGVLSSRQLGLPGPPDVPDHAAYHPF.R | -7.61E+00 ± 2.27E+00 | -7.92E+00 ± 9.33E-01 | -0.307 | 5.42E-01 | 3.39E-01 | 5.89E-01 |
| ITIH4 | G.SEMVVAGKLQ.D | -8.27E+00 ± 1.21E+00 | -7.28E+00 ± 1.78E+00 | 0.988 | 1.32E-01 | 9.10E-01 | 1.73E-01 |
| ITIH4 | K.GSEMVVAGKLQ.D | -8.76E+00 ± 1.33E+00 | -6.92E+00 ± 1.60E+00 | 1.844 | 3.42E-03 | 9.34E-01 | 1.21E-02 |
| ITIH4 | K.GSEMVVAGKLQD.R | -8.97E+00 ± 5.39E-01 | -7.13E+00 ± 1.30E+00 | 1.836 | 1.89E-04 | 2.71E-01 | 2.83E-03 |
| ITIH4 | K.GSEMVVAGKLQDR.G | -8.69E+00 ± 1.30E+00 | -7.01E+00 ± 1.14E+00 | 1.681 | 5.77E-04 | 9.11E-01 | 4.37E-03 |
| ITIH4 | K.YYLQGAKIPKPEAS.F | -8.86E+00 ± 5.17E-01 | -6.71E+00 ± 2.28E+00 | 2.152 | 8.17E-03 | 3.31E-01 | 1.82E-02 |
| ITIH4 | K.YYLQGAKIPKPEASFSPR.R | -6.69E+00 ± 2.33E+00 | -6.23E+00 ± 2.44E+00 | 0.468 | 6.09E-01 | 2.75E-01 | 6.55E-01 |
| ITIH4 | L.GLPGPPDVPDH.A | -8.80E+00 ± 1.12E+00 | -6.58E+00 ± 2.30E+00 | 2.217 | 7.23E-03 | 1.06E-01 | 1.71E-02 |
| ITIH4 | L.GLPGPPDVPDHA.A | -8.75E+00 ± 1.17E+00 | -6.50E+00 ± 2.79E+00 | 2.241 | 2.66E-02 | 7.68E-01 | 4.18E-02 |
| ITIH4 | L.GLPGPPDVPDHAA.Y | -9.14E+00 ± 6.03E-01 | -6.65E+00 ± 1.89E+00 | 2.493 | 4.12E-04 | 3.24E-01 | 3.71E-03 |
| ITIH4 | L.GLPGPPDVPDHAAY.H | -9.03E+00 ± 5.86E-01 | -5.46E+00 ± 3.48E+00 | 3.567 | 3.59E-03 | 1.06E-01 | 1.21E-02 |
| ITIH4 | L.GLPGPPDVPDHAAYHPF.R | -4.80E+00 ± 2.00E+00 | -3.71E+00 ± 3.40E+00 | 1.088 | 3.74E-01 | 9.76E-01 | 4.35E-01 |
| ITIH4 | L.PGPPDVPDHAAY.H | -9.00E+00 ± 6.24E-01 | -6.91E+00 ± 1.50E+00 | 2.081 | 2.85E-04 | 8.16E-01 | 3.30E-03 |
| ITIH4 | L.PGPPDVPDHAAYHPF.R | -8.92E+00 ± 1.00E+00 | -6.66E+00 ± 1.88E+00 | 2.262 | 1.21E-03 | 1.53E-01 | 6.82E-03 |
| ITIH4 | L.SSRQLGLPGPPDVPDHAAYHPF.R | -7.37E+00 ± 2.11E+00 | -5.43E+00 ± 2.08E+00 | 1.936 | 1.97E-02 | 6.47E-01 | 3.35E-02 |
| ITIH4 | M.NFRPGVLSSRQLGLPGPPDVPDHAAYHPF.R | -7.83E+00 ± 2.44E+00 | -7.14E+00 ± 1.63E+00 | 0.687 | 3.31E-01 | 7.34E-01 | 3.92E-01 |
| ITIH4 | N.FRPGVLSSRQLGLPGPPDVPDHAAYHPF.R | -5.49E+00 ± 2.50E+00 | -5.68E+00 ± 2.67E+00 | -0.189 | 8.51E-01 | 5.69E-01 | 8.80E-01 |
| ITIH4 | P.GPPDVPDHAAYHPF.R | -7.33E+00 ± 1.97E+00 | -5.89E+00 ± 1.70E+00 | 1.435 | 3.75E-02 | 3.79E-01 | 5.53E-02 |
| ITIH4 | P.GVLSSRQLGLPGPPDVPDHAA.Y | -7.86E+00 ± 1.00E+00 | -6.87E+00 ± 1.94E+00 | 0.992 | 1.46E-01 | 1.70E-01 | 1.89E-01 |
| ITIH4 | P.GVLSSRQLGLPGPPDVPDHAAYHPF.R | -6.71E+00 ± 3.18E+00 | -7.76E+00 ± 1.46E+00 | -1.055 | 1.60E-01 | 4.42E-01 | 2.05E-01 |
| ITIH4 | R.GWNRQAGAAGSRMNFRPGVLSSRQLGLPGPPDVPDHAAYHPF.R | -8.06E+00 ± 2.43E+00 | -7.92E+00 ± 9.97E-01 | 0.14 | 7.96E-01 | 6.32E-01 | 8.29E-01 |
| ITIH4 | R.M(+15.99)NFRPGVLSSRQLGLPGPPDVPDHAAYHPF.R | -6.12E+00 ± 2.53E+00 | -4.25E+00 ± 3.18E+00 | 1.866 | 1.18E-01 | 5.89E-01 | 1.56E-01 |
| ITIH4 | R.MNFRPGVLSSRQLGLPGPPDVPDHAA.Y | -7.43E+00 ± 2.23E+00 | -6.44E+00 ± 2.04E+00 | 0.991 | 2.16E-01 | 4.07E-01 | 2.72E-01 |
| ITIH4 | R.MNFRPGVLSSRQLGLPGPPDVPDHAAYHPF.R | -3.27E+00 ± 2.60E+00 | -2.14E+00 ± 3.46E+00 | 1.134 | 3.74E-01 | 8.18E-01 | 4.35E-01 |
| ITIH4 | R.MNFRPGVLSSRQLGLPGPPDVPDHAAYHPFR.R | -8.05E+00 ± 1.82E+00 | -3.77E+00 ± 3.13E+00 | 4.281 | 4.35E-04 | 8.25E-01 | 3.77E-03 |
| ITIH4 | R.NVHSGSTFF.K | -6.62E+00 ± 1.24E+00 | -5.96E+00 ± 2.10E+00 | 0.661 | 3.74E-01 | 2.55E-01 | 4.35E-01 |
| ITIH4 | R.NVHSGSTFFKYYLQGA.K | -8.99E+00 ± 6.39E-01 | -6.35E+00 ± 2.49E+00 | 2.639 | 3.75E-03 | 6.36E-01 | 1.22E-02 |
| ITIH4 | R.NVHSGSTFFKYYLQGAKIPKPEA.S | -6.56E+00 ± 2.76E+00 | -3.32E+00 ± 3.57E+00 | 3.244 | 1.77E-02 | 9.64E-01 | 3.08E-02 |
| ITIH4 | R.NVHSGSTFFKYYLQGAKIPKPEASFSPR.R | -1.10E+00 ± 2.69E+00 | -1.71E+00 ± 3.77E+00 | -0.605 | 6.57E-01 | 3.59E-01 | 7.00E-01 |
| ITIH4 | R.PGVLSSRQLGLPGPPDVPDHAA.Y | -9.42E+00 ± 6.71E-01 | -4.77E+00 ± 3.88E+00 | 4.65 | 1.10E-03 | 3.94E-01 | 6.45E-03 |
| ITIH4 | R.PGVLSSRQLGLPGPPDVPDHAAYHP.F | -9.19E+00 ± 1.13E+00 | -6.32E+00 ± 2.48E+00 | 2.864 | 1.46E-03 | 8.42E-02 | 7.14E-03 |
| ITIH4 | R.PGVLSSRQLGLPGPPDVPDHAAYHPF.R | -4.25E+00 ± 2.73E+00 | -1.76E+00 ± 2.39E+00 | 2.485 | 1.18E-02 | 7.32E-01 | 2.38E-02 |
| ITIH4 | R.PGVLSSRQLGLPGPPDVPDHAAYHPFR.R | -8.44E+00 ± 1.48E+00 | -5.40E+00 ± 3.19E+00 | 3.034 | 9.72E-03 | 7.42E-01 | 2.04E-02 |
| ITIH4 | R.QAGAAGSRM(+15.99)NFRPGVLSSRQLGLPGPPDVPDHAAYHPF.R | -5.87E+00 ± 2.53E+00 | -5.41E+00 ± 3.39E+00 | 0.455 | 7.04E-01 | 8.65E-02 | 7.46E-01 |
| ITIH4 | R.QAGAAGSRMNFRPGVLS.S | -8.59E+00 ± 1.75E+00 | -7.19E+00 ± 1.59E+00 | 1.398 | 2.82E-02 | 3.54E-01 | 4.35E-02 |
| ITIH4 | R.QAGAAGSRMNFRPGVLSSRQLGLPGPPDVPDHAAYHPF.R | -2.55E+00 ± 3.83E+00 | -4.42E+00 ± 4.79E+00 | -1.87 | 2.86E-01 | 2.40E-01 | 3.47E-01 |
| ITIH4 | R.QAGAAGSRMNFRPGVLSSRQLGLPGPPDVPDHAAYHPFR.R | -6.98E+00 ± 2.49E+00 | -6.25E+00 ± 2.40E+00 | 0.725 | 4.37E-01 | 9.08E-01 | 4.96E-01 |
| ITIH4 | R.QLGLPGPPDVPDH.A | -8.79E+00 ± 9.41E-01 | -6.87E+00 ± 2.19E+00 | 1.923 | 1.55E-02 | 5.67E-01 | 2.87E-02 |
| ITIH4 | R.QLGLPGPPDVPDHA.A | -8.76E+00 ± 1.17E+00 | -4.00E+00 ± 4.56E+00 | 4.756 | 2.62E-03 | 3.52E-02 | 1.03E-02 |
| ITIH4 | R.QLGLPGPPDVPDHAA.Y | -8.38E+00 ± 1.09E+00 | -6.58E+00 ± 2.21E+00 | 1.801 | 2.60E-02 | 9.41E-01 | 4.09E-02 |
| ITIH4 | R.QLGLPGPPDVPDHAAY.H | -9.24E+00 ± 7.94E-01 | -6.43E+00 ± 2.53E+00 | 2.812 | 1.78E-03 | 6.81E-02 | 8.06E-03 |
| ITIH4 | R.QLGLPGPPDVPDHAAYHP.F | -8.88E+00 ± 1.21E+00 | -6.79E+00 ± 1.74E+00 | 2.085 | 1.97E-03 | 9.52E-01 | 8.58E-03 |
| ITIH4 | R.QLGLPGPPDVPDHAAYHPF.R | -2.96E+00 ± 2.18E+00 | -3.64E+00 ± 3.94E+00 | -0.68 | 6.23E-01 | 2.60E-01 | 6.68E-01 |
| ITIH4 | R.QLGLPGPPDVPDHAAYHPFR.R | -6.29E+00 ± 2.61E+00 | -5.52E+00 ± 3.00E+00 | 0.769 | 4.91E-01 | 3.58E-01 | 5.44E-01 |
| ITIH4 | R.RGWNRQAGAAGSRMNFRPGVLSSRQLGLPGPPDVPDHAAYHPF.R | -3.77E+00 ± 3.08E+00 | -6.67E+00 ± 2.63E+00 | -2.907 | 7.47E-03 | 3.21E-01 | 1.74E-02 |
| ITIH4 | S.RQLGLPGPPDVPDHAAYHPF.R | -6.80E+00 ± 2.86E+00 | -5.85E+00 ± 2.19E+00 | 0.95 | 2.96E-01 | 8.96E-01 | 3.57E-01 |
| ITIH4 | S.SRQLGLPGPPDVPDHA.A | -8.55E+00 ± 7.55E-01 | -6.88E+00 ± 1.80E+00 | 1.669 | 1.12E-02 | 8.92E-01 | 2.27E-02 |
| ITIH4 | S.SRQLGLPGPPDVPDHAA.Y | -8.34E+00 ± 1.88E+00 | -7.03E+00 ± 1.29E+00 | 1.308 | 1.99E-02 | 2.45E-01 | 3.38E-02 |
| ITIH4 | S.SRQLGLPGPPDVPDHAAY.H | -9.00E+00 ± 5.75E-01 | -6.34E+00 ± 2.80E+00 | 2.668 | 7.87E-03 | 4.06E-01 | 1.78E-02 |
| ITIH4 | S.SRQLGLPGPPDVPDHAAYHPF.R | -3.51E+00 ± 2.71E+00 | -4.58E+00 ± 3.59E+00 | -1.066 | 4.15E-01 | 3.34E-01 | 4.75E-01 |
| ITIH4 | S.SRQLGLPGPPDVPDHAAYHPFR.R | -8.04E+00 ± 1.67E+00 | -6.34E+00 ± 2.56E+00 | 1.706 | 6.87E-02 | 4.15E-01 | 9.47E-02 |
| ITIH4 | Y.LQGAKIPKPEASFSPR.R | -7.52E+00 ± 2.33E+00 | -5.19E+00 ± 2.76E+00 | 2.329 | 2.41E-02 | 1.16E-01 | 3.86E-02 |
| ITIH4 | Y.YLQGAKIPKPEAS.F | -8.14E+00 ± 1.35E+00 | -7.41E+00 ± 1.60E+00 | 0.727 | 2.28E-01 | 8.24E-01 | 2.82E-01 |
| ITIH4 | Y.YLQGAKIPKPEASFSPR.R | -6.18E+00 ± 2.41E+00 | -6.83E+00 ± 1.84E+00 | -0.649 | 3.93E-01 | 7.79E-01 | 4.53E-01 |
| KNG1 | F.KLDDDLEHQGGHVLDHGH.K | -8.96E+00 ± 4.62E-01 | -6.29E+00 ± 2.66E+00 | 2.677 | 5.28E-03 | 5.71E-01 | 1.47E-02 |
| KNG1 | G.HGLGHGHEQQHGLGH.G | -8.86E+00 ± 1.06E+00 | -6.54E+00 ± 2.73E+00 | 2.316 | 1.68E-02 | 1.64E-01 | 2.99E-02 |
| KNG1 | G.HGLGHGHEQQHGLGHGHKF.K | -8.85E+00 ± 8.31E-01 | -6.66E+00 ± 2.13E+00 | 2.19 | 4.26E-03 | 1.47E-01 | 1.31E-02 |
| KNG1 | H.NLGHGHKHERDQGHGHQ.R | -7.33E+00 ± 2.01E+00 | -7.92E+00 ± 1.12E+00 | -0.587 | 2.64E-01 | 9.14E-01 | 3.23E-01 |
| KNG1 | K.HNLGHGHKHERDQGHGHQ.R | -7.62E+00 ± 2.11E+00 | -5.82E+00 ± 2.75E+00 | 1.797 | 8.11E-02 | 7.14E-01 | 1.10E-01 |
| KNG1 | K.LDDDLEHQGGHVLDHGH.K | -9.07E+00 ± 8.81E-01 | -5.47E+00 ± 3.31E+00 | 3.598 | 2.87E-03 | 4.37E-01 | 1.06E-02 |
| KNG1 | K.LDDDLEHQGGHVLDHGHKH.K | -8.93E+00 ± 8.24E-01 | -5.92E+00 ± 3.01E+00 | 3.015 | 6.07E-03 | 9.53E-01 | 1.56E-02 |
| KNG1 | K.LDDDLEHQGGHVLDHGHKHKHGHGHG.K | -8.95E+00 ± 6.76E-01 | -6.01E+00 ± 3.22E+00 | 2.937 | 1.12E-02 | 7.06E-01 | 2.27E-02 |
| KNG1 | K.RPPGFSPF.R | -2.53E+00 ± 3.13E+00 | -3.68E+00 ± 4.56E+00 | -1.147 | 4.86E-01 | 4.11E-01 | 5.40E-01 |
| KNG1 | L.DDDLEHQGGHVLDHGHKH.K | -9.10E+00 ± 9.57E-01 | -6.55E+00 ± 3.23E+00 | 2.556 | 2.68E-02 | 5.69E-01 | 4.19E-02 |
| KNG1 | R.GHGLGHGHEQQHGLGHGHKF.K | -8.67E+00 ± 6.46E-01 | -4.99E+00 ± 3.77E+00 | 3.68 | 6.44E-03 | 4.00E-01 | 1.61E-02 |
| KNG1 | R.IASFSQNC(+57.02)DIYPGKDFVQPPTKIC(+57.02)VGC(+57.02)PR.D | -9.34E+00 ± 1.05E+00 | -5.45E+00 ± 3.92E+00 | 3.887 | 5.70E-03 | 2.55E-01 | 1.52E-02 |
| LARP1 | I.SDGEEGGGEPGAGGG.A | -7.92E+00 ± 1.63E+00 | -7.48E+00 ± 1.04E+00 | 0.448 | 3.24E-01 | 4.00E-01 | 3.85E-01 |
| MINT | G.GPQGKKGKN.E | -6.59E+00 ± 3.35E+00 | -7.66E+00 ± 2.20E+00 | -1.064 | 2.69E-01 | 8.74E-01 | 3.29E-01 |
| MPRI | S.IC(+57.02)GENENC(+57.02)PPGVG.A | -3.52E+00 ± 6.98E-01 | -6.43E+00 ± 2.59E+00 | -2.908 | 1.40E-03 | 4.57E-02 | 7.14E-03 |
| NETO1 | F.ADGELESM(+15.99)GFSA.R | -3.24E+00 ± 6.37E-01 | -6.25E+00 ± 2.32E+00 | -3.013 | 3.80E-04 | 1.30E-01 | 3.67E-03 |
| PDLI1 | A.QPPSSLVIDKESEVYKML.Q | -7.65E+00 ± 1.46E+00 | -7.63E+00 ± 1.04E+00 | 0.024 | 9.56E-01 | 4.34E-01 | 9.63E-01 |
| PDLI1 | Q.EILESEEKGDPNKPSGF.R | -8.73E+00 ± 1.33E+00 | -7.27E+00 ± 1.40E+00 | 1.454 | 7.81E-03 | 1.66E-01 | 1.78E-02 |
| PON1 | M.AKLIALTLLGMGLALF.R | -8.86E+00 ± 1.10E+00 | -7.15E+00 ± 1.17E+00 | 1.708 | 3.78E-04 | 3.82E-01 | 3.67E-03 |
| PRG4 | E.TAPTTPKGT.A | -6.64E+00 ± 3.31E+00 | -7.25E+00 ± 2.04E+00 | -0.612 | 4.93E-01 | 2.58E-01 | 5.46E-01 |
| QSOX1 | R.AAPGQEPPEHM(+15.99)AELQRNEQEQPLGQWHLS.K | -8.93E+00 ± 4.87E-01 | -6.99E+00 ± 1.43E+00 | 1.939 | 2.44E-04 | 1.40E-01 | 3.26E-03 |
| QSOX1 | R.AAPGQEPPEHMAELQ.R | -9.39E+00 ± 9.49E-01 | -5.53E+00 ± 2.91E+00 | 3.854 | 3.22E-04 | 1.23E-01 | 3.48E-03 |
| QSOX1 | R.AAPGQEPPEHMAELQRNEQEQPLGQWHLS.K | -4.07E+00 ± 6.35E-01 | -4.72E+00 ± 2.83E+00 | -0.647 | 5.09E-01 | 7.85E-01 | 5.59E-01 |
| QSOX1 | R.NEQEQPLGQWHLS.K | -8.93E+00 ± 5.81E-01 | -6.84E+00 ± 1.30E+00 | 2.094 | 4.30E-05 | 8.76E-01 | 9.12E-04 |
| ROCK2 | L.VKKIPKKP.P | -7.31E+00 ± 1.57E+00 | -7.59E+00 ± 1.28E+00 | -0.278 | 5.92E-01 | 9.69E-01 | 6.39E-01 |
| SPTB2 | D.VDDWDNENSSA.R | -6.36E+00 ± 1.71E+00 | -7.23E+00 ± 1.41E+00 | -0.873 | 1.29E-01 | 6.43E-01 | 1.69E-01 |
| SRGN | Q.DLNRIFPL.S | -7.50E+00 ± 1.36E+00 | -7.82E+00 ± 1.10E+00 | -0.322 | 4.66E-01 | 3.53E-01 | 5.22E-01 |
| SRGN | Q.WVRC(+57.02)NPDSNSANC(+57.02)LE.E | -8.77E+00 ± 1.10E+00 | -7.09E+00 ± 1.45E+00 | 1.685 | 2.79E-03 | 7.23E-01 | 1.06E-02 |
| SRGN | Q.WVRC(+57.02)NPDSNSANC(+57.02)LEEKGP.M | -9.32E+00 ± 6.85E-01 | -6.81E+00 ± 1.81E+00 | 2.513 | 2.84E-04 | 7.91E-01 | 3.30E-03 |
| SRGN | Q.WVRC(+57.02)NPDSNSANC(+57.02)LEEKGPM.F | -9.09E+00 ± 1.03E+00 | -7.48E+00 ± 1.65E+00 | 1.604 | 9.63E-03 | 8.64E-01 | 2.03E-02 |
| SRGN | R.ARYQWVRC(+57.02)NPDSNSANC(+57.02)LEEKGPMFELLPGESNKIPRL.R | -9.09E+00 ± 6.49E-01 | -6.71E+00 ± 1.89E+00 | 2.382 | 8.16E-04 | 9.57E-01 | 5.63E-03 |
| SRGN | R.NLPSDSQDLGQHGLEED.F | -8.74E+00 ± 1.18E+00 | -6.43E+00 ± 1.85E+00 | 2.311 | 9.97E-04 | 1.71E-01 | 6.09E-03 |
| SRGN | R.NLPSDSQDLGQHGLEEDF.M | -9.00E+00 ± 7.63E-01 | -7.20E+00 ± 1.54E+00 | 1.807 | 1.85E-03 | 6.50E-01 | 8.32E-03 |
| SRGN | R.NLPSDSQDLGQHGLEEDFM(+15.99)L | -8.98E+00 ± 3.21E-01 | -7.16E+00 ± 1.43E+00 | 1.815 | 5.27E-04 | 1.58E-01 | 4.11E-03 |
| SRGN | R.NLPSDSQDLGQHGLEEDFM.L | -9.55E+00 ± 9.06E-01 | -6.46E+00 ± 1.66E+00 | 3.094 | 2.73E-06 | 6.31E-02 | 1.61E-04 |
| SRGN | R.SLDRNLPSDSQDLGQHGLEED.F | -6.00E+00 ± 2.17E+00 | -6.46E+00 ± 1.77E+00 | -0.467 | 5.13E-01 | 6.04E-01 | 5.63E-01 |
| SRGN | R.SLDRNLPSDSQDLGQHGLEEDF.M | -7.26E+00 ± 1.80E+00 | -6.60E+00 ± 1.31E+00 | 0.663 | 2.27E-01 | 5.14E-01 | 2.82E-01 |
| SRGN | R.SLDRNLPSDSQDLGQHGLEEDFM(+15.99).L | -7.58E+00 ± 1.33E+00 | -6.65E+00 ± 2.16E+00 | 0.932 | 2.23E-01 | 1.69E-01 | 2.79E-01 |
| SRGN | R.SLDRNLPSDSQDLGQHGLEEDFM(+15.99)L | -8.38E+00 ± 1.71E+00 | -7.74E+00 ± 1.64E+00 | 0.636 | 3.15E-01 | 3.93E-01 | 3.75E-01 |
| SRGN | R.SLDRNLPSDSQDLGQHGLEEDFM.L | -4.74E+00 ± 1.84E+00 | -6.21E+00 ± 1.72E+00 | -1.477 | 3.14E-02 | 3.71E-01 | 4.79E-02 |
| SRGN | R.SLDRNLPSDSQDLGQHGLEEDFML | -7.50E+00 ± 2.67E+00 | -7.06E+00 ± 1.54E+00 | 0.441 | 5.32E-01 | 7.28E-01 | 5.80E-01 |
| TBB1 | R.EIVHIQIGQC(+57.02)G.N | -8.70E+00 ± 1.65E+00 | -7.95E+00 ± 1.17E+00 | 0.752 | 1.33E-01 | 6.96E-01 | 1.74E-01 |
| TBB5 | F.SVVPSPKVSDTVVEPYN.A | -7.91E+00 ± 2.00E+00 | -7.98E+00 ± 1.05E+00 | -0.066 | 8.94E-01 | 3.87E-01 | 9.17E-01 |
| THRB | A.TSEYQTFF.N | -7.85E+00 ± 2.10E+00 | -7.82E+00 ± 1.22E+00 | 0.033 | 9.52E-01 | 6.74E-01 | 9.63E-01 |
| THRB | A.TSEYQTFFNPR.T | -5.35E+00 ± 9.16E-01 | -7.13E+00 ± 1.59E+00 | -1.784 | 2.74E-03 | 2.66E-01 | 1.05E-02 |
| THRB | E.DKTERELLESYIDGR.I | -7.02E+00 ± 1.28E+00 | -7.39E+00 ± 1.31E+00 | -0.369 | 4.60E-01 | 5.35E-01 | 5.18E-01 |
| THRB | E.YQTFFNPR.T | -7.10E+00 ± 1.58E+00 | -7.58E+00 ± 1.02E+00 | -0.482 | 2.82E-01 | 7.47E-01 | 3.43E-01 |
| THRB | F.SDYIHPVC(+57.02)LPDRETAASLLQAGYKG.R | -9.33E+00 ± 5.06E-01 | -6.50E+00 ± 2.27E+00 | 2.83 | 3.40E-04 | 1.31E-02 | 3.61E-03 |
| THRB | K.SLEDKTERELLESYIDGR.I | -6.46E+00 ± 1.98E+00 | -2.14E+00 ± 3.12E+00 | 4.326 | 4.06E-04 | 8.64E-01 | 3.71E-03 |
| THRB | L.EDKTERELLESYIDG.R | -8.68E+00 ± 9.03E-01 | -6.26E+00 ± 2.91E+00 | 2.422 | 2.03E-02 | 5.61E-01 | 3.42E-02 |
| THRB | L.FEKKSLEDKTERELLESYIDGR.I | -7.24E+00 ± 1.84E+00 | -7.27E+00 ± 1.48E+00 | -0.026 | 9.65E-01 | 6.85E-01 | 9.71E-01 |
| THRB | P.RTFGSGEADC(+57.02)GLRPLFEKKSLEDKTERELLESYIDGR.I | -7.45E+00 ± 2.45E+00 | -7.80E+00 ± 9.94E-01 | -0.352 | 5.20E-01 | 7.75E-01 | 5.69E-01 |
| THRB | R.IVEGSDAEIGM(+15.99)SPWQVM(+15.99)LF.R | -9.34E+00 ± 6.80E-01 | -7.24E+00 ± 1.97E+00 | 2.098 | 3.91E-03 | 8.54E-01 | 1.24E-02 |
| THRB | R.IVEGSDAEIGMSPWQVM(+15.99)LF.R | -9.19E+00 ± 3.51E-01 | -6.50E+00 ± 2.05E+00 | 2.687 | 4.59E-04 | 8.92E-01 | 3.78E-03 |
| THRB | R.IVEGSDAEIGMSPWQVMLF.R | -8.72E+00 ± 6.27E-01 | -6.47E+00 ± 2.17E+00 | 2.252 | 3.86E-03 | 2.19E-01 | 1.24E-02 |
| THRB | R.TATSEYQTFF.N | -7.30E+00 ± 2.28E+00 | -8.38E+00 ± 9.95E-01 | -1.08 | 4.23E-02 | 3.22E-01 | 6.13E-02 |
| THRB | R.TATSEYQTFFNPR.T | -3.41E+00 ± 1.01E+00 | -5.97E+00 ± 2.15E+00 | -2.566 | 1.49E-03 | 6.02E-01 | 7.14E-03 |
| THRB | R.TATSEYQTFFNPRTFGSGEADC(+57.02)GLRPLFEKKSLEDKTERELLESYIDG.R | -3.34E+00 ± 2.55E+00 | -7.82E+00 ± 1.31E+00 | -4.486 | 1.10E-08 | 2.95E-01 | 2.93E-06 |
| THRB | R.TATSEYQTFFNPRTFGSGEADC(+57.02)GLRPLFEKKSLEDKTERELLESYIDGR.I | 9.17E-01 ± 1.14E+00 | -5.28E+00 ± 2.62E+00 | -6.195 | 3.58E-08 | 2.97E-01 | 6.34E-06 |
| THRB | R.TFGSGEADC(+57.02)GLRPLF.E | -7.76E+00 ± 1.56E+00 | -7.98E+00 ± 1.20E+00 | -0.224 | 6.49E-01 | 5.78E-01 | 6.92E-01 |
| THRB | R.TFGSGEADC(+57.02)GLRPLFE.K | -6.51E+00 ± 1.69E+00 | -4.58E+00 ± 2.42E+00 | 1.928 | 3.23E-02 | 4.35E-01 | 4.87E-02 |
| THRB | R.TFGSGEADC(+57.02)GLRPLFEK.K | -6.99E+00 ± 1.17E+00 | -5.62E+00 ± 1.94E+00 | 1.368 | 5.51E-02 | 6.58E-01 | 7.80E-02 |
| THRB | R.TFGSGEADC(+57.02)GLRPLFEKKSLE.D | -7.92E+00 ± 1.83E+00 | -7.48E+00 ± 1.49E+00 | 0.439 | 4.65E-01 | 5.30E-01 | 5.22E-01 |
| THRB | R.TFGSGEADC(+57.02)GLRPLFEKKSLEDKTE.R | -7.59E+00 ± 1.19E+00 | -7.71E+00 ± 1.07E+00 | -0.124 | 7.69E-01 | 7.79E-01 | 8.04E-01 |
| THRB | R.TFGSGEADC(+57.02)GLRPLFEKKSLEDKTERELLESYIDG.R | -2.50E+00 ± 4.25E+00 | -6.25E+00 ± 2.89E+00 | -3.752 | 4.17E-03 | 9.10E-01 | 1.29E-02 |
| THRB | R.TFGSGEADC(+57.02)GLRPLFEKKSLEDKTERELLESYIDGR.I | 3.41E+00 ± 6.29E-01 | -1.69E+00 ± 3.74E+00 | -5.102 | 2.85E-04 | 7.85E-01 | 3.30E-03 |
| THRB | S.EYQTFF.N | -7.02E+00 ± 2.27E+00 | -7.80E+00 ± 9.40E-01 | -0.776 | 1.28E-01 | 2.75E-01 | 1.68E-01 |
| THRB | S.EYQTFFNPR.T | -5.01E+00 ± 1.21E+00 | -6.50E+00 ± 1.69E+00 | -1.489 | 1.89E-02 | 4.58E-01 | 3.23E-02 |
| THRB | T.ATSEYQTFFNPR.T | -7.42E+00 ± 1.49E+00 | -7.47E+00 ± 1.36E+00 | -0.05 | 9.25E-01 | 9.07E-01 | 9.42E-01 |
| THRB | T.SEYQTFF.N | -7.30E+00 ± 1.74E+00 | -7.94E+00 ± 1.29E+00 | -0.641 | 2.37E-01 | 8.06E-01 | 2.94E-01 |
| THRB | T.SEYQTFFNPR.T | -6.53E+00 ± 1.61E+00 | -7.41E+00 ± 1.29E+00 | -0.879 | 9.43E-02 | 2.91E-01 | 1.27E-01 |
| THRB | V.TGWGNLKETWTAN.V | -9.00E+00 ± 7.15E-01 | -7.21E+00 ± 1.79E+00 | 1.79 | 4.52E-03 | 5.11E-02 | 1.36E-02 |
| TLN1 | S.MPPAQQQITSGQMH.R | -8.37E+00 ± 1.40E+00 | -8.04E+00 ± 9.66E-01 | 0.332 | 4.19E-01 | 5.08E-01 | 4.78E-01 |
| TSP1 | P.SSPAFRIED.A | -9.08E+00 ± 8.51E-01 | -6.01E+00 ± 3.45E+00 | 3.067 | 1.28E-02 | 3.96E-01 | 2.48E-02 |
| TTHY | A.GPTGTGESKC(+57.02)PLMVKVLDAVRGSPAINVAVHVF.R | -9.24E+00 ± 7.75E-01 | -3.86E+00 ± 4.05E+00 | 5.381 | 2.05E-04 | 3.93E-02 | 2.94E-03 |
| TTHY | A.GPTGTGESKC(+57.02)PLMVKVLDAVRGSPAINVAVHVFR.K | -8.34E+00 ± 8.98E-01 | -4.89E+00 ± 3.99E+00 | 3.454 | 1.36E-02 | 1.64E-01 | 2.58E-02 |
| TTHY | K.ALGISPFHEHAEVVFTANDSGPRRYTIAALLSPYSYSTTAVVTNPKE | -8.57E+00 ± 2.52E+00 | -5.09E+00 ± 4.26E+00 | 3.481 | 2.37E-02 | 1.67E-01 | 3.83E-02 |
| TTHY | K.TSESGELHGLTTEEEFVEGIYKVEIDTKSYWK.A | -8.98E+00 ± 8.07E-01 | -6.00E+00 ± 3.19E+00 | 2.984 | 9.55E-03 | 5.86E-01 | 2.03E-02 |
| TTHY | N.DSGPRRYTIAALLSPYSYSTTAVVTNPKE | -7.57E+00 ± 1.59E+00 | -7.33E+00 ± 2.03E+00 | 0.237 | 7.51E-01 | 5.44E-01 | 7.88E-01 |
| TTHY | P.TGTGESKC(+57.02)PLMVKVLDAVRGSPAINVAVHVF.R | -7.87E+00 ± 2.24E+00 | -4.85E+00 ± 4.12E+00 | 3.021 | 3.53E-02 | 5.19E-02 | 5.25E-02 |
| TTHY | P.TGTGESKC(+57.02)PLMVKVLDAVRGSPAINVAVHVFR.K | -8.73E+00 ± 1.19E+00 | -5.07E+00 ± 3.77E+00 | 3.659 | 6.66E-03 | 2.25E-01 | 1.61E-02 |
| TTHY | R.KAADDTWEPFASGKTSESGELHGLTTEEEFVEGIYK.V | -9.01E+00 ± 6.62E-01 | -5.90E+00 ± 3.62E+00 | 3.115 | 1.61E-02 | 9.27E-01 | 2.92E-02 |
| TTHY | R.KAADDTWEPFASGKTSESGELHGLTTEEEFVEGIYKVEIDTK.S | -8.34E+00 ± 2.30E+00 | -6.33E+00 ± 3.06E+00 | 2.011 | 7.59E-02 | 3.20E-01 | 1.04E-01 |
| TTHY | R.RYTIAALLSPYSYSTTAVVTNPKE | -7.85E+00 ± 2.22E+00 | -4.74E+00 ± 3.63E+00 | 3.107 | 1.77E-02 | 1.18E-01 | 3.08E-02 |
| TTHY | R.YTIAALLSPYSYSTTAVVTNPKE | -8.66E+00 ± 7.89E-01 | -6.69E+00 ± 2.61E+00 | 1.972 | 3.45E-02 | 9.16E-01 | 5.18E-02 |
| TYB4 | E.KNPLPSKETIEQEKQAGES | -5.88E+00 ± 1.92E+00 | -5.41E+00 ± 1.82E+00 | 0.473 | 5.05E-01 | 8.09E-01 | 5.56E-01 |
| TYB4 | E.TQEKNPLPSKETIEQEKQAGES | -6.35E+00 ± 1.61E+00 | -6.80E+00 ± 1.48E+00 | -0.449 | 4.37E-01 | 5.43E-01 | 4.96E-01 |
| TYB4 | K.KTETQEKNPLPSKETIEQEKQAGES | -8.21E+00 ± 1.83E+00 | -5.30E+00 ± 1.32E+00 | 2.907 | 4.73E-06 | 4.96E-01 | 2.09E-04 |
| TYB4 | K.TETQEKNPLPSKETI.E | -9.20E+00 ± 8.72E-01 | -6.63E+00 ± 1.77E+00 | 2.573 | 1.91E-04 | 9.38E-01 | 2.83E-03 |
| TYB4 | K.TETQEKNPLPSKETIEQEKQAGES | -8.72E+00 ± 8.46E-01 | -5.02E+00 ± 1.67E+00 | 3.703 | 1.79E-07 | 4.30E-01 | 2.37E-05 |
| TYB4 | T.QEKNPLPSKETIEQEKQAGES | -6.96E+00 ± 2.25E+00 | -7.41E+00 ± 1.32E+00 | -0.442 | 4.58E-01 | 4.17E-01 | 5.16E-01 |
| VTNC | A.DQESC(+57.02)KGRC(+57.02)TEGFNVDKKC(+57.02)QC(+57.02) DELC(+57.02)SYYQSC(+57.02)C(+57.02)TDYTAEC(+57.02)KPQVT.R | -5.70E+00 ± 3.52E+00 | -3.10E+00 ± 5.03E+00 | 2.607 | 1.59E-01 | 6.56E-01 | 2.04E-01 |
| VTNC | A.DQESC(+57.02)KGRC(+57.02)TEGFNVDKKC(+57.02)QC(+57.02) DELC(+57.02)SYYQSC(+57.02)C(+57.02)TDYTAEC(+57.02)KPQVTR.G | -7.87E+00 ± 1.97E+00 | -5.19E-01 ± 3.43E+00 | 7.349 | 4.35E-07 | 4.15E-01 | 4.62E-05 |
| VTNC | A.DQESC(+57.02)KGRC(+57.02)TEGFNVDKKC(+57.02)QC(+57.02) DELC(+57.02)SYYQSC(+57.02)C(+57.02)TDYTAEC(+57.02)KPQVTRG.D | -9.10E+00 ± 6.78E-01 | -6.41E+00 ± 2.26E+00 | 2.691 | 1.36E-03 | 9.98E-01 | 7.14E-03 |
| VTNC | A.DQESC(+57.02)KGRC(+57.02)TEGFNVDKKC(+57.02)QC(+57.02) DELC(+57.02)SYYQSC(+57.02)C(+57.02)TDYTAEC(+57.02)KPQVTRGDVF.T | -6.82E+00 ± 1.90E+00 | -6.72E+00 ± 1.50E+00 | 0.099 | 8.71E-01 | 7.46E-01 | 8.98E-01 |
| VTNC | Q.ESC(+57.02)KGRC(+57.02)TEGFNVDKKC(+57.02)QC(+57.02)DELC (+57.02)SYYQSC(+57.02)C(+57.02)TDYTAEC(+57.02)KPQVT.R | -9.27E+00 ± 1.16E+00 | -7.02E+00 ± 1.80E+00 | 2.257 | 1.10E-03 | 3.93E-01 | 6.45E-03 |
| ZYX | F.HVQPQPQPKPQVQLHVQSQT.Q | -6.48E+00 ± 1.74E+00 | -7.05E+00 ± 1.42E+00 | -0.57 | 3.24E-01 | 7.84E-01 | 3.85E-01 |
| ZYX | K.VNPFRPGDSEPPPAPGAQRAQ.M | -6.98E+00 ± 1.79E+00 | -7.07E+00 ± 1.03E+00 | -0.092 | 8.43E-01 | 4.47E-01 | 8.73E-01 |
| ZYX | S.LANTQPRGPPASSPAPAPKFSPVTPKFTPVAS.K | -6.93E+00 ± 1.80E+00 | -7.34E+00 ± 1.40E+00 | -0.41 | 4.75E-01 | 6.75E-01 | 5.31E-01 |

*Data are represented (log 2 transformed) as mean ± standard deviation. Statistical significance was calculated using a two-way ANOVA controlling for presence of type 2 diabetes mellitus (T2DM)* *as covariate. A p-value <0.05 (Benjamini–Hochberg false discovery rate) was considered significant.*


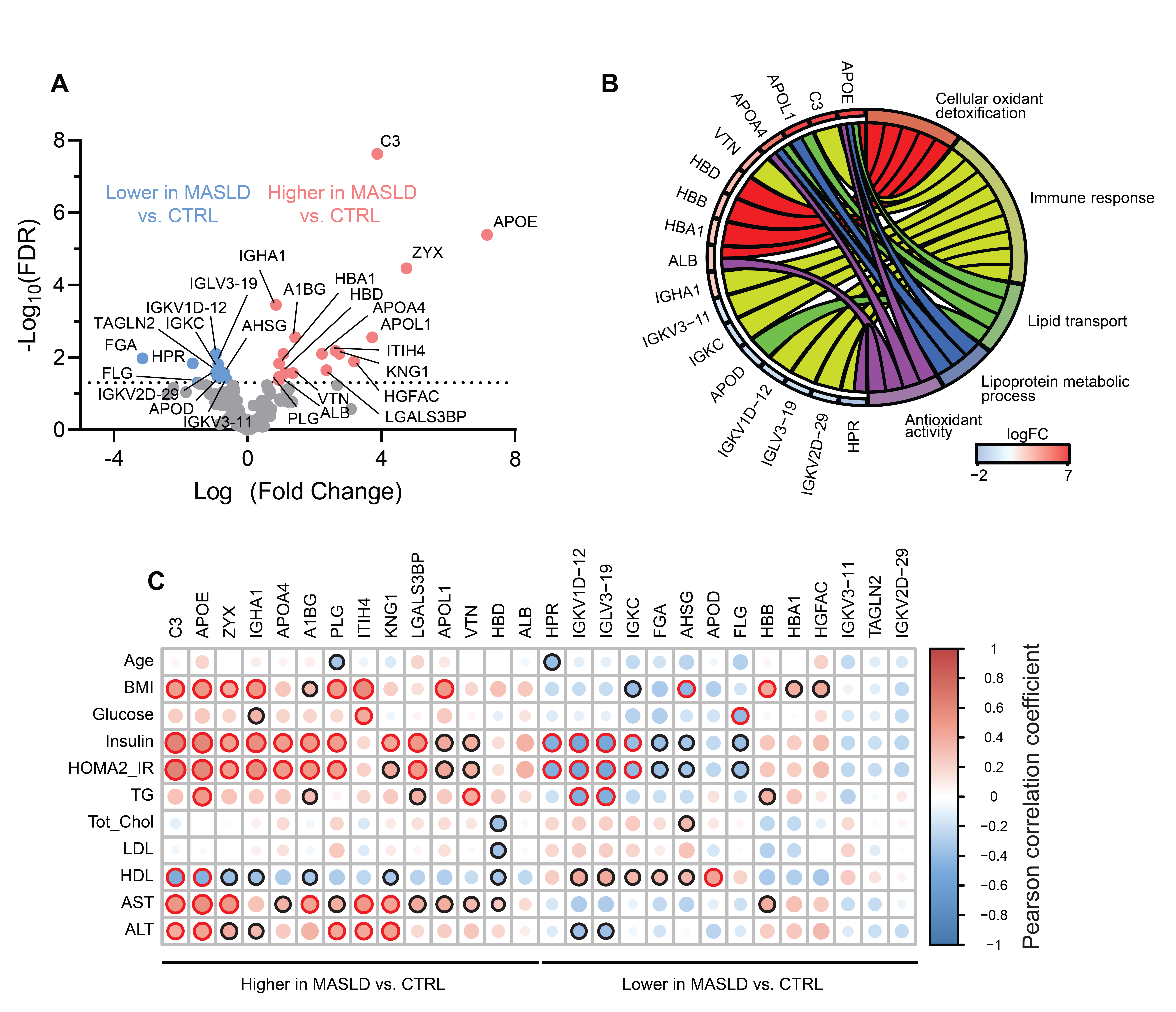


**Supplementary Figure 1. Untargeted serum proteomics in healthy volunteers and patients with MASLD.**

(A) Volcano plot showing differences in serum proteins of MASLD and controls in the untargeted cohort (n = 41). Statistical significance was assessed using two-way ANOVA using the disease state and presence of type 2 diabetes mellitus as covariates; a p-value <0.05 (Benjamini–Hochberg false discovery rate) was considered significant. In Supplementary Table 7 are reported all the protein analysed with raw and adjusted p-values. (B) Functional annotation enrichment analysis of the proteins highlighted in (A). (C) Heatmap representing a correlation matrix among significantly different serum proteins and clinical data in healthy volunteers and MASLD patients: colour represents the Pearson correlation coefficient (red: positive; blue: negative), and the size of the circle represents significance (black bold borders highlight correlations with p<0.05; red bold borders highlight correlations with p<0.01).


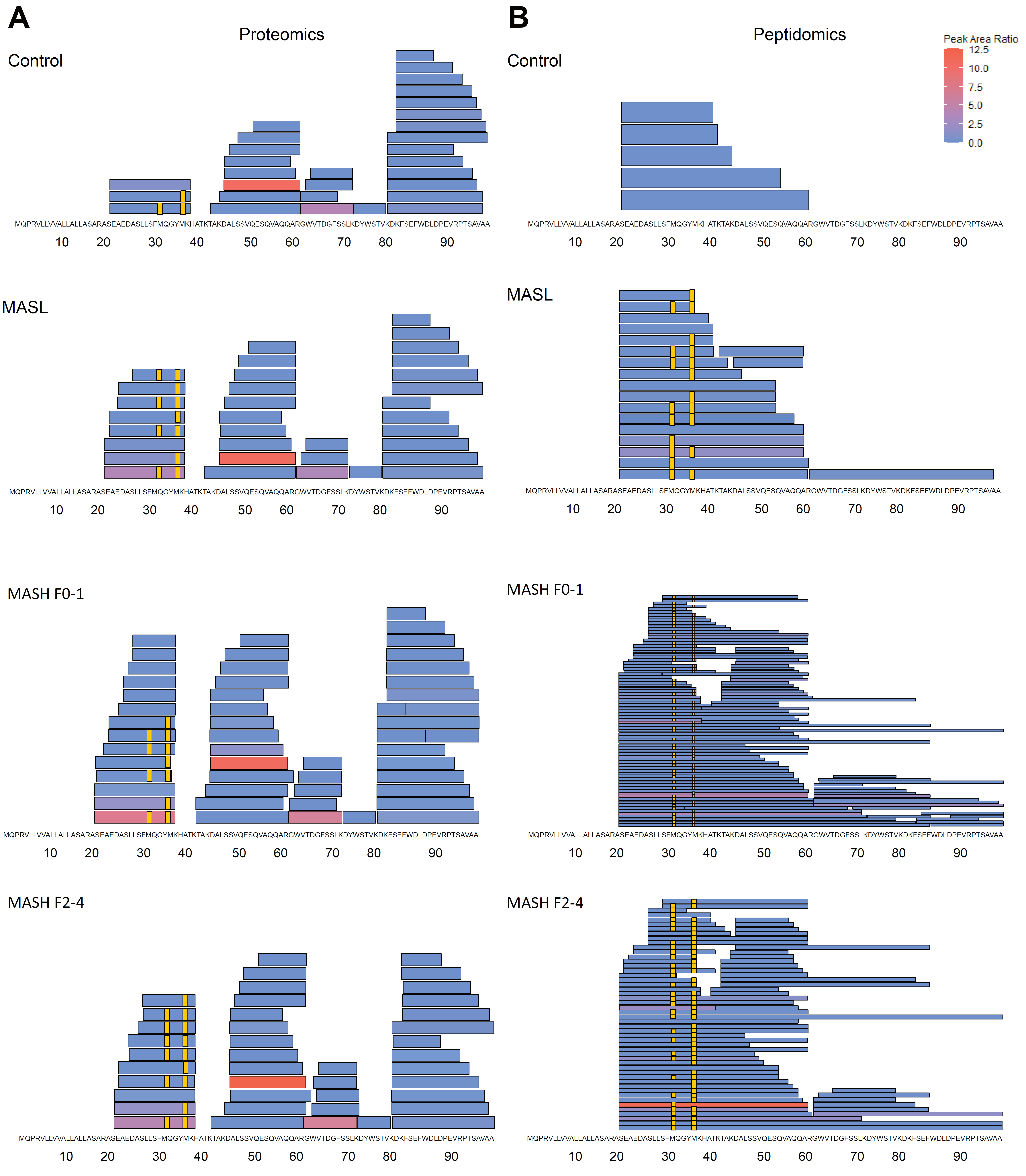


**Supplementary Figure 2. Peptides derived from Apolipoprotein C-III identified in control or MASLD samples of varying disease stages.** Fill colour is peptide peak area ratio (log scale). Yellow boxes indicate methionine oxidations. A) Peptides identified by proteomics, where trypsin was used to cleave at the C terminus of lysine and arginine with high efficiency and specificity, producing analogous peptides of optimal length and charge for MS analysis. B) Peptides identified using an intact peptidomics approach, identifying endogenous peptide fragments identified only in disease samples.


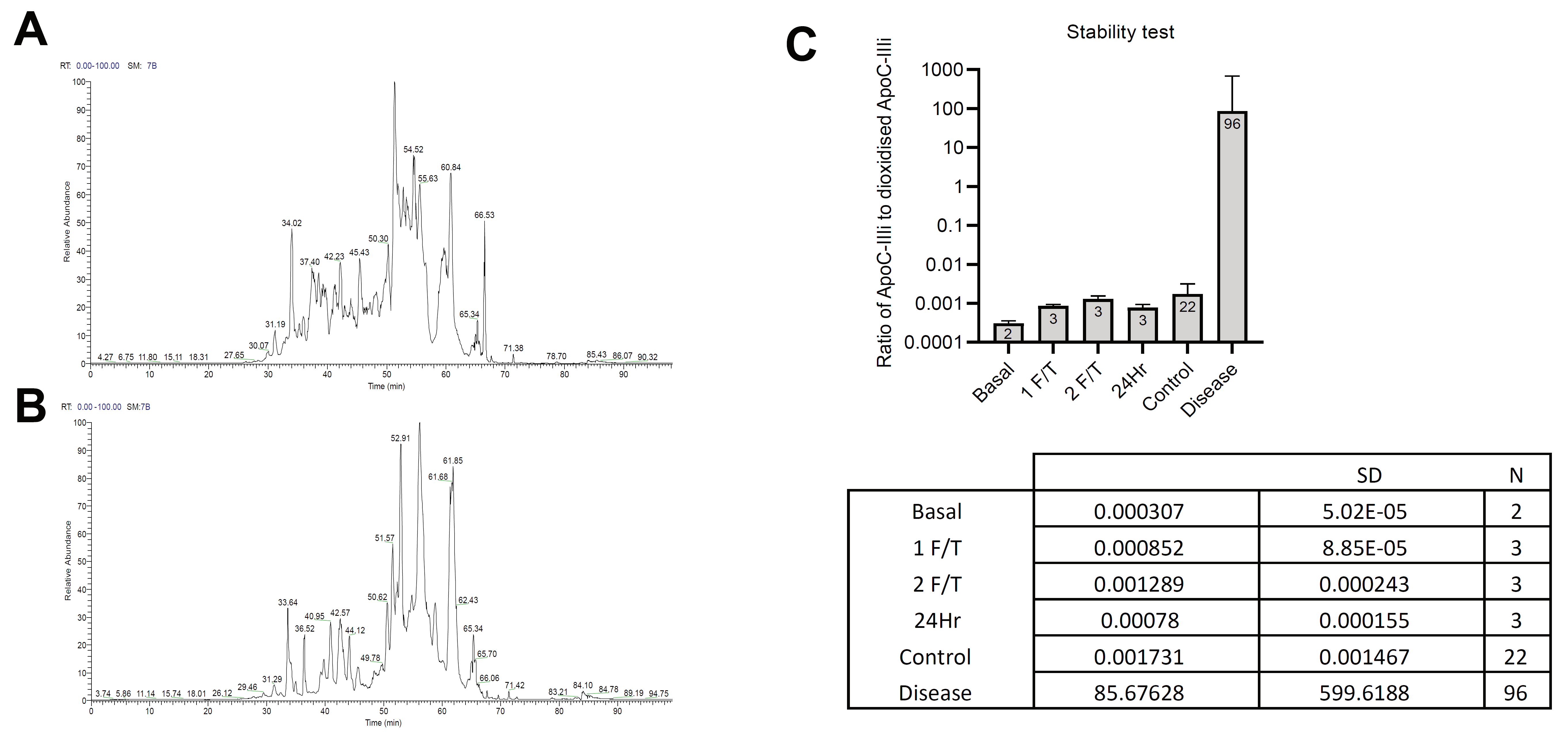


**Supplementary Figure 3. Validation of in-vivo and ex-vivo oxidation of ApoC-III isoforms in peptidomics analysis.** Total ion chromatograms of MASLD serum A) showing increased peptide content from 30 to 50 minutes compared with control serum (B). C) Peak area ratios of ApoC-III_i_ to dioxidised ApoC-III_i_ during a stability test. F/T = freeze-thaw cycle.





**Supplementary Figure 4.** Targeted serum peptidomics in healthy volunteers and patients with MASLD. (A-H) ApoC-III 21-59, ApoE 281-317 and their oxidised forms alongside their ratios (oxidised to non-oxidised forms) in MASLD compared to controls in the validation cohort (n = 107). Statistical significance was assessed using three-way ANOVA using the disease state, presence of type 2 diabetes (T2DM) and sex as covariates; a p-value <0.05 was considered significant. (I) Heatmap representing a correlation matrix among selected serum peptides and clinical data in healthy volunteers and MASLD patients: colour represents the Pearson correlation coefficient (red: positive; blue: negative), and the size of the circle represents significance (black bold borders highlight correlations with p<0.05; red bold borders highlight correlations with p<0.01).

**Supplementary references**

1. R. G. Kay *et al.*, Mass spectrometric characterisation of the circulating peptidome following oral glucose ingestion in control and gastrectomised patients. *Rapid communications in mass spectrometry : RCM* **34**, e8849 (2020).
